# Corrigendum: Seed coat pattern QTL and development in cowpea (*Vigna unguiculata* [L.] Walp.)

**DOI:** 10.3389/fpls.2023.1299051

**Published:** 2023-11-01

**Authors:** Ira A. Herniter, Ryan Lo, María Muñoz-Amatriaín, Sassoum Lo, Yi-Ning Guo, Bao-Lam Huynh, Mitchell Lucas, Zhenyu Jia, Philip A. Roberts, Stefano Lonardi, Timothy J. Close

**Affiliations:** ^1^ Department of Botany and Plant Sciences, University of California, Riverside, CA, United States; ^2^ Department of Nematology, University of California, Riverside, CA, United States; ^3^ Department of Computer Sciences and Engineering, University of California, Riverside, CA, United States

**Keywords:** *Vigna unguiculata*, cowpea, seed coat, pigment, pattern, quantitative trait loci

In the published article, there was an error. Several additional characters were added to a subsection title in the middle of a word.

A correction has been made to **Results, Identification of Candidate Genes**, *Identification of Candidate Genes*, page 5. This subsection title previously stated:

“I890-dentification of Candidate Genes”

The corrected sentence appears below:

“Identification of Candidate Genes”

The authors apologize for this error and state that this does not change the scientific conclusions of the article in any way. The original article has been updated.

