# Supplementary material for: Seed Coat Pattern QTL and Development in Cowpea (Vigna unguiculata [L.] Walp.)
Source: Front Plant Sci. 2019 Oct 25;10:1346. doi: 10.3389/fpls.2019.01346 (PMC6824211; doi:10.3389/fpls.2019.01346)
Supplement: Supplementary file 2 [file DataSheet_1.pdf]

```
Tue Mar 12, 2019 16:10 PDT
Cowpea1.0_Vu07(old2)_20533755_20546000.fa from 1 to 12246
Alignment to
CowpeaA_LG7_19158077-19171400.fa--  Matches:12012; Mismatches:116; Gaps:1314; Unattempted:0
CowpeaB_LG7_18768344-18781459.fa--  Matches:11705; Mismatches:98; Gaps:1756; Unattempted:0
CowpeaC_LG7_18103281-18116000.fa--  Matches:11488; Mismatches:104; Gaps:1782; Unattempted:0
CowpeaD_LG7_18460739-18472000.fa--  Matches:9805; Mismatches:82; Gaps:1347; Unattempted:2270

               *         *         *         *         *         *         *         *         *         *
     1>TCGGTGGTTGGTTGTTTTGAAGCGTAGTACTAGAGGAAGAAAGAACACCTTCATTGCGAATATTCTTAGAAATATAATGTAAAATAATATCCTAGATTAT>100
     1>TCGGTGGTTGGTTGTTTTGAAGCGTAGTACTAGAGGAAGAAAGAACACCTTCATTGCGAATATTCTTAGAAATATAATGTAAAATAATATCCTAGATTAT>100
     1>TCGGTGGTTGGTTGTTTTGAAGCGTAGTACTAGAGGAAGAAAGAACACCTTCATTGCGAATATTCTTAGAAATATAATGTAAAATAATATCCTAGATTAT>100
     1>TCGGTGGTTGGTTGTTTTGAAGCGTAGTACTAGAGGAAGAAAGAACACCTTCATTGCGAATATTCTTAGAAATATAATGTAAAATAATATCCTAGATTAT>100
     1>TCGGTGGTTGGTTGTTTTGAAGAGTAGTACTAGAGGAAGAAAGAACAGCTTCATTGCGAATATTCTTAGAAATATAATGTAAAATAATATCCTAGATTAT>100

               *         *         *         *         *         *         *         *         *         *
   101>TGTTAGATCATACATAATTATTATCACACTAAATAGAATGTGAACTAGTTTAAGTAATAATAACTAAAACTCTAATGAGAGCAGTGGTTCCTAGTTTGTT>200
   101>TGTTAGATCATACATAATTATTATCACACTAAATAGAATGTGAACTAGTTTAAGTAATAATAACTAAAACTCTAATGAGAGCAGTGGTTCCTAGTTTGTT>200
   101>TGTTAGATCATACATAATTATTATCACACTAAATAGAATGTGAACTAGTTTAAGTAATAATAACTAAAACTCTAATGAGAGCAGTGGTTCCTAGTTTGTT>200
   101>TGTTAGATCATACATAATTATTATCACACTAAATAGAATGTGAACTAGTTTAAGTAATAATAACTAAAACTCTAATGAGAGCAGTGGTTCCTAGTTTGTT>200
   101>TGTTAGATCATACATAATTATTATCACACTAAATAGAATGTGAACTAGTTTAAGTAATAATAACTAAAACTCTAATGAGAGCAGTGGTTCCTAGTTTGTT>200

               *         *         *         *         *         *         *         *         *         *
   201>TTTGTTGATCAGGCTTTCTGAGAATGCTTATGGGGATCACATTTGGCTTCAATCAATCTTAGATACACATTAAGCCATTGTGATTAGATATTACAAGAAA>300
   201>TTTGTTGATCAGGCTTTCTGAGAATGCTTATGGGGATCACATTTGGCTTCAATCAATCTTAGATACACATTAAGCCATTGTGATTAGATATTACAAGAAA>300
   201>TTTGTTGATCAGGCTTTCTGAGAATGCTTATGGGGATCACATTTGGCTTCAATCAATCTTAGATACACATTAAGCCATTGTGATTAGATATTACAAGAAA>300
   201>TTTGTTGATCAGGCTTTCTGAGAATGCTTATGGGGATCACATTTGGCTTCAATCAATCTTAGATACACATTAAGCCATTGTGATTAGATATTACAAGAAA>300
   201>TTTGTTGATCAGGCTTTCTGAGAATGCTTATGGGGATCACATTTGGCTTCAATCAATCTTAGATACACATTAAGCCATTGTGATTAGATATTACAAGAAA>300

               *         *         *         *         *         *         *         *         *         *
   301>AAAATAATTAAAGAGAAGTTAATCCATAAAGTTAATTAAGGCATGAAAATTAGAAGAGTGGTATTCAGTGGAGAGGATGGGGAATGATTTGGTTAAGGGC>400
   301>AAAATAATTAAAGAGAAGTTAATCCATAAAGTTAATTAAGGCATGAAAATTAGAAGAGTGGTATTCAGTGGAGAGGATGGGGAATGATTTGGTTAAGGGC>400
   301>AAAATAATTAAAGAGAAGTTAATCCATAAAGTTAATTAAGGCATGAAAATTAGAAGAGTGGTATTCAGTGGAGAGGATGGGGAATGATTTGGTTAAGGGC>400
   301>AAAATAATTAAAGAGAAGTTAATCCATAAAGTTAATTAAGGCATGAAAATTAGAAGAGTGGTATTCAGTGGAGAGGATGGGGAATGATTTGGTTAAGGGC>400
   301>AAAATAATTAAAGAGAAGTTAATCCATAAAGTTAATTAAGGCATGAAAATTAGAAGAGTGGTATTCAGTGGAGAGGATGGGGAATGATTTGGTTAAGGGC>400

               *         *         *         *         *         *         *         *         *         *
   401>CTTCTTCACTTCCACAATGCTCACCTTCTTCCCGTTCGCATGTTCCTTCACCTTAGCCCTCAACTCCGCCATGAACACGCCATTGCTAAGTGATGACTGC>500
   401>CTTCTTCACTTCCACAATGCTCACCTTCTTCCCGTTCGCATGTTCCTTCACCTTAGCCCTCAACTCCGCCATGAACACGCCATTGCTAAGTGATGACTGC>500
   401>CTTCTTCACTTCCACAATGCTCACCTTCTTCCCGTTCGCATGTTCCTTCACCTTAGCCCTCAACTCCGCCATGAACACGCCATTGCTAAGTGATGACTGC>500
   401>CTTCTTCACTTCCACAATGCTCACCTTCTTCCCGTTCGCATGTTCCTTCACCTTAGCCCTCAACTCCGCCATGAACACGCCATTGCTAAGTGATGACTGC>500
   401>CTTCTTCACTTCCACAATGCTCACCTTCTTCCCGTTTGCATGTTCCTTCACCTTAGCCCTCAACTCCGCCATGAACACGCCATTGCTAAGTGATGACTGC>500

               *         *         *         *         *         *         *         *         *         *
   501>ACCCCAATAACCTCTATTCTCACCTCCCTCAGCATCTGCATCACATCAAGAAGCAACCCTTCTCTGTGGACGCACTCGATCTCCAACAGTGCGTCGCTCT>600
   501>ACCCCAATAACCTCTATTCTCACCTCCCTCAGCATCTGCATCACATCAAGAAGCAACCCTTCTCTGTGGACGCACTCGATCTCCAACAGTGCGTCGCTCT>600
   501>ACCCCAATAACCTCTATTCTCACCTCCCTCAGCATCTGCATCACATCAAGAagcaacccttcTCTGTGGACGCACTCGATCTCCAACAGTGCGTCGCTCT>600
   501>ACCCCAATAACCTCTATTCTCACCTCCCTCAGCATCTGCATCACATCAAGAAGCAACCCTTCTCTGTGGACGCACTCGATCTCCAACAGTGCGTCGCTCT>600
   501>ACCCCAATAACCTCTATTCTCACCTCCCTCAGCATCTGCATCACATCAAGAAGCAACCCTTCTCTGTGGACGCACTCGATCTCCAACAGTGCGTCGCTCT>600

               *         *         *         *         *         *         *         *         *         *
   601>CAATGATCGAAACTTGCACTGATGTTGCCGTCTCAGCCTCCACCGCCGTCGCCTTCGCCGCCACCCCTTCCACGATCCTCACCTTCCTCTTCTCCATCAC>700
   601>CAATGATCGAAACTTGCACTGATGTTGCCGTCTCAGCCTCCACCGCCGTCGCCTTCGCCGCCACCCCTTCCACGATCCTCACCTTCCTCTTCTCCATCAC>700
   601>CAATGATCGAAACTTGCACTGATGTTGCCGTCTCAGCCTCCACCGCCGTCGCCTTCGCCGCCACCCCTTCCACGATCCTCACCTTCCTCTTCTCCATCAC>700
   601>CAATGATCGAAACTTGCACTGATGTTGCCGTCTCAGCCTCCACCGCCGTCGCCTTCGCCGCCACCCCTTCCACGATCCTCACCTTCCTCTTCTCCATCAC>700
   601>CAATGATCGAAACTTGCACTGATGTTGCCGTCTCAGCCTCCACCGCCGTCGCCTTCGCCGCCACCCCTTCCACGATCCTCACCTTCCTCTTCTCCATCAC>700

               *         *         *         *         *         *         *         *         *         *
   701>CGTCACCCCACTCCTCTGCTGCTCCTTGCTCGAATTGCTGCTCGTCCTTTGAACCGTCACCTATCCACATTTGTTAACGCATCAACAAATCATGTCACCA>800
   701>CGTCACCCCACTCCTCTGCTGCTCCTTGCTCGAATTGCTGCTCGTCCTTTGAACCGTCACCTATCCACATTTGTTAACGCATCAACAAATCATGTCACCA>800
   701>CGTCACCCCACTCCTCTGCTGCTCCTTGCTCGAATTGCTGCTCGTCCTTTGAACCGTCACCTATCCACATTTGTTAACGCATCAACAAATCATGTCACCA>800
   701>CGTCACCCCACTCCTCTGCTGCTCCTTGCTCGAATTGCTGCTCGTCCTTTGAACCGTCACCTATCCACATTTGTTAACGCATCAACAAATCATGTCACCA>800
   701>CGTCACCCCACTCCTCTGCTGCTCCTTGCTCGAATTGCTGCTCGTCCTTTGAACCGCCACCTATCCACATTTGTTAACGCATCAACAAATCATGTCACCA>800

               *         *         *         *         *         *         *         *         *         *
   801>AAATATATAAACTTTATTAATTCTAATAGGGATAATGTCCTTGTTTTTCTTCATTCTAGAATCACCTCTGGGAGCTTGGAACGGTGTTCCGCCTCAGTGT>900
   801>AAATATATAAACTTTATTAATTCTAATAGGGATAATGTCCTTGTTTTTCTTCATTCTAGAATCACCTCTGGGAGCTTGGAACGGTGTTCCGCCTCAGTGT>900
   801>AAATATATAAACTTTATTAATTCTAATAGGGATAATGTCCTTGTTTTTCTTCATTCTAGAATCACCTCTGGGAGCTTGGAACGGTGTTCCGCCTCAGTGT>900
   801>AAATATATAAACTTTATTAATTCTAATAGGGATAATGTCCTTGTTTTTCTTCATTCTAGAATCACCTCTGGGAGCTTGGAACGGTGTTCCGCCTCAGTGT>900
   801>AAATATATAAACTTTATTAATTATAATAGGGATAATGTCGATTTTTTTCTTGATTCTAGAATCACCTCTGGGAGCTTGGAACGGTGTTCCGCCTCAGTGT>900
```

```
              *         *         *         *         *         *         *         *         *         *
 901>GGCGGTTACGCGCCTCCAGCTCCTGGATCTTCCGCCGCAGCTGCTTCACATACTCGATGGTGTCACCCAGAATGGATGCCTTGTCCATTTTGGTGACGAA>1000
 901>GGCGGTTACGCGCCTCCAGCTCCTGGATCTTCCGCCGCAGCTGCTTCACATACTCGATGGTGTCACCCAGAATGGATGCCTTGTCCATTTTGGTGACGAA>1000
 901>GGCGGTTACGCGCCTCCAGCTCCTGGATCTTCCGCCGCAGCTGCTTCACATACTCGATGGTGTCACCCAGAATGGATGCCTTGTCCATTTTGGTGACGAA>1000
 901>GGCGGTTACGCGCCTCCAGCTCCTGGATCTTCCGCCGCAGCTGCTTCACATACTCGATGGTGTCACCCAGAATGGATGCCTTGTCCATTTTGGTGACGAA>1000
 901>GGCGGTTACGCGCCTCCAGCTCCTGGATCTTCCGCCGCAGCTGCTTCACATACTCGATGGTGTCACCCAGAATGGATGCCTTGTCCATTTTGGTGACGAA>1000

              *         *         *         *         *         *         *         *         *         *
1001>GGGGACCAGTGACCGCAGGATTACGAACCGCTCGTTCAGCTTCTCGCGGCGCCGGCGCTCCGCGAGCACGTGGTTGGCGCTCAGCTCATCCTGCGGCGTT>1100
1001>GGGGACCAGTGACCGCAGGATTACGAACCGCTCGTTCAGCTTCTCGCGGCGCCGGCGCTCCGCGAGCACGTGGTTGGCGCTCAGCTCATCCTGCGGCGTT>1100
1001>GGGGACCAGTGACCACAGGATTACGAACCGCTCGTTCAGCTTCTCGCGGCGCCGGCGCTCCGCGAGCACGTGGTTGGCGCTCAGCTCGTCCTGCGGCGTT>1100
1001>GGGGACCAGTGACCGCAGGATTACGAACCGCTCGTTCAGCTTCTCGCGGCGCCGGCGCTCCGCGAGCACGTGGTTGGCGCTCAGCTCGTCCTGCGGCGTT>1100
1001>GGGGACCAGTGACCGCAGGATTACGAACCGCTCGTTCAGCTTCTCGCGGCGCCGGCGCTCCGCGAGCACGTGGTTGGCGCTCAGCTCGTCCTGCGGCGTT>1100

              *         *         *         *         *         *         *         *         *         *
1101>CCCTTGCCGCGCAGCTTGGGGTCGGCGGCGGTTTGGGGGGAAGTGTCGGCGGGGTTCTTGGCGTGTAGATAGGGGACGGTGAAGAGAATGTACTTGAGGA>1200
1101>CCCTTGCCGCGCAGCTTGGGGTCGGCGGCGGTTTGGGGGGaagtgtcggcGGGGTTCTTGGCGTGTAGATAGGGGACGGTGAAGAGAATGTACTTGAGGA>1200
1101>CCCTTGCCGCGCAGCTTGGGGTCGGCGGCGGTTTGGGGGGAAGTGTCGGCGGGGTTCTTGGCGTGTAGATAGGGGACGGTGAAGAGAATGTACTTGAGGA>1200
1101>CCCTTGCCGCGCAGCTTGGGGTCGGCGGCGGTTTGGGGGGAAGTGTCGGCGGGGTTCTTGGCGTGTAGATAGGGGACGGTGAAGAGAATGTACTTGAGGA>1200
1101>CCCTTGCCGCGCAGCTTGGGGTCGGCGGCGGTTTGGGGGGAAGTGTCGGCGGGGTTCTTGGCGTGTAGATAGGGGACGGTGAAGAGAATGTACTTGAGGA>1200

              *         *         *         *         *         *         *         *         *         *
1201>GCCACTGGCTGGGGCCGTCCACCGCCGGGTGGAAGAGGTGGCTGGCGCGGCTGCTCCACTTTGTGAAAGCTGAATGCGTGGAGTATGTGATGTAGCCAAC>1300
1201>GCCACTGGCTGGGGCCGTCCACCGCCGGGTGGAAGAGGTGGCTGGCGCGGCTGCTCCACTTTGTGAAAGCTGAATGCGTGGAGTATGTGATGTAGCCAAC>1300
1201>GCCACTGGCTGGGGCCGTCCACCGCCGGGTGGAAGAGGTGGCTGGCGCGGCTGCTCCACTTTGTGAAAGCTGAATGCGTGGAGTATGTGATGTAGCCAAC>1300
1201>GCCACTGGCTGGGGCCGTCCACCGCCGGGTGGAAGAGGTGGCTGGCGCGGCTGCTCCACTTTGTGAAAGCTGAATGCGTGGAGTATGTGATGTAGCCAAC>1300
1201>GCCACTGGCTGGGGCCGTCCACCGCCGGGTGGAAGAGGTGGCTGGCGCGGCTGCTCCACTTTGTGAAAGCTGAATGCGTGGAGTATGTGATGTAGCCAAC>1300

              *         *         *         *         *         *         *         *         *         *
1301>GGAGGTGGGCGATGTGGGCCACCGGCTGGACTGGTTTTGGAGAATGTTGGATACGGTTTCAGAGTAGTGATTGTCCTCTTGGGTCAAGTCTTCTAATAGA>1400
1301>GGAGGTGGGCGATGTGGGCCACCGGCTGGACTGGTTTTGGAGAATGTTGGATACGGTTTCAGAGTAGTGATTGTCCTCTTGGGTCAAGTCTTCTAATAGA>1400
1301>GGAGGTGGGCGATGTGGGCCACCGGCTGGACTGGTTTTGGAGAATGTTGGATACGGTTTCAGAGTAGTGATTGTCCTCTTGGGTCAAGTCTTCTAATAGA>1400
1301>GGAGGTGGGCGATGTGGGCCACCGGCTGGACTGGTTTTGGAGAATGTTGGATACGGTTTCAGAGTAGTGATTGTCCTCTTGGGTCAAGTCTTCTAATAGA>1400
1301>GGAGGTGGGCGATGTGGGCCACCGGCTGGACTGGTTTTGGAGAATGTTGGATACGGTTTCAGAGTAGTGATTGTCCTCTTGGGTCAAGTCTTCTAATAGA>1400

              *         *         *         *         *         *         *         *         *         *
1401>TGAGGGGCTGCAAAATACACGCTCACATTTAAACGTGGAAATTATGTGCAAAATTAAGAGTTAATATTAATATAACTAAGTTATTAACACTTTCTTACTA>1500
1401>TGAGGGGCTGCAAAATACACGCTCACATTTAAACGTGGAAATTATGTGCAAAATTAAGAGTTAATATTAATATAACTAAGTTATTAACACTTTCTTACTA>1500
1401>TGAGGGGCTGCAAAATACACGCTCACATTTAAACGTGGAAATTATGTGCAAAATTAAGAGTTAATATTAATATAACTAAGTTATTAACACTTTCTTACTA>1500
1401>TGAGGGGCTGCAAAATACACGCTCACATTTAAACGTGGAAATTATGTGCAAAATTAAGAGTTAATATTAATATAACTAAGTTATTAACACTTTCTTACTA>1500
1401>TGAGGGGCTGCAAAATACACGCTCACATTTAAACGTGGAAATTATGTGCAAAATTAAGAGTTAATATTAATATAACTAAGTTATTAACACTTTCTTACTA>1500

              *         *         *         *         *         *         *         *         *         *
1501>GGATTATTTTGGTGCAAGTTTTTTAAATTTAATCCCTAAACTCTAAATTAGTTATTCAACCTATCAAAGTCTTCCTTACAATAGTGAGATGTGATTAAAT>1600
1501>GGATTATTTTGGTGCAAGTTTTTTAAATTTAATCCCTAAACTCTAAATTAGTTATTCAACCTATCAAAGTCTTCCTTACAATAGTGAGATGTGATTAAAT>1600
1501>GGATTATTTTGGTGCAAGTTTTTTAAATTTAATCCCTAAACTCTAAATTAGTTATTCAACCTATCAAAGTCTTCCTTACAATAGTGAGATGTGATTAAAT>1600
1501>GGATTATTTTGGTGCAAGTTTTTTAAATTTAATCCCTAAACTCTAAATTAGTTATTCAACCTATCAAAGTCTTCCTTACAATAGTGAGATGTGATTAAAT>1600
1501>GGATTATTTTGGTGCAAGTTTTTTAAATTGAATCCCTAAACTCTAAATTAGTTATTCAACCTATCAAAGTCTTCCTTACAATAGTGAGATGTGATTAAAT>1600

              *         *         *          *         *         *         *         *         *
1601>CATGATTCAACTGGCATAAATTAAAAAAAAA-TGGTAAAAATTATATAATATAAGAGTAAAAATAAATATAAAACTCGACTTCGCAGTGTGAATTTTGAA>1699
1601>CATGATTCAACTGGCATAAATTAAAAAAAAA-TGGTAAAAATTATATAATATAAGAGTAAAAATAAATATAAAACTCGACTTCGCAGTGTGAATTTTGAA>1699
1601>CATGATTCAACTGGCATAAATTAAAAAAAAA-TGGTAAAAATTATATAATATAAGAGTAAAAATAAATATAAAACTCGACTTCGCAGTGTGAATTTTGAA>1699
1601>CATGATTCAACTGGCATAAATTAAAAAAAAA-TGGTAAAAATTATATAATATAAGAGTAAAAATAAATATAAAACTCGACTTCGCAGTGTGAATTTTGAA>1699
1601>CATGATTCAACTGGCATAAATTAAAAAAAAAATGGTAAAAATTATATAATAT--GAGTAAAAATAAATATAAAACTCGACTTCGCAGTGTGAATTTTGAA>1698

      *         *         *         *         *         *         *         *         *         *
1700>TCATAAAGTTCTACTATAGTCAAAATAAAAGTAAGTTTCAAAATTCAGAATCGATTGTTATTTCTCTAAAAATAAAACATATGCTTTATAAGTTTCAAAT>1799
1700>TCATAAAGTTCTACTATAGTCAAAATAAAAGTAAGTTTCAAAATTCAGAATCGATTGTTATTTCTCTAAAAATAAAACATATGCTTTATAAGTTTCAAAT>1799
1700>TCATAAAGTTCTACTATAGTCAAAATAAAAGTAAGTTTCAAAATTCAGAATCGATTGTTATTTCTCTAAAAATAAAACATATGCTTTATAAGTTTCAAAT>1799
1700>TCATAAAGTTCTACTATAGTCAAAATAAAAGTAAGTTTCAAAATTCAGAATCGATTGTTATTTCTCTAAAAATAAAACATATGCTTTATAAGTTTCAAAT>1799
1699>TCATAAAGTTCTACTATAATCAAAATAAAAGTAAGTTTCAAAATTCAGAATCGATTGTTATTTCTCTAAAAATAAAACATATGCTTTATAAGTTTCAAAT>1798

      *         *         *         *         *         *         *         *         *         *
1800>CTCAAGTATCATATTGAGGTCTTATTTAAGGTATGAATATATGTCTCACATAATAAGTTAATATTTGTCTGAAGGTTAAAATGTAAAAATAGCACAAGTT>1899
1800>CTCAAGTATCATATTGAGGTCTTATTTAAGGTATGAATATATGTCTCACATAATAAGTTAATATTTGTCTGAAGGTTAAAATGTAAAAATAGCACAAGTT>1899
1800>CTCAAGTATCATATTGAGGTCTTATTTAAGGTATGAATATATGTCTCACATAATAAGTTAATATTTGTCTGAAGGTTAAAATGTAAAAATAGCACAAGTT>1899
1800>CTCAAGTATCATATTGAGGTCTTATTTAAGGTATGAATATATGTCTCACATAATAAGTTAATATTTGTCTGAAGGTTAAAATGTAAAAATAGCACAAGTT>1899
1799>CTCAAGTATCATATTGAGGTCTTATTTAAGGTATGAATATATGTCTCACATAATAAGTTAATATTTGTCTGAAGGTTAAAATGTAAAAATAGCACAAGTT>1898
```

```
        *         *         *         *         *         *         *         *         *         *
1900>AATATCAATATCCCAAAAAAA--AATCATTAAATAATAACTAATTTTAGAGACAAAAAACGTTAATCAATATATTAAATAATTTAAATATCATATTATAA>1997
1900>AATATCAATATCCCAAAAAAA--AATCATTAAATAATAACTAATTTTAGAGACAAAAAACGTTAATCAATATATTAAATAATTTAAATATCATATTATAA>1997
1900>AATATCAATATCCCAAAAAAAA-AATCATTAAATAATAACTAATTTTAGAGACAAAAAACGTTAATCAATATATTAAATAATTTAAATATCATATTATAA>1998
1900>AATATCAATATCCCAAAAAAAAAAATCATTAAATAATAACTAATTTTAGAGACAAAAAACGTTAATCAATATATTAAATAATTTAAATATCATATTATAA>1999
1899>AATATCAATATCCCAAAAAAAA-AATCATTAAATAATAATTAATTTTAGAGACAAAAAACGTTAATCAATATATTAAATAATTTAAATATCATATTATAA>1997

        *         *         *         *         *         *         *         *         *         *
1998>ATTAAAAATATTGATAGTTAAAATATTTTTTATTATAAATAAAAAATTATAATTATTTTGTGAATTAGAATATAAATTAATCTTTAATATTAGTTACCAA>2097
1998>ATTAAAAATATTGATAGTTAAAATATTTTTTATTATAAATAAAAAATTATAATTATTTTGTGAATTAGAATATAAATTAATCTTTAATATTAGTTACCAA>2097
1999>ATTAAAAATATTGATAGTTAAAATATTTTTTATTATAAATAAAAAATTATAATTATTTTGTGAATTAGAATATAAATTAATCTTTAATATTAGTTACCAA>2098
2000>ATTAAAAATATTGATAGTTAAAATATTTTTTATTATAAATAAAAAATTATAATTATTTTGTGAATTAGAATATAAATTAATCTTTAATATTAGTTACCAA>2099
1998>ATTAAAAATATTGATAGTTAAAATATTTTTTATTATAAATAAAAAATTATAATTATTTTGTGAATTAGAATATAAATTAATCTTTAATATTAGTTACCAA>2097

        *         *         *         *         *         *         *         *         *         *
2098>AATTTTAACTATGGAAATATTAATATTAATGTCTAAATTAGTTTATAATTTAAAATTAGATACCAAGTTAGAATCATGGATAGGAAATAGTTAAAATTTT>2197
2098>AATTTTAACTATGGAAATATTAATATTAATGTCTAAATTAGTTTATAATTTAAAATTAGATACCAAGTTAGAATCATGGATAGGAAATAGTTAAAATTTT>2197
2099>AATTTTAACTATGGAAATATTAATATTAATGTCTAAATTAGTTTATAATTTAAAATTAGATACCAAGTTAGAATCATGGATAGGAAATAGTTAAAATTTT>2198
2100>AATTTTAACTATGGAAATATTAATATTAATGTCTAAATTAGTTTATAATTTAAAATTAGATACCAAGTTAGAATCATGGATAGGAAATAGTTAAAATTTT>2199
2098>AATTTTAACTATGGAAATATTAATATTAATGTCTAAATTAGTTTATAATTTAAAATTAGATACCAAGTTAGAATCATGGATAGGAAATAGTTAAAATTTT>2197

        *         *         *         *         *         *         *         *         *         *
2198>AGTAACTTATTTAATAATTTTCTTGTATGGTAAAAGATATATACAATAATGCAAATCAAGACATTAAAAGACATTAAATGTGTACAAGTAAAATAATAAC>2297
2198>AGTAACTTATTTAATAATTTTCTTGTATGGTAAAAGATATATACAATAATGCAAATCAAGACATTAAAAGACATTAAATGTGTACAAGTAAAATAATAAC>2297
2199>AGTAACTTATTTAATAATTTTCTTGTATGGTAAAAGATATATACAATAATGCAAATCAAGACATTAAAAGACATTAAATGTGTACAAGTAAAATAATAAC>2298
2200>AGTAACTTATTTAATAATTTTCTTGTATGGTAAAAGATATATACAATAATGCAAATCAAGACATTAAAAGACATTAAATGTGTACAAGTAAAATAATAAC>2299
2198>AGTAACTTATTTAATAATTTTCTTGTATGGTAAAAGATATATACAATAATGCAAATCAAG----------------------------TAAAATAATAAC>2269

        *         *         *         *         *         *         *         *         *         *
2298>AAACAGTTTTTTGAAATTACCTCAGATATTTATCTTCTTAAGTTAAAATAAAATAATTTTAGAATAATTTTTATTAACCCATTTCAATAAGAAAAAATTA>2397
2298>AAACAGTTTTTTGAAATTACCTCAGATATTTATCTTCTTAAGTTAAAATAAAATAATTTTAGAATAATTTTTATTAACCCATTTCAATAAGAAAAAATTA>2397
2299>AAACAGTTTTTTGAAATTACCTCAGATATTTATCTTCTTAAGTTAAAATAAAATAATTTTAGAATAATTTTTATTAACCCATTTCAATAAGAAAAAATTA>2398
2300>AAACAGTTTTTTGAAATTACCTCAGATATTTATCTTCTTAAGTTAAAATAAAATCATTTTAGAATAATTTTTATTAACCCATTTCAATAAGAAAAAATTA>2399
2270>AAACAGTTTTTTGAAATTACCTCAGATATTTATCTTCTTAAGTTAAAATAAAATAATTTTAGAATAATTTTTATTAACCCATTTCAATAAGAAAAAATTA>2369

        *         *         *         *         *         *                        *         *         *
2398>GTCTAGTTTTTTATTCTTTTAAATCCATACCGTATAAAATAAGAGTTTGAGTGTGT--------------GTATATATATATATATATATATATATATAT>2483
2398>GTCTAGTTTTTTATTCTTTTAAATCCATACCGTATAAAATAAGAGTTTGAGTGTGTATATAtatatatatatatatatatatatatatatatatatatatat>2497
2399>GTCTAGTTTTTTATTCTTTTAAATCCATACCGTATAAAATAAGAGTTTGAGTGTGT--------------GTATATATATATATATATATATAtatatat>2484
2400>GTCTAGTTTTTTATTCTTTTAAATCCATACCGTATAAAATAAGAGTTTGAGTGTGT----------------------------------------ATAT>2459
2370>GTCTAGTTTTTTATTCTTTTAAATCCATACCGTATA[redacted]--------------[redacted]>2405

            *         *         *         *         *         *         *         *         *         *
2484>ATATATATATATATATATATATATATATATATATATATCATTTTATATGTTAAGAGTGAAGAGTGGTAAGTGACAAAAACGATATAAATATATTATGAAT>2583
2498>atatatatatatatatatatatatatatATATATATATCATTTTATATGTTAAGAGTGAAGAGTGGTAAGTGACAAAAACGATATAAATATATTATGAAT>2597
2485>aTATATATATATATATATATATATATATATATA----TCATTTTATATGTTAAGAGTGAAGAGTGGTAAGTGACAAAAACGATATAAATATATTATGAAT>2580
2460>ATATATATATATATATATATAtatatatataTATATATCATTTTATATGTTAAGAGTGAAGAGTGGTAAGTGACAAAAACGATATAAATATATTATGAAT>2559
2405>---------------------------------------------------------------------------------------------------->2405

            *         *         *         *         *         *         *         *         *         *
2584>AAAATGATTTTATATTAGTTTTAAATCATGTAATGAAAAAATAATGGTAATTGTTATACACAAAAATAATTTAAATATATTATAATTGATAATTCTATAA>2683
2598>AAAATGATTTTATATTAGTTTTAAATCATGTAATGAAAAAATAATGGTAATTGTTATACACAAAAATAATTTAAATATATTATAATTGATAATTCTATAA>2697
2581>AAAATGATTTTATATTAGTTTTAAATCATGTAATGAAAAAATAATGGTAATTGTTATACACAAAAATAATTTAAATATATTATAATTGATAATTCTATAA>2680
2560>AAAATGATTTTATATTAGTTTTAAATCATGTAATGAAAAAATAATGGTAATTGTTATACACAAAAATAATTTAAATATATTATAATTGATAATTCTATAA>2659
2405>---------------------------------------------------------------------------------------------------->2405

            *         *         *         *         *         *         *         *         *         *
2684>ATATAAATCATATTCATCACTTTTATTTTTAGAGATAAATTCATGTTATGAGACTAGAAAATAAATGATTTTACAATCTTTCAAAATTGTAAATTATCAA>2783
2698>ATATAAATCATATTCATCACTTTTATTTTTAGAGATAAATTCATGTTATGAGACTAGAAAATAAATGATTTTACAATCTTTCAAAATTGTAAATTATCAA>2797
2681>ATATAAATCATATTCATCACTTTTATTTTTAGAGATAAATTCATGTTATGAGACTAGAAAATAAATGATTTTACAATCTTTCAAAATTGTAAATTATCAA>2780
2660>ATATAAATCATATTCATCACTTTTATTTTTAGAGATAAATTCATGTTATGAGACTAGAAAATAAATGATTTTACAATCTTTCAAAATTGTAAATTATCAA>2759
2405>---------------------------------------------------------------------------------------------------->2405

            *         *         *         *         *         *         *         *         *         *
2784>TTGAAAAAGTTTTAGAAACGTCTAAGTTGTATATTATTTGTAATGAAAATGTAATGTTAAAATTAAATTATTGAAAAATATAAAAAATTATTATTTTATT>2883
2798>TTGAAAAAGTTTTAGAAACGTCTAAGTTGTATATTATTTGTAATGAAAATGTAATGTTAAAATTAAATTATTGAAAAATATAAAAAATTATTATTTTATT>2897
2781>TTGAAAAAGTTTTAGAAACGTCTAAGTTGTATATTATTTGTAATGAAAATGTAATGTTAAAATTAAATTATTGAAAAATATAAAAAATTATTATTTTATT>2880
2760>TTGAAAAAGTTTTAGAAACGTCTAAGTTGTATATTATTTGTAATGAAAATGTAATGTTAAAATTAAATTATTGAAAAATATAAAAAATTATTATTTTATT>2859
2405>---------------------------------------------------------------------------------------------------->2405
```

```
            *         *         *         *         *         *         *         *         *         *
2884>TATCTTAATAGAGAATATATTTTTATTTTAATTAAATGAGTCTGAAAATATACATATATTCATATTAATTTAAAATATTATATTAAAATAATCTTGATTT>2983
2898>TATCTTAATAGAGAATATATTTTTATTTTAATTAAATGAGTCTGAAAATATACATATATTCATATTAATTTAAAATATTATATTAAAATAATCTTGATTT>2997
2881>TATCTTAATAGAGAATATATTTTTATTTTAATTAAATGAGTCTGAAAATATACATATATTCATATTAATTTAAAATATTATATTAAAATAATCTTGATTT>2980
2860>TATCTTAATAGAGAATATATTTTTATTTTAATTAAATGAGTCTGAAAATATACATATATTCATATTAATTTAAAATATTATATTAAAATAATCTTGATTT>2959
2405>----------------------------------------------------------------------------------------------------->2405

            *         *         *         *         *         *         *         *         *         *
2984>TTAGGAAAGTATATAACGCGACAATTAGAAATCATTAGATATTTAATAAGCAAATACTTTCATAAGAACTAAAAAAAATCATATTAAAATTAAAATATAAA>3083
2998>TTAGGAAAGTATATAACGCGACAATTAGAAATCATTAGATATTTAATAAGCAAATACTTTCATAAGAACTAAAAAAAATCATATTAAAATTAAAATATAAA>3097
2981>TTAGGAAAGTATATAACGCGACAATTAGAAATCATTAGATATTTAATAAGCAAATACTTTCATAAGAACTAAAAAAAATCATATTAAAATTAAAATATAAA>3080
2960>TTAGGAAAGTATATAACGCGACAATTAGAAATCATTAGATATTTAATAAGCAAATACTTTCATAAGAACTAAAAAAAATCATATTAAAATTAAAATATAAA>3059
2405>----------------------------------------------------------------------------------------------------->2405

            *         *         *         *         *         *         *         *         *         *
3084>AATAATAAAAAGAGAAATAACAAATTATTAATAAGAATAAAGATGATGGATATTTTGAATGATAAGAAGGATAGAGATGTGATTTTAAAGTGCTTAGCAT>3183
3098>AATAATAAAAAGAGAAATAACAAATTATTAATAAGAATAAAGATGATGGATATTTTGAATGATAAGAAGGATAGAGATGTGATTTTAAAGTGCTTAGCAT>3197
3081>AATAATAAAAAGAGAAATAACAAATTATTAATAAGAATAAAGATGATGGATATTTTGAATGATAAGAAGGATAGAGATGTGATTTTAAAGTGCTTAGCAT>3180
3060>AATAATAAAAAGAGAAATAACAAATTATTAATAAGAATAAAGATGATGGATATTTTGAATGATAAGAAGGATAGAGATGTGATTTTAAAGTGCTTAGCAT>3159
2405>----------------------------------------------------------------------------------------------------->2405

            *         *         *         *         *         *         *         *         *         *
3184>GAATGTAAAAGTTGAAGAATAAATAAAATTGAGGTTTATATTAAAAACTGCCATCTACAAAAATCATCGATAAAATGGTAAAGAAAAACAAATCAGTAAA>3283
3198>GAATGTAAAAGTTGAAGAATAAATAAAATTGAGGTTTATATTAAAAACTGCCATCTACAAAAATCATCGATAAAATGGTAAAGAAAAACAAATCAGTAAA>3297
3181>GAATGTAAAAGTTGAAGAATAAATAAAATTGAGGTTTATATTAAAAACTGCCATCTACAAAAATCATCGATAAAATGGTAAAGAAAAACAAATCAGTAAA>3280
3160>GAATGTAAAAGTTGAAGAATAAATAAAATTGAGGTTTATATTAAAAACTGCCATCTACAAAAATCATCGATAAAATGGTAAAGAAAAACAAATCAGTAAA>3259
2405>----------------------------------------------------------------------------------------------------->2405

            *         *         *         *         *         *         *         *         *
3284>TAAAAAAGTGGTTGAAAAAAAATTATATTTAAATTCAGGTATTATTTTTTCAACTATCTTACTTTTTCATATAACAATATTAAAAAAAAAA---------->3373
3298>TAAAAAAGTGGTTGAAAAAAAATTATATTTAAATTCAGGTATTATTTTTTCAACTATCTTACTTTTTCATATAACAATATTAAAAAAAAAAATATA----->3392
3281>TAAAAAAGTGGTTGAAAAAAAATTATATTTAAATTCAGGTATTATTTTTTCAACTATCTTACTTTTTCATATAACAATATTAAAAAAAAAAAAAAAA---->3376
3260>TAAAAAAGTGGTTGAAAAAAAATTATATTTAAATTCAGGTATTATTTTTTCAACTATCTTACTTTTTCATATAACAATATTAAAAAAAAAAAAAaaaatat>3359
2405>----------------------------------------------------------------------------------------------------->2405

                              *         *         *         *         *         *         *         *
3374>-----------------TATATATATATATATATAACCTATAAATTTATTATTTTTATTATTTCTCTAAGCGTATATTAGATGTTAATACACCTTTTTTA>3456
3393>-----------------tatatatataTATATATAACCTATAAATTTATTATTTTTATTATTTCTCTAAGCGTATATTAGATGTTAATACACCTTTTTTA>3475
3377>-----------------TATATATATATATATATAACCTATAAATTTATTATTTTTATTATTTCTCTAAGCGTATATTAGATGTTAATACACCTTTTTTA>3459
3360>ataTATATATATATATATATATATATATATATATAACCTATAAATTTATTATTTTTATTATTTCTCTAAGCGTATATTAGATGTTAATACACCTTTTTTA>3459
2405>----------------------------------------------------------------------------------------------------->2405

         *         *         *         *         *         *         *         *         *         *
3457>GTGTCAATGAATGATTTTTCTTATATAAATTATATTTTTATTTTACTTTATTTATAATATTATATTGAATTTGAGAAGGAAAAAAAATGAACTTTAACAA>3556
3476>GTGTCAATGAATGATTTTTCTTATATAAATTATATTTTTATTTTACTTTATTTATAATATTATATTGAATTTGAGAAGGAAAAAAAATGAACTTTAACAA>3575
3460>GTGTCAATGAATGATTTTTCTTATATAAATTATATTTTTATTTTACTTTATTTATAATATTATATTGAATTTGAGAAGGAAAAAAAATGAACTTTAACAA>3559
3460>GTGTCAATGAATGATTTTTCTTATATAAATTATATTTTTATTTTACTTTATTTATAATATTATATTGAATTTGAGAAGGAAAAAAAATGAACTTTAACAA>3559
2405>----------------------------------------------------------------------------------------------------->2405

         *         *         *         *         *          *         *         *         *         *
3557>GATATAAGTGTAATTGTACAGGATGAAGCTCACTTTAAATTAAA-AAAAAAATTTAAATACGTATAGTCAAAATTAATACAAATATTAAATTTCAACTGA>3655
3576>GATATAAGTGTAATTGTACAGGATGAAGCTCACTTTAAATTAAA-AAAAAAATTTAAATACGTATAGTCAAAATTAATACAAATATTAAATTTCAACTGA>3674
3560>GATATAAGTGTAATTGTACAGGATGAAGCTCACTTTAAATTAAA-AAAAAAATTTAAATACGTATAGTCAAAATTAATACAAATATTAAATTTCAACTGA>3658
3560>GATATAAGTGTAATTGTACAGGATGAAGCTCACTTTAAATTAAAAAAAAAAATTTAAATACGTATAGTCAAAATTAATACAAATATTAAATTTCAACTGA>3659
2405>----------------------------------------------------------------------------------------------------->2405

          *         *         *         *         *         *         *         *         *         *
3656>TTAGAAAGAATATAGTGTGAGAAGATGTGGACATGTCTTAGCACATATGTTATTTAATAAATTTTAATTCTACTAATTTATCGTACTCATTGATATCTTA>3755
3675>TTAGAAAGAATATAGTGTGAGAAGATGTGGACATGTCTTAGCACATATGTTATTTAATAAATTTTAATTCTACTAATTTATCGTACTCATTGATATCTTA>3774
3659>TTAGAAAGAATATAGTGTGAGAAGATGTGGACATGTCTTAGCACATATGTTATTTAATAAATTTTAATTCTACTAATTTATCGTACTCATTGATATCTTA>3758
3660>TTAGAAAGAATATAGTGTGAGAAGATGTGGACATGTCTTAGCACATATGTTATTTAATAAATTTTAATTCTACTAATTTATCGTACTCATTGATATCTTA>3759
2405>----------------------------------------------------------------------------------------------------->2405

          *         *         *         *         *         *         *         *         *         *
3756>ATATTAAAGTAGATAATATTTGTTGTCCATTTATGTTCACTATCGTGAAGATGCATACATAACTAAGTCTAATAAAAGTTTATTTAAAATTCAAGGACTT>3855
3775>ATATTAAAGTAGATAATATTTGTTGTCCATTTATGTTCACTATCGTGAAGATGCATACATAACTAAGTCTAATAAAAGTTTATTTAAAATTCAAGGACTT>3874
3759>ATATTAAAGTAGATAATATTTGTTGTCCATTTATGTTCACTATCGTGAAGATGCATACATAACTAAGTCTAATAAAAGTTTATTTAAAATTCAAGGACTT>3858
3760>ATATTAAAGTAGATAATATTTGTTGTCCATTTATGTTCACTATCGTGAAGATGCATACATAACTAAGTCTAATAAAAGTTTATTTAAAATTCAAGGACTT>3859
2405>----------------------------------------------------------------------------------------------------->2405
```

```
          *         *         *         *         *         *         *         *         *         *
3856>ATAATTTGTTTGTTGTGAAAAGTACATTTTCTTTTCATTTCAGTCTGAGATTTTGGAGTCTTTTGAAACAGTGACAGGCTAAAAACTTAGATATATCATA>3955
3875>ATAATTTGTTTGTTGTGAAAAGTACATTTTCTTTTCATTTCAGTCTGAGATTTTGGAGTCTTTTGAAACAGTGACAGGCTAAAAACTTAGATATATCATA>3974
3859>ATAATTTGTTTGTTGTGAAAAGTACATTTTCTTTTCATTTCAGTCTGAGATTTTGGAGTCTTTTGAAACAGTGACAGGCTAAAAACTTAGATATATCATA>3958
3860>ATAATTTGTTTGTTGTGAAAAGTACATTTTCTTTTCATTTCAGTCTGAGATTTTGGAGTCTTTTGAAACAGTGACAGGCTAAAAACTTAGATATATCATA>3959
2405>---------------------------------------------------------------------------------------------------->2405

          *         *         *         *         *         *         *         *         *         *
3956>TATGTTGGGTTTTCTACCTAAAACAAGGGGAATAAGAAAGAATATTCTACACATTCTGACTTATTTTGATGTGTTCCTGTTGACATTAACATCTATAATG>4055
3975>TATGTTGGGTTTTCTACCTAAAACAAGGGGAATAAGAAAGAATATTCTACACATTCTGACTTATTTTGATGTGTTCCTGTTGACATTAACATCTATAATG>4074
3959>TATGTTGGGTTTTCTACCTAAAACAAGGGGAATAAGAAAGAATATTCTACACATTCTGACTTATTTTGATGTGTTCCTGTTGACATTAACATCTATAATG>4058
3960>TATGTTGGGTTTTCTACCTAAAACAAGGGGAATAAGAAAGAATATTCTACACATTCTGACTTATTTTGATGTGTTCCTGTTGACATTAACATCTATAATG>4059
2405>---------------------------------------------------------------------------------------------------->2405

          *         *         *         *         *         *         *         *         *         *
4056>TTTTGGATTTATCTTTCAAAACATGTCCAATGCTGTGGTAGTTGATTAAGTTTTGGAGATTTTTCATCCAAACAATGCTTATCCTTAAAATAAAACGTCT>4155
4075>TTTTGGATTTATCTTTCAAAACATGTCCAATGCTGTGGTAGTTGATTAAGTTTTGGAGATTTTTCATCCAAACAATGCTTATCCTTAAAATAAAACGTCT>4174
4059>TTTTGGATTTATCTTTCAAAACATGTCCAATGCTGTGGTAGTTGATTAAGTTTTGGAGATTTTTCATCCAAACAATGCTTATCCTTAAAATAAAACGTCT>4158
4060>TTTTGGATTTATCTTTCAAAACATGTCCAATGCTGTGGTAGTTGATTAAGTTTTGGAGATTTTTCATCCAAACAATGCTTATCCTTAAAATAAAACGTCT>4159
2405>---------------------------------------------------------------------------------------------------->2405

          *         *         *         *         *         *         *         *         *         *
4156>AATCATTAAACAGTGCTCCTCTTTGCTTGCAATCACTTTATGATTGGTGTTGGGTTGAGAAGAAATTAAAAGAATAGAAAAGGATGAAATTAGTTTTTGT>4255
4175>AATCATTAAACAGTGCTCCTCTTTGCTTGCAATCACTTTATGATTGGTGTTGGGTTGAGAAGAAATTAAAAGAATAGAAAAGGATGAAATTAGTTTTTGT>4274
4159>AATCATTAAACAGTGCTCCTCTTTGCTTGCAATCACTTTATGATTGGTGTTGGGTTGAGAAGAAATTAAAAGAATAGAAAAGGATGAAATTAGTTTTTGT>4258
4160>AATCATTAAACAGTGCTCCTCTTTGCTTGCAATCACTTTATGATTGGTGTTGGGTTGAGAAGAAATTAAAAGAATAGAAAAGGATGAAATTAGTTTTTGT>4259
2405>---------------------------------------------------------------------------------------------------->2405

          *         *         *         *         *         *         *           *         *         *
4256>TACTTGGTGACACAGAATGAAAAAATAATTTCAGAAATCATAAAATTTACTGAGTTTTTTTTTTTT--TTTGGAGATTCTATTCATTTAATCTCTATTTAA>4353
4275>TACTTGGTGACACAGAATGAAAAAATAATTTCAGAAATCATAAAATTTACTGAGTTTTTTTTTTTT--TTTGGAGATTCTATTCATTTAATctctatttaa>4372
4259>TACTTGGTGACACAGAATGAAAAAATAATTTCAGAAATCATAAAATTTACTGAGTTTTTTTTTTTTTTTTTGGAGATTCTATTCATTTAATCTCTATTTAA>4358
4260>TACTTGGTGACACAGAATGAAAAAATAATTTCAGAAATCATAAAATTTACTGAGTTTTTTTTTTTT--TTT------------------------------>4327
2405>---------------------------------------------------------------------------------------------------->2405

            *         *         *         *         *         *         *         *         *         *
4354>ATTTTTTATTTACTTTTTTGATTTTATTTTATTTCATCTCTTCAATAAAATACTTTTAAATGTATTTGTTTCATATTTTATTTTCTAATCGTTCATTTAA>4453
4373>aTTTTTTATTTACTTTTTTGATTTTATTTTATTTCATCTCTTCAATAAAATACTTTTAAATGTATTTGTTTCATATTTTATTTTCTAATCGTTCATTTAA>4472
4359>ATTTTTTATTTACTTttttgattttaTTTTATTTCATCTCTTCAATAAAATACTTTTAAATGTATTTGTTTCATATTTTATTTTCTAATCGTTCATTTAA>4458
4328>-----------------TTG-------------------------------------------------------------------------------->4330
2405>---------------------------------------------------------------------------------------------------->2405

            *         *         *         *         *         *         *         *         *         *
4454>TTTTCACTAATAATTTTTCTCTATCGGTAACTGATTTTATATTAAATTAAGACTCCTAAAAATCTCAAACCATTATTATTTCGCAAGTAGTAGTACTTAG>4553
4473>TTTTCACTAATAATTTTTCTCTATCGGTAACTGATTTTATATTAAATTAAGACTCCTAAAAATCTCAAACCATTATTATTTCGCAAGTAGTAGTACTTAG>4572
4459>TTTTCACTAATAATTTTTCTCTATCGGTAACTGATTTTATATTAAATTAAGACTCCTAAAAATCTCAAACCATTATTATTTCGCAAGTAGTAGTACTTAG>4558
4330>---------------------------------------------------------------------------------------------------->4330
2405>---------------------------------------------------------------------------------------------------->2405

            *         *         *         *         *                     *         *         *         *
4554>GTCTCTGTTTTCTTTTCCTGTCTTTTTAAGAACGAAATATACACATCT-----------TTATATATATATATATATATATATATATATATATATATATA>4642
4573>GTCTCTGTTTTCTTTTCCTGTCTTTTTAAGAACGAAATATACACATCT-----------TTNNNNNNNNNNNNNNNNNNNNNNNNNNNNNNNNNNNNN>4661
4559>GTCTCTGTTTTCTTTTCCTGTCTTTTTAAGAACGAAATATACACATCTNNNNNNNNNNNNNNNNNNNNNNNNNNNNNNNNNNNNNNNNNNNNNNNNNN>4658
4330>---------------------------------------------------------------------------------------------------->4330
2406>-----------------------------------GAATAAGAGTTTGAG-----------TGNNNNNNNNNNNNNNNNNNNNNNNNNNNNNNNNNNNNNNN>2461

             *         *         *         *         *         *         *         *         *         *
4643>TATATGTATATATATATATATATGTATATATATATATATGTATATATATATATATGTATATATATAATCTATCTTCATAGTAATAATAATAAAAATAATA>4742
4662>NNNNNNNNNNNNNNNNNNNNNNNNNNNNNNNNNNNNNNNNNNNNNNNNNNNNNNNNNNNN---------------------------------------->4721
4659>NNNNNNNNNNNNNNNNNNNNNNNNNNNNNNNNNNNNNNNNNNNNNNNNTGTATATATATA-ATCTAATCTATCTTCATAGTAATAATAATAAAAATAATA>4757
4331>---------NNNNNNNNNNNNNNNNNNNNNNNNNNNNNNNNNNNNNNNNNNNNNNNNNNNNNNNNNNNNNNNNNNNNNNNNNNNNNNNNNNNNNNNNNNNNN>4421
2462>NNNNNNNNNNNNNNNNNNNNNNNNNNNNNNNNNNNNNNNNNNNNNNNNNNNNNNNNNNNNNATATAATCTATCTTCATAGTAATAATAATAAAAATAATA>2561

             *         *                  *         *           *         *         *         *         *
4743>ATAACCATCAAATGAAAA--------TTATTAATTTACTTTTTATAATTATCAA-TATACTACCTTATTTTCAATATTTTTTATATTATTACATTCATTA>4833
4722>NTAACCATCAAATGAAAA--------TTATTAATTTACTTTTTATAATTATCAA-TATACTACCTTATTTTCAATATTTTTTATATTATTACATTCATTA>4812
4758>ATAACCATCAAATGAAAA--------TTATTAATTTACTTTTTATAATTATCAA-TATACTACCTTATTTTCAATATTTTTTATATTATTACATTCATTA>4848
4422>NNNNNNNNNAAATGAAAA--------TTATTAATTTACTTTTTATAATTATCAA-TATACTACCTTATTTTCAATATTTTTTATATTATTACATTCATTA>4512
2562>ATAACCATCAAATGAAAAGTTACTTTTTATTAATTTACTTTTTATAATTATCAAATATACTACCTTATTTTCAATATTTTTTATATTATTACATTCATTA>2661
```

```
           *         *         *         *         *         *         *         *         *         *
4834>TTATTATTATTATCATTAATGTTGATGTCTTGGTTATCATTATTATGAACATCTTTTATTGTTATACTTTGTACACCAGGCATTTAATATAATTAATTCT>4933
4813>TTATTATTATTATCATTAATGTTGATGTCTTGGTTATCATTATTATGAACATCTTTTATTGTTATACTTTGTACACCAGGCATTTAATATAATTAATTCT>4912
4849>TTATTATTATTATCATTAATGTTGATGTCTTGGTTATCATTATTATGAACATCTTTTATTGTTATACTTTGTACACCAGGCATTTAATATAATTAATTCT>4948
4513>TTATTATTATTATCATTAATGTTGATGTCTTGGTTATCATTATTATGAACATCTTTTATTGTTATACTTTGTACACCAGGCATTTAATATAATTAATTCT>4612
2662>TTATTATTATTATCATTAATGTTGATGTCTTGGTTATCATTATTATGAACATCTTTTATTGTTATACTTTGTACACCAGGCATTTAATATAATTAATTCT>2761

           *         *         *         *         *         *         *         *         *         *
4934>TATTGTATTTTGATTAGATTATATTACTTATATATCGTACTGATATTAACTAGTCATAAAATAATGTAAACGAGAGATATAGTAAAAAAAATAATGATAA>5033
4913>TATTGTATTTTGATTAGATTATATTACTTATATATCGTACTGATATTAACTAGTCATAAAATAATGTAAACGAGAGATATAGTAAAAAAAATAATGATAA>5012
4949>TATTGTATTTTGATTAGATTATATTACTTATATATCGTACTGATATTAACTAGTCATAAAATAATGTAAACGAGAGATATAGTAAAAAAAATAATGATAA>5048
4613>TATTGTATTTTGATTAGATTATATTACTTATATATCGTACTGATATTAACTAGTCATAAAATAATGTAAACGAGAGATATAGTAAAAAAAATAATGATAA>4712
2762>TATTGTATTTTGATTAGATTATATTACTTATATATCGTACTGATATTAACTAGTCATAAAATAATGTAAACGAGAGATATAGTAAAAAAAATAATGATAA>2861

           *         *         *         *         *         *         *         *
5034>TTATAATGGTTGATTAATAATAAGAAATTGTACTAAAAATATTTTTGTTTTCTTTTTCCGAGCATTATAAACTTTCGCTTTT------------------>5115
5013>TTATAATGGTTGATTAATAATAAGAAATTGTACTAAAAATATTTTTGTTTTCTTTTTCCGAGCATTATAAACTTTCGCTTTT------------------>5094
5049>TTATAATGGTTGATTAATAATAAGAAATTGTACTAAAAATATTTTTGTTTTCTTTTTCCGAGCATTATAAACTTTCGCTTTT------------------>5130
4713>TTATAATGGTTGATTAATAATAAGAAATTGTACTAAAAATATTTTTGTTTTCTTTTTCCGAGCATTATAAACTTTCGCTTTT------------------>4794
2862>TTATAATGGTTGATTAATAATAAGAAATTGTACTAATAATATTTTTGTCTTCTTTTTCCGAGCATTATAAACTTTCGCTTTTCTTTCGGTTGAGCATTAT>2961

                       *         *         *         *         *         *         *         *         *
5116>--------------CTTTCGGTTGTCACAGTCATATTTTCGTTTTGTAGTGTGTACCTGATAGACAAGGGTCTTTATTATGATATATTTGCATGAAGATC>5201
5095>--------------CTTTCGGTTGTCACAGTCATATTTTCGTTTTGTAGTGTGTACCTGATAGACAAGGGTCTTTATTATGATATATTTGCATGAAGATC>5180
5131>--------------CTTTCGGTTGTCACAGTCATATTTTCGTTTTGTAGTGTGTACCTGATAGACAAGGGTCTTTATTATGATATATTTGCATGAAGATC>5216
4795>--------------CTTTCGGTTGTCACAGTCATATTTTCGTTTTGTAGTGTGTACCTGATAGACAAGGGTCTTTATTATGATATATTTGCATGAAGATC>4880
2962>AAACTTTCGCTTTTCTTTCGGTTGTCACAGTCATATTTTCGTTTTGTAGTGTGTACCTGATAGACAAGGGTCTTTATTATGATATATTTGCATGAAGATC>3061

             *         *         *         *         *         *         *         *         *         *
5202>GTGGTGTTTGTTTACGTACATATATAGTGTGGGATCACTATACTTTCATGCCTCTGCATTAAAACCAATTTCGTGGTTTAGAATTTTGCTAAGGACCCTC>5301
5181>GTGGTGTTTGTTTACGTACATATATAGTGTGGGATCACTATACTTTCATGCCTCTGCATTAAAACCAATTTCGTGGTTTAGAATTTTGCTAAGGACCCTC>5280
5217>GTGGTGTTTGTTTACGTACATATATAGTGTGGGATCACTATACTTTCATGCCTCTGCATTAAAACCAATTTCGTGGTTTAGAATTTTGCTAAGGACCCTC>5316
4881>GTGGTGTTTGTTTACGTACATATATAGTGTGGGATCACTATACTTTCATGCCTCTGCATTAAAACCAATTTCGTGGTTTAGAATTTTGCTAAGGACCCTC>4980
3062>GTGGTGTTTGTTTACGTACATATATAGCGTGGGATCACTATACTTTCATGCCTCTGCATTAAAACCAATTTCGTGGTTTAGACTTTTGCTAAGGACCCTC>3161

             *         *         *         *         *         *         *         *         *         *
5302>ACCAAACGCTCGTCTCAGCCAATCTATTAGGATAAGGACCACATGCCCTAGCTCTAGATTAGTCTTCATATGCCACACGCATTTAATCAATCTTCACTAA>5401
5281>ACCAAACGCTCGTCTCAGCCAATCTATTAGGATAAGGACCACATGCCCTAGCTCTAGATTAGTCTTCATATGCCACACGCATTTAATCAATCTTCACTAA>5380
5317>ACCAAACGCTCGTCTCAGCCAATCTATTAGGATAAGGACCACATGCCCTAGCTCTAGATTAGTCTTCATATGCCACACGCATTTAATCAATCTTCACTAA>5416
4981>ACCAAACGCTCGTCTCAGCCAATCTATTAGGATAAGGACCACATGCCCTAGCTCTAGATTAGTCTTCATATGCCACACGCATTTAATCAATCTTCACTAA>5080
3162>ACCAAACGCTCGTCTCAGCCAATCTATTAGGATAAGGACCACATGCCCTAGCTCTAGATTAGTCTTCATATGCCACACGCATTTAATCAATCTTCACTAA>3261

             *                 *         *         *         *         *         *         *         *
5402>TTAATTAATTAATT--------AATTCTTCTCAGTGACCTACCTAACGTGGTCCATGTATTTTAGGGGTTGAACGTTTGGTGAGATGCAGTGGCAGTTGT>5493
5381>TTAATTAATTAATT--------AATTCTTCTCAGTGACCTACCTAACGTGGTCCATGTATTTTAGGGGTTGAACGTTTGGTGAGATGCAGTGGCAGTTGT>5472
5417>TTAATTAATTAATT--------AATTCTTCTCAGTGACCTACCTAACGTGGTCCATGTATTTTAGGGGTTGAACGTTTGGTGAGATGCAGTGGCAGTTGT>5508
5081>TTAATTAATTAATT--------AATTCTTCTCAGTGACCTACCTAACGTGGTCCATGTATTTTAGGGGTTGAACGTTTGGTGAGATGCAGTGGCAGTTGT>5172
3262>TTAATTAATTAATTAATTAATTAATTCTTCTCAGTGACCTACCTAACGTGGTCCATGTATTTTAGGGGTTGAACGTTTGGTGAGATGCAGTGGCAGTTGT>3361

           *         *         *         *         *         *         *         *         *         *
5494>AATGTTATACGAAACCTGTTTTCAGTTTGTCATTCATATGTATGTATGCATGGATGTCTATTTCTGCATAACTAAATAACTACCGGTTTACCTGAAGCTG>5593
5473>AATGTTATACGAAACCTGTTTTCAGTTTGTCATTCATATGTATGTATGCATGGATGTCTATTTCTGCATAACTAAATAACTACCGGTTTACCTGAAGCTG>5572
5509>AATGTTATACGAAACCTGTTTTCAGTTTGTCATTCATATGTATGTATGCATGGATGTCTATTTCTGCATAACTAAATAACTACCGGTTTACCTGAAGCTG>5608
5173>AATGTTATACGAAACCTGTTTTCAGTTTGTCATTCATATGTATGTATGCATGGATGTCTATTTCTGCATAACTAAATAACTACCGGTTTACCTGAAGCTG>5272
3362>AATGTTATACGAAACCTGTTTTCAGTTTGTCATTCATATGTATGTATGCATGGATGTCTATTTCTGCATAACTAAATAACTACCGGTTTACCTGAAGCTG>3461

           *         *         *         *         *         *         *         *         *         *
5594>GTAGTTGAACTTGTAATGGCTCTTCACTCGGGACCCATCTCAGGCTCGACTCAGCCTGCCCCGCCGCGTTTCCTCCTTGACTCACCGCCAGCAAGTGGAA>5693
5573>GTAGTTGAACTTGTAATGGCTCTTCACTCGGGACCCATCTCAGGCTCGACTCAGCCTGCCCCGCCGCGTTTCCTCCTTGACTCACCGCCAGCAAGTGGAA>5672
5609>GTAGTTGAACTTGTAATGGCTCTTCACTCGGGACCCATCTCAGGCTCGACTCAGCCTGCCCCGCCGCGTTTCCTCCTTGACTCACCGCCAGCAAGTGGAA>5708
5273>GTAGTTGAACTTGTAATGGCTCTTCACTCGGGACCCATCTCAGGCTCGACTCAGCCTGCCCCGCCGCGTTTCCTCCTTGACTCACCGCCAGCAAGTGGAA>5372
3462>GTAGTTGAACTTGTAATGGCTCTTCACTCGGGACCCATCTCAGGCTCGACTCAGCCTGCCCCGCCGCATTTCCTCCTTGACTCACCGCCAGCAAGTGGAA>3561

           *         *         *         *         *         *         *         *         *         *
5694>GTCCGAGTCCAAGTTGTTCGACCCGTCGTTCGGAGACCCGACCCGGATATCCTCCGGCATCTCCAGCTGCATCAGCTCGCTGGGCTCCGCCCTCGTCACG>5793
5673>GTCCGAGTCCAAGTTGTTCGACCCGTCGTTCGGAGACCCGACCCGGATATCCTCCGGCATCTCCAGCTGCATCAGCTCGCTGGGCTCCGCCCTCGTCACG>5772
5709>GTCCGAGTCCAAGTTGTTCGACCCGTCGTTCGGAGACCCGACCCGGATATCCTCCGGCATCTCCAGCTGCATCAGCTCGCTGGGCTCCGCCCTCGTCACG>5808
5373>GTCCGAGTCCAAGTTGTTCGACCCGTCGTTCGGAGACCCGACCCGGATATCCTCCGGCATCTCCAGCTGCATCAGCTCGCTGGGCTCCGCCCTCGTCACG>5472
3562>GTCCGAGTCCAAGTTGTTCGACCCGTCGTTCGGAGACCCGACCCGGATATCCTCCGGCATCTCCAGCTGCATCAGCTCGCTGGGCTCCGCCCTCGTCACG>3661
```

```
          *         *         *         *         *         *         *         *         *         *
5794>TCCTCCATCTCCGGCGTCGTGACCCGCCGAGCAATGCCGCCTCGGGTCTCGTCCTCAGAGGCGGACTCCGCTCCCTCCTCCTCCTCTTCTTCTTCTTCCT>5893
5773>TCCTCCATCTCCGGCGTCGTGACCCGCCGAGCAATGCCGCCTCGGGTCTCGTCCTCAGAGGCGGACTCCGCTCCCTCCTCCTCCTCTTCTTCTTCTTCCT>5872
5809>TCCTCCATCTCCGGCGTCGTGACCCGCCGAGCAATGCCGCCTCGGGTCTCGTCCTCAGAGGCGGACTCCGCTCCCTCCTCCTCCTCTTCTTCTTCTTCCT>5908
5473>TCCTCCATCTCCGGCGTCGTGACCCGCCGAGCAATGCCGCCTCGGGTCTCGTCCTCAGAGGCGGACTCCGCTCCCTCCTCCTCCTCTTCTTCTTCTTCCT>5572
3662>TCCTCCATCTCCGGCGTCGTGACCCGCCGAGCAATGCCGCCTCGGGTCTCGTCCTCAGAGGCGGACTCCGCTCCCTCCTCCTCCTCTTCTTCTTCTTCCT>3761

          *         *         *         *         *         *         *         *         *         *
5894>CCTCCTCCTCCTCTTCCTCTTCATCTTCGTCCATCTCGTCGTTTAGGGCGGCACTGGCTGCGGGTGGGTCTGCGACGGTGTACATGATGGCGGGGATGTG>5993
5873>CCTCCTCCTCCTCTTCCTCTTCATCTTCGTCCATCTCGTCGTTTAGGGcggcactggcTGCGGGTGGGTCTGCGACGGTGTACATGATGGCGGGGATGTG>5972
5909>CCTCCTCCTCCTCTTCCTCTTCATCTTCGTCCATCTCGTCGTTTAGGGCGGCACTGGCTGCGGGTGGGTCTGCGACGGTGTACATGATGGCGGGGATGTG>6008
5573>CCTCCTCCTCCTCTTCCTCTTCATCTTCGTCCATCTCGTCGTTTAGGGCGGCACTGGCTGCGGGTGGGTCTGCGACGGTGTACATGATGGCGGGGATGTG>5672
3762>CCTCCTCCTCCTCTTCCTCTTCATCTTCGTCCATCTCGTCGTTTAGGGCGGCACTGGCTGCGGGTGGGTCTGCGACGGTGTACATGATGGCGGGGATGTG>3861

          *         *         *         *         *         *         *         *         *         *
5994>GTCGGAGGAGGAGATGGGGTTGGAGGTTGAGTGCTCGGACAAGGCGGGCTTTGGCGGCAGAGGGTGGTGGTGGTCTGCGAAGAATGTCTTCACGTGGTGG>6093
5973>GTCGGAGGAGGAGATGGGGTTGGAGGTTGAGTGCTCGGACAAGGCGGGCTTTGGCGGCAGAGGGTGGTGGTGGTCTGCGAAGAATGTCTTCACGTGGTGG>6072
6009>GTCGGAGGAGGAGATGGGGTTGGAGGTTGAGTGCTCGGACAGGGCGGGCTTTGGCGGCAGAGGGTGGTGGTGGTCTGCGAAGAATGTCTTCACGTGGTGG>6108
5673>GTCGGAGGAGGAGATGGGGTTGGAGGTTGAGTGCTCGGACAGGGCGGGCTTTGGCGGCAGAGGGTGGTGGTGGTCTGCGAAGAATGTCTTCACGTGGTGG>5772
3862>GTCGGAGGAGGAGATGGGGTTGGAGGTTGAGTGCTCGGACAGGGCGGGCTTTGGCGGCAGAGGGTGGTGGTGGTCTGCGAAGAATGTCTTCACGTGGTGG>3961

          *         *         *         *         *         *         *         *         *         *
6094>ATGAAGTTAAGGTCTTCTTGAACCTAAGCAAAAGAGAAAAGAAATGAACATATAGAAGTGAAAACGTTGTTACGTTAAAAGTGATTGGTTACGTTAGGAG>6193
6073>ATGAAGTTAAGGTCTTCTTGAACCTAAGCAAAAGAGAAAAGAAATGAACATATAGAAGTGAAAACGTTGTTACGTTAAAAGTGATTGGTTACGTTAGGAG>6172
6109>ATGAAGTTAAGGTCTTCTTGAACCTAAGCAAAAGAGAAAAGAAATGAACATATAGAAGTGAAAACGTGGTTACGTTAAAAGTGATTGGTTACGTTAGGAG>6208
5773>ATGAAGTTAAGGTCTTCTTGAACCTAAGCAAAAGAGAAAAGAAATGAACATATAGAAGTGAAAACGTTGTTACGTTAAAAGTGATTGGTTACGTTAGGAG>5872
3962>ATGAAGTTAACGTCTTCTTGAACCTAAGCAAAAGAGAAAAGAAATGAACATATAGAAGTGAAAACGTTGTTACGTTAAAAGTGATTGGTTACGTTAGGAG>4061

          *         *         *         *         *         *         *         *         *         *
6194>GGAGTGAAGGTTGGTTACCTTATCCGTTGTACCAAACTCAACGACGCCGTCCAGTAAAGGAATGCACACCACTGTCTGTATGTTACAAATGACAAATCCA>6293
6173>GGAGTGAAGGTTGGTTACCTTATCCGTTGTACCAAACTCAACGACGCCGTCCAGTAAAGGAATGCACACCACTGTCTGTATGTTACAAATGACAAATCCA>6272
6209>GGAGTGAAGGTTGGTTACCTTATCCGTTGTACCAAACTCAACGACGCCGTCCAGTAAAGGAATGCACACCACTGTCTGTATGTTACAAATGACAAATCCA>6308
5873>GGAGTGAAGGTTGGTTACCTTATCCGTTGTACCAAACTCAACGACGCCGTCCAGTAAAGGAATGCACACCACTGTCTGTATGTTACAAATGACAAATCCA>5972
4062>GGAGTGAAGGTTGGTTACCTTATCCGTTGTACCAAACTCAACAACGCCGTCCAGTAAAGGAATGCACACCACTGTCTGTATGTTACAAATGACAAATCCA>4161

          *         *         *         *         *         *         *         *         *         *
6294>TCATATTATATTATATTTATATATTATTCTTCACTTTAATTTTCCTCTTTTATTCATTGGACCAATTATGGATATTTTGAACACGCGTGAAGAACGCAGA>6393
6273>TCATATTATATTATATTTATATATTATTCTTCACTTTAATTTTCCTCTTTTATTCATTGGACCAATTATGGATATTTTGAACACGCGTGAAGAACGCAGA>6372
6309>TCATATTATATTATATTTATATATTATTCTTCACTTTAATTTTCCTCTTTTATTCATTGGACCAATTATGGATATTTTGAACACGCGTGAAGAACGCAGA>6408
5973>TCATATTATATTATATTTATATATTATTCTTCACTTTAATTTTCCTCTTTTATTCATTGGACCAATTATGGATATTTTGAACACGCGTGAAGAACGCAGA>6072
4162>TCATATTATATTACATTTATATATTATTCTTCACTTTAATTTTCCTCTTTTATTCATTGGACCAATTATGGATATTTTGAACACGCGTGAAGAACGCAGA>4261

          *         *         *         *         *         *         *         *         *         *
6394>AATGCGACAATGAGAAAGAAAGATAAAAAAGAATGGTTACCTGTATTCGAGCACTCTATGATTGTAGAAAAGTTTGCATCCAGAAACCAGAGGAAAACAC>6493
6373>AATGCGACAATGAGAAAGAAAGATAAAAAAGAATGGTTACCTGTATTCGAGCACTCTATGATTGTAGAAAAGTTTGCATCCAGAAACCAGAGGAAAACAC>6472
6409>AATGCGACAATGAGAAAGAAAGATAAAAAAGAATGGTTACCTGTATTCGAGCACTCTATGATTGTAGAAAAGTTTGCATCCAGAAACCAGAGGAAAACAC>6508
6073>AATGCGACAATGAGAAAGAAAGATAAAAAAGAATGGTTACCTGTATTCGAGCACTCTATGATTGTAGAAAAGTTTGCATCCAGAAACCAGAGGAAAACAC>6172
4262>AATGCGACAATGAGAAAGAAAGATAAAAAAGAATGGTTACCTGTATTCGAGCACTCTATGATTGTAGAAAAGTTTGCATCCAGAAACCAGAGGAAAACAC>4361

          *         *         *         *         *         *         *         *         *         *
6494>AAGTTAGTAAATTATGAATCACCCTTCATGTCATTCAATAAATTCAAAAGCAAAAGCAACTTGTGTAACTTTTCTTATATCCAAAAGGAAAAAGATTTTA>6593
6473>AAGTTAGTAAATTATGAATCACCCTTCATGTCATTCAATAAATTCAAAAGCAAAAGCAACTTGTGTAACTTTTCTTATATCCAAAAGGAAAAAGATTTTA>6572
6509>AAGTTAGTAAATTATGAATCACCCTTCATGTCATTCAATAAATTCAAAAGCAAAAGCAACTTGTGTAACTTTTCTTATATCCAAAAGGAAAAAGATTTTA>6608
6173>AAGTTAGTAAATTATGAATCACCCTTCATGTCATTCAATAAATTCAAAAGCAAAAGCAACTTGTGTAACTTTTCTTATATCCAAAAGGAAAAAGATTTTA>6272
4362>AAGTTAGTAAATTATGAATCACCCTTCATGTCATTCAATAAATTCAAAAGCAAAAGCAACTTGTGTAACTTTTCTTATATCCAAAAGGAAAAAGATTTTA>4461

          *         *         *         *          *         *         *         *         *         *
6594>TGACACTCCCTTTTGGTTTTAGGTTGACTTGGCAATT-GTTTATAATAGAATATTATATTGACATAATAAAAATATGTAAAAATATAGAATTATAAAATA>6692
6573>TGACACTCCCTTTTGGTTTTAGGTTGACTTGGCAATTGGTTTATAATAGAATATTATATTGACATAATAAAAATATGTAAAAATATAGAATTATAAAATA>6672
6609>TGACACTCCCTTTTGGTTTTAGGTTGACTTGGCAATTGGTTTATAATAGAATATTATATTGACATAATAAAAATATGTAAAAATATAGAATTATAAAATA>6708
6273>TGACACTCCCTTTTGGTTTTAGGTTGACTTGGCAATTGGTTTATAATAGAATATTATATTGACATAATAAAAATATGTAAAAATATAGAATTATAAAATA>6372
4462>TGACACTCCCTTTTGGTTTTAGGTTGACTTGGCAATTGGTTTATAATAGAATATTATATTGACATAATAAAAATATGTAAAAATATAGAATTATAAAATA>4561

           *         *         *         *         *         *         *         *         *         *
6693>ATAATTTATGAAATTATAAGTTGAGTGTAAAAGAAAAATGAAAGTGTCAAACTAACTTGACCCGTTTAAATTCTGCATTATTAAAAATGGTAAGTTTTTT>6792
6673>ATAATTTATGAAATTATAAGTTGAGTGTAAAAGAAAAATGAAAGTGTCAAACTAACTTGACCCGTTTAAATTCTGCATTATTAAAAATGGTAAGTTTTTT>6772
6709>ATAATTTATGAAATTATAAGTTGAGTGTAAAAGAAAAATGAAAGTGTCAAACTAACTTGACCCGTTTAAATTCTGCATTATTAAAAATGGTAAGTTTTTT>6808
6373>ATAATTTATGAAATTATAAGTTGAGTGTAAAAGAAAAATGAAAGTGTCAAACTAACTTGACCCGTTTAAATTCTGCATTATTAAAAATGGTAAGTTTTTT>6472
4562>ATAATTTATGAAATTATAAGTTGAGTGTAAAAGAAAAATGAAAGTGTCAAACTAACTTGACCCGTTTAAATTCTGCATTATTAAAAATGGTAAGTTTTTT>4661
```

```
          *         *         *         *         *         *         *         *         *         *
6793>CCTTAGCTAAGAAAAAGTAATGTTATAATGTTTAAGGAAATTGATTAAATTTCCATTCAATATATTTTATAATAAAGTAGTAATATTTAAATTTTTTAAT>6892
6773>CCTTAGCTAAGAAAAAGTAATGTTATAATGTTTAAGGAAATTGATTAAATTTCCATTCAATATATTTTATAATAAAGTAGTAATATTTAAATTTTTTAAT>6872
6809>CCTTAGCTAAGAAAAAGTAATGTTATAATGTTTAAGGAAATTGATTAAATTTCCATTCAATATATTTTATAATAAAGTAGTAATATTTAAATTTTTTAAT>6908
6473>CCTTAGCTAAGAAAAAGTAATGTTATAATGTTTAAGGAAATTGATTAAATTTCCATTCAATATATTTTATAATAAAGTAGTAATATTTAAATTTTTTAAT>6572
4662>CCTTAGCTAAGAAAAAGTAATGTTATAATGTTTAAGGAAATTGATTAAATTTCCATTCAATATATTTTATAATAAAGTAGTAATATTTAAATTTTTTAAT>4761

          *         *         *         *         *         *         *         *         *         *
6893>ATAATTAATAAACTCTTTTCTTTGGATACTTTCTACTTTTAATAGCATTTGTGTTGAAATATTATGTGAATTGTTTGTTATATAGAAGAAGCACCTTGGC>6992
6873>ATAATTAATAAACTCTTTTCTTTGGATACTTTCTACTTTTAATAGCATTTGTGTTGAAATATTATGTGAATTGTTTGTTATATAGAAGAAGCACCTTGGC>6972
6909>ATAATTAATAAACTCTTTTCTTTGGATACTTTCTACTTTTAATAGCATTTGTGTTGAAATATTATGTGAATTGTTTGTTATATAGAAGAAGCACCTTGGC>7008
6573>ATAATTAATAAACTCTTTTCTTTGGATACTTTCTACTTTTAATAGCATTTGTGTTGAAATATTATGTGAATTGTTTGTTATATAGAAGAAGCACCTTGGC>6672
4762>ATAATTAATAAACTCTTTTCTTTGGATACTTTCTACTTTTAATAGCATTTGTGTTGAAATATTATGTGAATTGTTTGTTATATAGAAGAAGCACCTTGGC>4861

          *         *         *         *         *         *         *         *         *         *
6993>TAGAATAGCTCGTGAAAATGTTTTGCTGTCCACCTCGTTTGCACCAGTGAGCCAAACATGCTGCCTCCTAGCATATGCCTTTCCAGGCAACCTTATAGCG>7092
6973>TAGAATAGCTCGTGAAAATGTTTTGCTGTCCACCTCGTTTGCACCAGTGAGCCAAACATGCTGCCTCCTAGCATATGCCTTTCCAGGCAACCTTATAGCG>7072
7009>TAGAATAGCTCGTGAAAATGTTTTGCTGTCCACCTCGTTTGCACCAGTGAGCCAAACATGCTGCCTCCTAGCATATGCCTTTCCAGGCAACCTTATAGCG>7108
6673>TAGAATAGCTCGTGAAAATGTTTTGCTGTCCACCTCGTTTGCACCAGTGAGCCAAACATGCTGCCTCCTAGCATATGCCTTTCCAGGCAACCTTATAGCG>6772
4862>TAGAATAGCTCGTGAAAATGTTTTGCTGTCCACCTCGTTTGCACCAGTGAGCCAAACATGCTGCCTCCTAGCATATGCCTTTCCAGGCAACCTTATAGCG>4961

          *         *         *         *         *         *         *         *         *         *
7093>CAGTTCAGTAATAGGTGTCAAATGCAAAAAAAATAAATAAAAAAATTAAAAGAATAATATTTTTTCTCAAATTCCATATACTTAAATTATTATACTACTA>7192
7073>CAGTTCAGTAATAGGTGTCAAATGCAAAAAAAATAAATAAAAAAATTAAAAGAATAATATTTTTTCTCAAATTCCATATACTTAAATTATTATACTACTA>7172
7109>CAGTTCAGTAATAGGTGTCAAATGCAAAAAAAATAAATAAAAAAATTAAAAGAATAATATTTTTTCTCAAATTCCATATACTTAAATTATTATACTACTA>7208
6773>CAGTTCAGTAATAGGTGTCAAATGCAAAAAAAATAAATAAAAAAATTAAAAGAATAATATTTTTTCTCAAATTCCATATACTTAAATTATTATACTACTA>6872
4962>CAGTTCAGTAATAGGTGTCAAATGCAAAAAAAATAAATAAAAAAATTAAAAGAATAATATTTTTTCTCAAATTCCATATACTTAAATTATTATACTACTA>5061

          *         *         *         *         *         *         *         *         *         *
7193>CAAGTTTATTAAAAGATATATGTTAGAAATATTGTGAATGTTTACCCCTTAACAGTCTTTTATTAAAGAGAATTCAATGTATGTAACTAAAGTTCCCAGA>7292
7173>CAAGTTTATTAAAAGATATATGTTAGAAATATTGTGAATGTTTACCCCTTAACAGTCTTTTATTAAAGAGAATTCAATGTATGTAACTAAAGTTCCCAGA>7272
7209>CAAGTTTATTAAAAGATATATGTTAGAAATATTGTGAATGTTTACCCCTTAACAGTCTTTTATTAAAGAGAATTCAATGTATGTAACTAAAGTTCCCAGA>7308
6873>CAAGTTTATTAAAAGATATATGTTAGAAATATTGTGAATGTTTACCCCTTAACAGTCTTTTATTAAAGAGAATTCAATGTATGTAACTAAAGTTCCCAGA>6972
5062>CAAGTTTATTAAAAGATATATGTTAGAAATATTGTGAATGTTTACCCCTTAACAGTCTTTTATTAAAGAGAATTCAATGTATGTAACTAAAGTTCCCAGA>5161

          *         *         *         *         *         *         *         *         *         *
7293>ATTGTAGAGAATCTAAACAATCGTCACAAACAATGATTTGCTTCATACAGACAGATGAAGATCCTGATATGTTAAAAGCAAACATAATAATGTCCCACAT>7392
7273>ATTGTAGAGAATCTAAACAATCGTCACAAACAATGATTTGCTTCATACAGACAGATGAAGATCCTGATATGTTAAAAGCAAACATAATAATGTCCCACAT>7372
7309>ATTGTAGAGAATCTAAACAATCGTCACAAACAATGATTTGCTTCATACAGACAGATGAAGATCCTGATATGTTAAAAGCAAACATAATAATGTCCCACAT>7408
6973>ATTGTAGAGAATCTAAACAATCGTCACAAACAATGATTTGCTTCATACAGACAGATGAAGATCCTGATATGTTAAAAGCAAACATAATAATGTCCCACAT>7072
5162>ATTGTAGAGAATCTAAACAATCGTCACAAACAATGATTTGCTTCATACAGACAGATGAAGATCCTGATATGTTAAAAGCAAACATAATAATGTCCCACAT>5261

          *         *         *         *         *         *         *         *         *         *
7393>AATAGAGAAAGATACAAAATGAGTTATATATAGAAAGATCTACAATTATTTATACATAGTTCCTAAATTTAATTTTATATCTTTCTGTCTTATAACAAAC>7492
7373>AATAGAGAAAGATACAAAATGAGTTATATATAGAAAGATCTACAATTATTTATACATAGTTCCTAAATTTAATTTTATATCTTTCTGTCTTATAACAAAC>7472
7409>AATAGAGAAAGATACAAAATGAGTTATATATAGAAAGATCTACAATTATTTATACATAGTTCCTAAATTTAATTTTATATCTTTCTGTCTTATAACAAAC>7508
7073>AATAGAGAAAGATACAAAATGAGTTATATATAGAAAGATCTACAATTATTTATACATAGTTCCTAAATTTAATTTTATATCTTTCTGTCTTATAACAAAC>7172
5262>AATAGAGAAAGATACAAAATGAGTTATATATAGAAAGATCTACAATTATTTATACATAGTTCCTAAATTTAATTTTATATCTTTCTGTCTTATAACAAAC>5361

          *         *         *         *         *         *         *         *         *         *
7493>ACAACATTGTCATGTTTCTTTCTAACATGAAATAATCTAAATCCCACATTAAAATTAGTGCATTATTATTAGATTCCTAATATAGTCCTGCTCCACTCAG>7592
7473>ACAACATTGTCATGTTTCTTTCTAACATGAAATAATCTAAATCCCACATTAAAATTAGTGCATTATTATTAGATTCCTAATATAGTCCTGCTCCACTCAG>7572
7509>ACAACATTGTCATGTTTCTTTCTAACATGAAATAATCTAAATCCCACATTAAAATTAGTGCATTATTATTAGATTCCTAATATAGTCCTGCTCCACTCAG>7608
7173>ACAACATTGTCATGTTTCTTTCTAACATGAAATAATCTAAATCCCACATTAAAATTAGTGCATTATTATTAGATTCCTAATATAGTCCTGCTCCACTCAG>7272
5362>ACAACATTGTCATGTTTCTTTCTAACATGAAATAATCTAAATCCCACATTAAAATTAGTGCATTATTATTAGATTCCTAATATAGTCCTGCTCCACTCAG>5461

          *         *         *         *         *         *         *         *         *         *
7593>CTCTATTAAACTTTTTATTTGTCCTTCTCCACTCATAGCGTCTCCTACAGAATATATATACAAAAGCAAAGGGATCATAGCTTTGTGTGTGTGTGTGAAT>7692
7573>CTCTATTAAACTTTTTATTTGTCCTTCTCCACTCATAGCGTCTCCTACAGAATATATATACAAAAGCAAAGGGATCATAGCTTTGTGTGTGTGTGTGAAT>7672
7609>CTCTATTAAACTTTTTATTTGTCCTTCTCCACTCATAGCGTCTCCTACAGAATATATATACAAAAGCAAAGGGATCATAGCTTTGTGTGTGTGTGTGAAT>7708
7273>CTCTATTAAACTTTTTATTTGTCCTTCTCCACTCATAGCGTCTCCTACAGAATATATATACAAAAGCAAAGGGATCATAGCTTTGTGTGTGTGTGTGAAT>7372
5462>CTCTATTAAACTTTTTATTTGTCCTTCTCCACTCATAGCGTCTCCTACAGAATATATATACAAAAGCAAAGGGATCATAGCTTTGTGTGTGTGTGTGAAT>5561

          *         *         *         *         *         *
7693>GTAGACCAGCCTAAACACACACATCCTATCTCTTATATAATTGAGAACTCAATAGAATGG---------------------------------------->7752
7673>GTAGACCAGCCTAAACACACACATCCTATCTCTTATATAATTGAGAACTCAATAGAATGGTGCAACATGCATTCATCTGTTATATAGGGACCAATTGATG>7772
7709>GTAGACCAGCCTAAACACACACATCCTATCTCTTATATAATTGAGAACTCAATAGAATGGTGCAACATGCATTCATCTGTTATATAGGGACCAATTGATG>7808
7373>GTAGACCAGCCTAAACACACACATCCTATCTCTTATATAATTGAGAACTCAATAGAATGGTGCAACATGCATTCATCTGTTATATAGGGACCAATTGATG>7472
5562>GTAGACCAGCCTAAACACACACATCCTATCTCTTATATAATTGAGAACTCAATAGAATGGTGCAACATGCATTCATCTGTTATATAGGGACCAATTGATG>5661
```

```
7752>---------------------------------------------------------------------------------------------------->7752
7773>TTATAAATCCTTTTTGATGGAAGGGAAAATGGTAGTAGCAATTTGGGAGAAAGTAGGAAGCTATGTAATGTAGCATTGAAACACGGATTCCTATGGTAAA>7872
7809>TTATAAATCCTTTTTGATGGAAGGGAAAATGGTAGTAGCAATTTGGGAGAAAGTAGGAAGCTATGTAATGTAGCATTGAAACACGGATTCCTATGGTAAA>7908
7473>TTATAAATCCTTTTTGATGGAAGGGAAAATGGTAGTAGCAATTTGGGAGAAAGTAGGAAGCTATGTAATGTAGCATTGAAACACGGATTCCTATGGTAAA>7572
5662>TTATAAATCCTTTTTGATGGAAGGGAAAATGGTAGTAGCAATTTGGGAGAAAGTAGGAAGCTATGTAATGTAGCATTGAAACACGGATTCCTATGGTAAA>5761

7752>---------------------------------------------------------------------------------------------------->7752
7873>TAATTATTCCATAATGAAAATATGATGATGGTGGAAAAAGTTTGATCCCGGAGGAGGGAGAAGAAAAGGAAGATGGAGGTGTGATTGAAAAAAGTTCCAA>7972
7909>TAATTATTCCATAATGAAAATATGATGATGGTGCAAAAAGTTTGATCCCGGAGGAGGGAGAAGAAAAGGAAGATGGAGGTGTGATTGAAAAAAGTTCCAA>8008
7573>TAATTATTCCATAATGAAAATATGATGATGGTGGAAAAAGTTTGATCCCGGAGGAGGGAGAAGAAAAGGAAGATGGAGGTGTGATTGAAAAAAGTTCCAA>7672
5762>TAATTATTCCATAATGAAAATATGATGATGGTGGAAAAAGTTTGATCCCGGAGGAGGGAGAAGAAAAGGAAGATGGAGGTGTGATTGAAAAAAGTTCCAA>5861

7752>---------------------------------------------------------------------------------------------------->7752
7973>ATGAGGATGGTAGCTACAGTGGAATTTTTTTGCAGCTATATCGCTTTAAATTTGAACGCTTTAATTTGTCTGGTTTCTCAAAATAGGCAATTCCCTCTAG>8072
8009>ATGAGGATGGTAGCTACAGTGGAATTTTTTTGCAGCTATATCGCTTTAAATTTGAACGCTTTAATTTGTCTGGTTTCTCAAAATAGGCAATTCCCTCTAG>8108
7673>ATGAGGATGGTAGCTACAGTGGAATTTTTTTGCAGCTATATCGCTTTAAATTTGAACGCTTTAATTTGTCTGGTTTCTCAAAATAGGCAATTCCCTCTAG>7772
5862>ATGAGGATGGTAGCTACAGTGGAATTCTTTTGCAGCTATATCGCTTTAAATTTGAACGCTTTAATTTGTCTGGTTTCTCAAAATAGGCAATTCCCTCTAG>5961

7752>---------------------------------------------------------------------------------------------------->7752
8073>CAGCAGTCCTATTCAAGCATATAATGCGCAACTAAATTTTGACCAGCTCAGCAAATTCTTTTTTGACAGAATGCTCCGAATATATTCCCCAACACATTCT>8172
8109>CAGCAGTCCTATTCAAGCATATAATGCGCAACTAAATTTTGACCAGCTCAGCAAATTCTTTTTTGACAGAATGCTCCGAATATATTCCCCAACACATTCT>8208
7773>CAGCAGTCCTATTCAAGCATATAATGCGCAACTAAATTTTGACCAGCTCAGCAAATTCTTTTTTGACAGAATGCTCCGAATATATTCCCCAACACATTCT>7872
5962>CAGCAGTCCTATTCAAGCATATAATGCGCAACTAAATTTTGACCAGCTCAGCAAATTCTTTTTTGACAGAATGCTCCGAATATATTCCCCAACACATTCT>6061

7752>---------------------------------------------------------------------------------------------------->7752
8173>AACACAACTCTCAATATCAATGCTGTCAGTTTGACAAATTTACGTAATCTAACTGTGCTTCATTTACTCCTGAAAGCAGAATCTGAGAAACCTTCGAATA>8272
8209>AACACAACTCTCAATATCAATGCTGTCAGTTTGACAAATTTACGTAATCTAACTGTGCTTCATTTACTCCTGAAAGCAGAATCTGAGAAACCTTCGAATA>8308
7873>AACACAACTCTCAATATCAATGCTGTCAGTTTGACAAATTTACGTAATCTAACTGTGCTTCATTTACTCCTGAAAGTAGAATCTGAGAAACCTTCGAATA>7972
6062>AACACAACTCTCAATATCAATGCTGTCAGTTTGACAAATTTACGTAATCTAACTGTGCTTCATTTACTCCTGAAAGCAGAATCTGAGAAACCTTCGAATA>6161

7752>---------------------------------------------------------------------------------------------------->7752
8273>GGAAGAAGAAATAAAGTCCCTCAACCACCTTTTGGTACGTAGAGACAAATGATACCATTCGTCAAACACTTCCAACAAAAAAAGGTGAAAATTGCACAAC>8372
8309>GGAAGAAGAAATAAAGTCCCTCAACCACCTTTTGGTACGTAGAGACAAATGATACCATTCGTCAAACACTTCCAACAAAAAAAGGTGAAAATTGCACAAC>8408
7973>GGAAGAAGAAATAAAGTCCCTCAACCACCTTTTGGTACGTAGAGACAAATGATACCATTCGTCAAACACTTCCAACAAAAAAAGGTGAAAATTGCACAAC>8072
6162>GGAAGAAGAAATAAAGTCCCTCAACCACCTTTTGGTACGTAGAGACAAATGATACCATTCGTCAAACACTTCCAACAAAAAAAGGTGAAAATTGCACAAC>6261

7752>---------------------------------------------------------------------------------------------------->7752
8373>TTTTACCGTCTCTTCTCTTTTTCCACACAAAATATCAAATTCCTTTCACTATACAATCTCTGCACTGCTATATCTTTTTCAACAACATAAAATGACACCA>8472
8409>TTTTACCGTCTCTTCTCTTTTTCCACACAAAATATCAAATTCCTTTCACTATACAATCTCTGCACTGCTATATCTTTTTCAACAACATAAAATGACACCA>8508
8073>TTTTACCGTCTCTTCTCTTTTTCCACACAAAATATCAAATTCCTTTCACTATACAATCTCTGCACTGCTATATCTTTTTCAACAACATAAAATGACACCA>8172
6262>TTTTACCGTCTCTTCTCTTTTTCCACACAAAATATCAAATTCCTTTCACTATACAATCTCTGCACTGCTATATCTTTTTCAACAACATCTCTCTCATACT>6361

7752>---------------------------------------------------------------------------------------------------->7752
8473>GTTGCACAACTCATTCTTATAATTTTGTAAATTTTATCAAATATATGTAAATCTTAGAGAGGGAATCAAAGAAAGCTTGTTATATACATTGGCATTTTCT>8572
8509>GTTGCACAACTCATTCTTATAATTTTGTAAATTTTATCAAATATATGTAAATCTTAGAGAGGGAATCAAAGAAAGCTTGTTATATACATTGGCATTTTCT>8608
8173>GTTGCACAATCATTCTTATAATTTTGTAAATTTTATCAAATATATGTAAATCTTAGAGAGGGAATCAAAGAAAGCTTGTTATATACATTGGCATTTTCTT>8272
6362>ATATCGACTCAAATGTGTAATGATTCATAAAATGACACCAGTTGCACAACTCATTCTTATAATTTTGTAAATTTTATGAAATATATGTAAATCTTAGAGA>6461

7752>---------------------------------------------------------------------------------------------------->7752
8573>TTTCAGTCTTTACTGACACTTCATAAATTCGAAGCTACTCAGATTGCATTTAAACTACACTCAGAGAAGCTCTGGGCCAGTCTTGATGCCTCACCAAAAT>8672
8609>TTTCAGTCTTTACTGACACTTCATAAATTCGAAGCTACTCAGATTGCATTTAAACTACACTCAGAGAAGCTCTGGGCCAGTCTTGATGCCTCACCAAAAT>8708
8273>TTCAGTCTTTACTGACACTTCATAAATTCGAAGCTACTCAGATTGCATTTAAACTACACTCAGAGAAGCTCTGGGCCAGTCTTGATGCCTCACCAAAATG>8372
6462>GGGAATCAAAGAAAGCTTGTTATATACATTGGCATTTTCTTTTCAGTCTTTACTGACACTTCATAAATTCGAAGCTATTCAGATTGCATTTAAACTACAC>6561

7752>---------------------------------------------------------------------------------------------------->7752
8673>GCACTTCCAAGTGAGGGAAATCGCATTGATACTTCATTGACAACCTCTAGCATAACCTCTTTATGAGAAAATGATTTAACATCTTTAATTTTCTCTAAAC>8772
8709>GCACTTCCAAGTGAGGGAAATCGCATTGATACTTCATTGACAACCTCTAGCATAACCTCTTTATGAGAAAATGATTTAACATCTTTAATTTTCTCTAAAC>8808
8373>CACTTCCAAGTGAGGGAAATCGCATTGATACTTCATTGACAACCTCTAGCATAACCTCTTTATGAGAAAATGATTTAACATCTTTAATTTTCTCTAAACA>8472
6562>TCAGAGAAGCTCTGGGCCAGTCTTGATGCCTCACCAAAATGCACTTCCAAGTGAGGGAAATCGCATTGATACTTCATTGACAACCTCTAGCATAACCTCT>6661
```

```
                                                                                                        *
7753>---------------------------------------------------------------------------------------AAAGTTATTAAAT>7765
8773>AAAGTAAGGAAGGCGCTTGTTTTCAAAAGCTAAGTTAGCACAAATGG----------------------------------------AAAGTTATTAAAT>8832
8809>AAAGTAAGGAAGGCGCTTGTTTTCAAAAGCTAAGTTAGCACAAATGG----------------------------------------AAAGTTATTAAAT>8868
8473>AAGTAAGGAAGGCGCTTGTTTTCAAAAGCTAAGTTAGCACAAATGG-----------------------------------------AAAGTTATTAAAT>8531
6662>TTATGAGAAAATGATTTAACATCTTTAATTTTCTCTAAACAAAGTAAGGAAGGCGCTTGTTTTCAAAAGCTAAGTTAGCACAAATGGAAAGTTATTAAAT>6761

              *         *         *         *         *         *         *         *         *         *
7766>TTTTGCAGCTGCTATCGTTGCATTTAAATTCACACGTCACAGTAGCTAGTCTCTCCCTTTCTCTTTTACTATGGGACACTATAATACTTCTCCTCACCCT>7865
8833>TTTTGCAGCTGCTATCGTTGCATTTAAATTCACACGTCACAGTAGCTAGTCTCTCCCTTTCTCTTTTACTATGGGACACTATAATACTTCTCCTCACCCT>8932
8869>TTTTGCAGCTGCTATCGTTGCATTTAAATTCACACGTCACAGTAGCTAGTCTCTCCCTTTCTCTTTTACTATGGGACACTATAATACTTCTCCTCACCCT>8968
8532>TTTTGCAGCTGCTATCGTTGCATTTAAATTCACACGTCACAGTAGCTAGTCTCTCCCTTTCTCTTTTACTATGGGACACTATAATACTTCTCCTCACCCT>8631
6762>TTTTGCAGCTGCTATCGTTGCATTTAAATTCACACGTCACAGTAGCTAGTCTCTCCCTTTCTCTTTTACTATGGGACACTATAATACTTCTCCTCACCCT>6861

              *         *         *         *         *         *         *         *         *         *
7866>TTACAAAAGCTACCGTTCCACTTATCTTCAAAAGGTAATAATTAACTCAAATTTTGAACCCAAACTATACATATCTAGAAAATGGCCTATCAGATAATAA>7965
8933>TTACAAAAGCTACCGTTCCACTTATCTTCAAAAGGTAATAATTAACTCAAATTTTGAACCCAAACTATACATATCTAGAAAATGGCCTATCAGATAATAA>9032
8969>TTACAAAAGCTACCGTTCCACTTATCTTCAAAAGGTAATAATTAACTCAAATTTTGAACCCAAACTATACATATCTAGAAAATGGCCTATCAGATAATAA>9068
8632>TTACAAAAGCTACCGTTCCACTTATCTTCAAAAGGTAATAATTAACTCAAATTTTGAACCCAAACTATACATATCTAGAAAATGGCCTATCAGATAATAA>8731
6862>TTACAAAAGCTACCGTTCCACTTATCTTCAAAAGGTAATAATTAACTCAAATTTTGAACCCAAACTATACATATCTAGAAAATGGCCTATCAGATAATAA>6961

              *         *         *         *         *         *         *         *         *         *
7966>AATTCACCACATCAAATATAGTTCAACATTATTAAAAAGAGTCTTAAAGAACTGATAAAGTTAAAGTTAGATTAGATTTCTATGTAAAGACATAAAGAGA>8065
9033>AATTCACCACATCAAATATAGTTCAACATTATTAAAAAGAGTCTTAAAGAACTGATAAAGTTAAAGTTAGATTAGATTTCTATGTAAAGACATAAAGAGA>9132
9069>AATTCACCACATCAAATATAGTTCAACATTATTAAAAAGAGTCTTAAAGAACTGATAAAGTTAAAGTTAGATTAGATTTCTATGTAAAGACATAAAGAGA>9168
8732>AATTCACCACATCAAATATAGTTCAACATTATTAAAAAGAGTCTTAAAGAACTGATAAAGTTAAAGTTAGATTAGATTTCTATGTAAAGACATAAAGAGA>8831
6962>AATTCACCACATCAAATATAGTTCAACATTATTAAAAAGAGTCTTAAAGAACTGATAAAGTTAAAGTTAGATTAGATTTCTATGTAAAGACATAAAGAGA>7061

              *         *         *         *         *         *         *         *         *         *
8066>GCTTTATGACAAATAATGCAGCATGACTTACCCAACACCAGGAGGAAAGGAGAATGAGACACACATCAAATAGAACCATTCAGATTCTGTCAAGTCCTCT>8165
9133>GCTTTATGACAAATAATGCAGCATGACTTACCCAACACCAGGAGGAAAGGAGAATGAGACACACATCAAATAGAACCATTCAGATTCTGTCAAGTCCTCT>9232
9169>GCTTTATGACAAATAATGCAGCATGACTTACCCAACACCAGGAGGAAAGGAGAATGAGACACACATCAAATAGAACCATTCAGATTCTGTCAAGTCCTCT>9268
8832>GCTTTATGACAAATAATGCAGCATGACTTACCCAACACCAGGAGGAAAGGAGAATGAGACACACATCAAATAGAACCATTCAGATTCTGTCAAGTCCTCT>8931
7062>GCTTTATGACAAATAATGCAGCATGACTTACCCAACACCAGGAGGAAAGGAGAATGAGACACACATCAAATAGAACCATTCAGATTCTGTCAAGTCCTCT>7161

              *         *         *         *         *         *         *         *         *         *
8166>GGCGACAAGGCGGCGCAAGGCCGTCGAGTTGGCGGATTTGTCTCTCCGGCAGACAGTGATTCATACAGTTCTCTAAGTTGTTGGCTTCTTTGGAGAGATG>8265
9233>GGCGACAAGGCGGCGCAAGGCCGTCGAGTTGGCGGATTTGTCTCTCCGGCAGACAGTGATTCATACAGTTCTCTAAGTTGTTGGCTTCTTTGGAGAGATG>9332
9269>GGCGACAAGGCGGCGCAAGGCCGTCGAGTTGGCGGATTTGTCTCTCCGGCAGACAGTGATTCATACAGTTCTCTAAGTTGTTGGCTTCTTTGGAGAGATG>9368
8932>GGCGACAAGGCGGCGCAAGGCCGTCGAGTTGGCGGATTTGTCTCTCCGGCAGACAGTGATTCATACAGTTCTCTAAGTTGTTGGCTTCTTTGGAGAGATG>9031
7162>GGCGACAAGGCGGCGCAAGGCCGTCGAGTTGGCGGATTTGTCTCTCCGGCAGACAGTGACTCATACAGTTCTCTAAGTTGTTGGCTTCTTTGGAGAGATG>7261

              *         *         *         *         *         *         *         *         *         *
8266>CCTCCTCAGCACTCACCTCCATTGGTTGCACCGTCTTCCTAGTCTTGATTGCCCCATTGTAGTACCCATCACCCCAAACCAGAATCCTAAATCAACATAA>8365
9333>CCTCCTCAGCACTCACCTCCATTGGTTGCACCGTCTTCCTAGTCTTGATTGCCCCATTGTAGTACCCATCACCCCAAACCAGAATCCTAAATCAACATAA>9432
9369>CCTCCTCAGCACTCACCTCCATTGGTTGCACCGTCTTCCTAGTCTTGATTGCCCCATTGTAGTACCCATCACCCCAAACCAGAATCCTAAATCAACATAA>9468
9032>CCTCCTCAGCACTCACCTCCATTGGTTGCACCGTCTTCCTAGTCTTGATTGCCCCATTGTAGTACCCATCACCCCAAACCAGAATCCTAAATCAACATAA>9131
7262>CCTCCTCAGCACTCACCTCCATTGGTTGCACCGTCTTCCTAGTCTTGATTGCCCCATTGTAGTACCCATCACCCCAAACCAGAATCCTAAATCAACATAA>7361

              *         *         *         *         *         *         *         *         *         *
8366>CATATAACACATCACCCCAAACCAGAATCCTAAATCAACATAACATATAACACATCACCTTAAAACTAACTTGTTATAATTTAGTGTCACACACATATAC>8465
9433>CATATAA-------------------------------------------CACATCACCTTAAAACTAACTTGTTATAATTTAGTGTCACACACATATAC>9489
9469>CATATAA-------------------------------------------CACATCACCTTAAAACTAACTTGTTATAATTTAGTGTCACACACATATAC>9525
9132>CATATAA-------------------------------------------CACATCACCTTAAAACTAACTTGTTATAATTTAGTGTCACACACATATAC>9188
7362>CATATAA-------------------------------------------CACATCACCTTAAAACTAACTTGTTATAATTTAGTGTCACACACATATAC>7418

              *         *         *         *         *         *         *         *         *         *
8466>ATGTGTCGGTTAGAGAGAGTGAGAAACATTAATTAGAGGAGTATGAGTGATTATATATGATATGGGACAATTAGTGTGGATAAAGTGAATTAATAACGAC>8565
9490>ATGTGTCGGTTAGAGAGAGTGAGAAACATTAATTAGAGGAGTATGAGTGATTATATATGATATGGGACAATTAGTGTGGATAAAGTGAATTAATAACGAC>9589
9526>ATGTGTCGGTTAGAGAGAGTGAGAAACATTAATTAGAGGAGTATGAGTGATTATATATGATATGGGACAATTAGTGTGGATAAAGTGAATTAATAACGAC>9625
9189>ATGTGTCGGTTAGAGAGAGTGAGAAACATTAATTAGAGGAGTATGAGTGATTATATATGATATGGGACAATTAGTGTGGATAAAGTGAATTAATAACGAC>9288
7419>ATGTGTCGGTTAGAGAGAGTGAGAAACATTAATTAGAGGAGTATGAGTGATTATATATGATATGGGACAATTAGTGTGGATAAAGTGAATTAATAACGAC>7518

              *         *         *         *         *         *         *         *         *         *
8566>GTACACTTGTTGTGGGCAAAGTTGCCAGAAGAGGCTATAAGTCCAGTGAACAGATTGCACCGCAGCCTGCAACATGCTTTGGAGCCTATTGCCTAGTGGT>8665
9590>GTACACTTGTTGTGGGCAAAGTTGCCAGAAGAGGCTATAAGTCCAGTGAACAGATTGCACCGCAGCCTGCAACATGCTTTGGAGCCTATTGCCTAGTGGT>9689
9626>GTACACTTGTTGTGGGCAAAGTTGCCAGAAGAGGCTATAAGTCCAGTGAACAGATTGCACCGCAGCCTGCAACATGCTTTGGAGCCTATTGCCTAGTGGT>9725
9289>GTACACTTGTTGTGGGCAAAGTTGCCAGAAGAGGCTATAAGTCCAGTGAACAGATTGCACCGCAGCCTGCAACATGCTTTGGAGCCTATTGCCTAGTGGT>9388
7519>GTACACTTGTTGTGGGCAAAGTTGCCAGAAGAGGCTATAAGTCCAGTGAACAGATTGCACCGCAGCCTGCAACATGCTTTGGAGCCTATTGCCTAGTGGT>7618
```

```
          *         *         *         *         *         *         *         *         *         *
 8666>GCAGCCATTGTTCCTAACAGGTTTCGGAACAGAAGGCTTTTGGTTACTTCTCTGGAACCATGTCTACTCTGCTTCAAAAAACATACCCAAAAGTGGGAAA>8765
 9690>GCAGCCATTGTTCCTAACAGGTTTCGGAACAGAAGGCTTTTGGTTACTTCTCTGGAACCATGTCTACTTTGCTTCAAAAAACATACCCAAAAGTgggaaa>9789
 9726>GCAGCCATTGTTCCTAACAGGTTTCGGAACAGAAGGCTTTTGGTTACTTCTCTGGAACCATGTCTACTCTGCTTCAAAAAACATACCCAAAAGTGGGAAA>9825
 9389>GCAGCCATTGTTCCTAACAGGTTTCGGAACAGAAGGCTTTTGGTTACTTCTCTGGAACCATGTCTACTCTGCTTCAAAAAACATACCCAAAAGTGGGAAA>9488
 7619>GCAGCCATTGTTCCTAACAGGTTTCGGAACAGAAGGCTTTTGGTTACTTCTCTGGAACCATGTCTACTTTGCTTCAAAAAACATACCCAAAAGTGGGAAA>7718

          *         *         *         *         *         *         *         *         *         *
 8766>AAGGTGAGAAAAAGGAAAAAAAAAAAAAAAAGGGTTCTTGGTTTTGATGAGAAGTTAATAGGCAATGAAAAAAAAAGATTGTTTTGCGTGGATCTGAGCC>8865
 9790>aaggtGAGAAAAA---------AAAAAAAAAGGGTTCTTGGTTTTGATGAGAAGTTAATAGGCAATGAAAAAAAAAGATTGTTTTGCGTGGATCTGAGCC>9880
 9826>AAGgtgagaaaaagGAAAAAAAAAAAAAAAAGGGTTCTTGGTTTTGATGAGAAGTTAATAGGCAATGAAAAAAAAAGATTGTTTTGCGTGGATCTGAGCC>9925
 9489>AAGGTGAGAAAAAGgaaaaaaaaaaAAAAAAGGGTTCTTGGTTTTGATGAGAAGTTAATAGGCAATGAAAAAAAAAGATTGTTTTGCGTGGATCTGAGCC>9588
 7719>AAGGTGAGaaaaaggaaaaAAAAA-AAAAAAGGGTTCTTGGTTTTGATGAGAAGTTAATAGGCAATGAAAAAAAAAGATTGTTTTGCGTGGATCTGAGCC>7817

          *         *         *         *         *         *         *         *         *         *
 8866>GAAAATCAAACCTTTAATTTATTGGGAGTGAAAGGAAGGGAGAAAATAAGGTAAAGGGAGTTGTGAAGAGAGAAGAGAAGGTCAAGAAATTAAGTATAGA>8965
 9881>GAAAATCAAACCTTTAATTTATTGGGAGTGAAAGGAAGGGAGAAAATAAGGTAAAGGGAGTTGTGAAGAGAGAAGAGAAGGTCAAGAAATTAAGTATAGA>9980
 9926>GAAAATCAAACCTTTAATTTATTGGGAGTGAAAGGAAGGGAGAAAATAAGGTAAAGGGAGTTGTGAAGAGAGAAGAGAAGGTCAAGAAATTAAGTATAGA>10025
 9589>GAAAATCAAACCTTTAATTTATTGGGAGTGAAAGGAAGGGAGAAAATAAGGTAAAGGGAGTTGTGAAGAGAGAAGAGAAGGTCAAGAAATTAAGTATAGA>9688
 7818>GAAAATCAAACCTTTAATTTATTGGGAGTGAAAGGAAGGGAGAAAATAAGGTAAAGGGAGTTGTGAAGAGAGAAGAGAAGGTCAAGAAATTAAGTATAGA>7917

          *         *         *         *         *         *         *         *         *
 8966>CCTGCAGATGGATGTGTGGTTTGCGTATTGGAGAGAAAGAGATGTTTAAAAAGGTGTATGACTTTGAGGTGGATATGCGGGCGATCCAAGA--------->9056
 9981>CCTGCAGATGGATGTGTGGTTTGCGTATTGGAGAGAAAGAGATGTTTAAAAAGGTGTATGACTTTGAGGTGGATATGCGGGCGATCCAAGA--------->10071
10026>CCTGCAGATGGATGTGTGGTTTGCGTATTGGAGAGAAAGAGATGTTTAAAAAGGTGTATGACTTTGAGGTGGATATGCGGGCGATCCAAGATGTTAGGAA>10125
 9689>CCTGCAGATGGATGTGTGGTTTGCGTATTGGAGAGAAAGAGATGTTTAAAAAGGTGTATGACTTTGAGGTGGATATGCGGGCGATCCAAGA--------->9779
 7918>CCTGCAGATGGATGTGTGGTTTGCGTATTGGAGAGAAAGAGATGTTTAAAAAGGTGTATGACTTTGAGGTGGATATGCGGGCGATCCAAGA--------->8008

 9056>---------------------------------------------------------------------------------------------------->9056
10071>---------------------------------------------------------------------------------------------------->10071
10126>TTCAAGTGTGAGTCTAAGTCCCACATTGACCAGAAATGAGAAAGTAGAGCACTATATAAGAATAAAGACCCATAAACCCATTGCCTTAAGGTTTTGGGTT>10225
 9779>---------------------------------------------------------------------------------------------------->9779
 8008>---------------------------------------------------------------------------------------------------->8008

                                                                                                    *
 9057>--------------------------------------------------------------------------------------------ATTACCAA>9064
10072>--------------------------------------------------------------------------------------------ATTACCAA>10079
10226>GAGAGTGGTGTCAATGTCTTATGTGGTTGGGCTCAGGTCTCATTGGTGTTGTTCTCCCCGGTGAAACCCCCTCTGTAAAACCTGACACAAGAATTACCAA>10325
 9780>--------------------------------------------------------------------------------------------ATTACCAA>9787
 8009>--------------------------------------------------------------------------------------------ATTACCAA>8016

           *         *         *         *         *         *         *         *         *         *
 9065>ATCTCTGTAAGGAGAAGATCTAGGAACGTTTGTTGGTTAATGATCAGAAAGAGAGAGGGAGAGAGAGAAATAGGGTTTTGAAACTGAAGAAAGTGTATGG>9164
10080>ATCTCTGTAAGGAGAAGATCTAGGAACGTTTGTTGGTTAATGATCAGAAAGAGAGAGGGAGAGAGAGAAATAGGGTTTTGAAACTGAAGAAAGTGTATGG>10179
10326>ATCTCTGTAAGGAGAAGATCTAGGAACGTTTGTTGGTTAATGATCAGAAAGAGAGAGGGAGAGAGAGAAATAGGGTTTTGAAACTGAAGAAAGTGTATGG>10425
 9788>ATCTCTGTAAGGAGAAGATCTAGGAACGTTTGTTGGTTAATGATCAGAAAGAGAGAGGGAGAGAGAGAAATAGGGTTTTGAAACTGAAGAAAGTGTATGG>9887
 8017>ATCTCTGTAAGGAGAAGATCTAGGAACGTTTGTTGGTTAATGATCAGAAAGAGAGAGGGAGAGAGAGAAATAGGGTTTTGAAACTGAAGAAAGTGTATGG>8116

           *         *         *         *         *         *         *         *         *         *
 9165>TTTTGACTGGTTGGTCTACCAGACAGTTGTAGCTAGCTATGTGGTTTTTGAATGATTAGACTCTGAGGCTGCCTTGTGCTTTGTTAGTTGTGTTTGTTTT>9264
10180>TTTTGACTGGTTGGTCTACCAGACAGTTGTAGCTAGCTATGTGGTTTGTGAATGATTAGACTCTGAGGCTGCCTTGTGCTTTGTTAGTTGTGTTTGTTTT>10279
10426>TTTTGACTGGTTGGTCTACCAGACAGTTGTAGCTAGCTATGTGGTTTTTGAATGATTAGACTCTGAGGCTGCCTTGTGCTTTGTTAGTTGTGTTTGTTTT>10525
 9888>TTTTGACTGGTTGGTCTACCAGACAGTTGTAGCTAGCTATGTGGTTTTTGAATGATTAGACTCTGAGGCTGCCTTGTGCTTTGTTAGTTGTGTTTGTTTT>9987
 8117>TTTTGACTGGTTGGTCTACCAGACAGTTGTAGCTAGCTATGTGGTTTGTGAATGATTAGACTCTGAGGCTGCCTTGTGCTTTGTTAGTTGTGTTTGTTTT>8216

           *         *         *         *         *         *         *         *         *         *
 9265>GGAGAGCCTTCAGGATTTTGGTACTGAGAAGGAAAACAAAATGATTTTCTAAAATGTATTTGGGTACATATAACTTGATGAGTAAAAGACAGATAGAAAG>9364
10280>GGAGAGCCTTCAGGATTTTGGTACTGAGAAGGAAAACAAAATGATTTTCTAAAATGTATTTGGGTACATATAACTTGATGAGTAAAAGACAGATAGAAAG>10379
10526>GGAGAGCCTTCAGGATTTTGGTACTGAGAAGGAAAACAAAATGATTTTCTAAAATGTATTTGGGTACATATAACTTGATGAGTAAAAGACAGATAGAAAG>10625
 9988>GGAGAGCCTTCAGGATTTTGGTACTGAGAAGGAAAACAAAATGATTTTCTAAAATGTATTTGGGTACATATAACTTGATGAGTAAAAGACAGATAGAAAG>10087
 8217>GGAGAGCCTTCAGGATTTTGGTACTGAGAAGGAAAACAAAATGATTTTCTAAAATGTATTTGGGTACATATAACTTGATGAGTAAAAGACAGATAGAAAG>8316

           *         *         *         *         *         *         *         *         *         *
 9365>GAACCATGTGTGTGTAACAAATACTAACAACAATAGTTATAATGAAAGAAAAATTAAAATTGAAATAGGTGCGTGGAAATCAAGCACCGGCATGTCATGA>9464
10380>GAACCATGTGTGTGTAACAAATACTAACAACAATAGTTATAATGAAAGAAAAATTAAAATTGAAATAGGTGCGTGGAAATCAAGCACCGGCATGTCATGA>10479
10626>GAACCATGTGTGTGTAACAAATACTAACAACAATAGTTATAATGAAAGAAAAATTAAAATTGAAATAGGTGCGTGGAAATCAAGCACCGGCATGTCATGA>10725
10088>GAACCATGTGTGTGTAACAAATACTAACAACAATAGTTATAATGAAAGAAAAATTAAAATTGAAATAGGTGCGTGGAAATCAAGCACCGGCATGTCATGA>10187
 8317>GAACCATGTGTGTGTAACAAATACTAACAACAATAGTTATAATGAAAGAAAAATTAAAATTGAAATAGGTGCGTGGAAATCAAGCACTGGCATGTCATGA>8416
```

```
            *         *         *         *         *         *         *         *            *         *
 9465>AAAAATCGTGAGGATTATGTCAACCCACTAAATTCTAATCAAAACTAGGGGTGATAAAAATATCTCTTTACGTGAATATTTGCTA---ATAAAATCCTTT>9561
10480>AAAAATCGTGAGGATTATGTCAACCCACTAAATTCTAATCAAAACTAGGGGTGATAAAAATATCTCTTTACGTGAATATTTGCTA---ATAAAATCCTTT>10576
10726>AAAAATCGTGAGGATTATGTCAACCCACTAAATTCTAATCAAAACTAGGGGTGATAAAAATATCTCTTTACGTGAATATTTGCTA---ATAAAATCCTTT>10822
10188>AAAAATCGTGAGGATTATGTCAACCCACTAAATTCTAATCAAAACTAGGGGTGATAAAAATATCTCTTTACGTGAATATTTGCTA---ATAAAATCCTTT>10284
 8417>AAAAATCGTGAGGATTATGTCAACCCACTAAATTCTAATCAAAACTAGGTGTGATAAAAATATCTGTTTACGTGAATATTTGCTAATTATAAAATCCTTT>8516

               *         *         *         *         *         *         *         *         *         *
 9562>AGGGTAATGAGGGTATATTTTAAATTAAGGTTGGTAATACAAACCGGCTTGTCCCGTTTTGGTAGGGGTGGTAAATTGGGCCAGTCTCGTCCATTTTCGC>9661
10577>AGGGTAATGAGGGTATATTTTAAATTAAGGTTGGTAATACAAACCGGCTTGTCCCGTTTTGGTAGGGGTGGTAAATTGGGCCAGTCTCGTCCATTTTCGC>10676
10823>AGGGTAATGAGGGTATATTTTAAATTAAGGTTGGTAATACAAACCGGCTTGTCCCGTTTTGGTAGGGGTGGTAAATTGGGCCAGTCTCGTCCATTTTCGC>10922
10285>AGGGTAATGAGGGTATATTTTAAATTAAGGTTGGTAATACAAACCGGCTTGTCCCGTTTTGGTAGGGGTGGTAAATTGGGCCAGTCTCGTCCATTTTCGC>10384
 8517>AGGGTAATGAGGGTATATTTTAAATTAAGGTTGGTAATACAAACCGGCTTGTCCCGTTTTGGTAGGGGTGATAAATTGGGCCAGTCCCGTCCATTTTGGC>8616

               *         *         *         *         *         *         *         *         *         *
 9662>TCGGCCCACCTGTGGCTCGCCAAAAACGGGCTGGGTCGGGCTGGCCCGCCATCCAAAACGGGTTGGATATTGTAACCCGTCCCGTTTTAGGGCGGGTTGG>9761
10677>TTGGCCCACCTGTGGCTCGCCAAAAACGGACTGGGTCGGGCTGGCCCGCCATCCAAAACGGGTTGGATATTGTAACCCGTCCCGTTTTAGGGCGGGTTGG>10776
10923>TCGGCCCACCTGTGGCTCGCCAAAAACGGGCTGGGTCGGGCTGGCCCGCCATCCAAAACGGGTTGGATATTGTAACCCGTCCCGTTTTAGGGCGGGTTGG>11022
10385>TCGGCCCACCTGTGGCTCGCCAAAAACGGGCTGGGTCGGGCTGGCCCGCCATCCAAAACGGGTTGGATATTGTAACCCGTCCCGTTTTAGGGCGGGTTGG>10484
 8617>TCGGCTCGCCTGTGGCTCGCCAAAAACGGGTTGGGTCGGGCTGGCCCGCCATCCAAAACGGGTTGGATATTGTAACCCGTCCCGTTTTAGGGTGGGTTGG>8716

               *         *         *         *         *          *         *         *         *         *
 9762>CGGGTCCGCGGACTGGCTTGTTTTTTAATTTTTTTTTAATTTTTTTAAA-TTTTTTTAACTTTTATAAATTTTGTTTATAAATCTATTATATATATAT>9860
10777>CGGGTCCGCGGACTGGCTTGTTTTTTAATTTTTTTT-AATTTTTTTTAA-TTTTTTTAACTTTTATAAATTTTGTTTATAAATCTATTATATAT---->10870
11023>CGGGTCCGCGGACTGGCTTGTTTTTTAATTTTTTTTTAATTTTTTTAAA-TTTTTTTAACTTTTATAAATTTTGTTTATAAATCTATTATATATATAT>11121
10485>CGGGTCCGCGGACTGGCTTGTTTTTTAATTTTTTTTTAATTTTTTTAAAATTTTTTTAACTTTTATAAATTTTGTTTATAAATCTATTATATATATAT>10584
 8717>CGGACCTGCAGGCTGGCCTGTTTTTTAATTTTT-TTTAATTTTTTTAAA-TTTTTTTAACTTTTATAAATTTTGTTTATAAATCTATTATATATATAT>8814

                *                   *         *         *         *         *         *         *         *
 9861>ATATATATAT----------ATATATATATTTATTTATTTATATAAAAAAATATTATTTTTTTAATTATTTTTAATTTTAAACATAAAAAAATTAAAAAAATA>9950
10871>------------------------ATATATT----TATTTATATAAAAAAATATTATTTTTTtaattattttTAATTTTAAACATAAAAAAATAAAAAAATA>10942
11122>ATAtatatatatat------ATATATATATTTATTTATTTATATAAAAAAATATTATTTTTTTAATTATTTTTAATTTTAAACATAAAAAAATTAAAAAAATA>11215
10585>ATATATATATatatatatatatatatATATT----TATTTATATAAAAAAATATTATTTTTTTAATTATTTTTAATTTTAAACATAAAAAAATTAAAAAAATA>10680
 8815>ATATAAATTT----------ATTTATATA-----------ATATAAAAAAATATTATTTTTTTAATTATTTTTAATTTTAAACATAAAAAAATTAAAAAAATA>8893

                       *         *         *         *         *         *         *         *         *
 9951>AAAAA--------ATGGGTTGGCAGGCTGGCCCGTTTTGGCCCGCTAAACTGCCAGGTTAAAGTGGGTTGGCGGGCTGAAAATTTCAACCCGGCCCGCCC>10042
10943>AAAAA--------ATGGGTTGGCAGGCTGGCCCGTTTTGGCCCGCTAAACTGCCGGGTTAAAGTGGGTTGGCGGGCTAAAAATTTCAACCCGGCCCGCCC>11034
11216>AAAAA--------ATGGGTTGGCAGGCTGGCCCGTTTTGGCCCGCTAAACTGCCAGGTTAAAGTGGGTTGGCGGGCTGAAAATTTCAACCCGGCCCGCCC>11307
10681>AAAAA--------ATGGGTTGGCAGGCTGGCCCGTTTTGGCCCGCTAAACTGCCAGGTTAAAGTGGGTTGGCGGGCTGAAAATTTCAACCCGGCCCGCCC>10772
 8894>AAAAATAAAAAAAATGGGTTGGCAGGCTGGCCCGTTTTGGCCCGCTAAACTGCCGGGTTAAAGTGGGTTGGCGGGCTGAAAATTTCAACCCGGCCCGCCC>8993

              *         *         *         *         *         *         *         *         *         *
10043>TTTTTTTGGCGGGCTGGCGAGCGGGCTGGACGGGCCTGGTCCATTTTGACATCCCTACGTTTTGGTCTACTCCACATTTAGTTATAAAAATTGCGTCAAA>10142
11035>TTTTTTTGGCGGGCTGGCGAGCGGGCTGGACGGGCCTggtccattttgACATCCCTACGTTTTGGTCTACTCCACATTTAGTTATAAAAATTGCGTCAAA>11134
11308>TTTTTTTGGCGGGCTGGCGAGCGGGCTggacgggcctGGTCCATTTTGACATCCCTACGTTTTGGTCTACTCCATATTTAGTTATAAAAATTGCGTCAAA>11407
10773>TTTTTTTGGCGGGCTGGCGAGCGGGCTGGACGGGCCTGGTCCATTTTGACATCCCTACGTTTTGGTCTACTCCACATTTAGTTATAAAAATTGCGTCAAA>10872
 8994>TTTTTTTGGCGGGCTGGCGAGCGGGCTGGACGGGTCTGGTCCATTTTGACATCCCTACGTTTTGGTCTACTCCACATTTAGTTATAAAAATTGCGTCAAA>9093

              *         *         *         *         *         *         *         *         *         *
10143>TTGACCCGCTTTGCATAAAAAA-TATGGGCTAAATTTTCTAACCTGTTCTGTATAATGTTTGGCCCACGTCTCATGCGAGAAATTATTTATTTTTATATT>10241
11135>TTGACCCGCTTTGCATAAAAAA-TATGGGCTAAATTTTCTAACCTGTTCTGTATAATGTTTGGCCCACGTCTCATGCGAGAAATTATTTATTTTTATATT>11233
11408>TTGACCCGCTTTGCATAAAAAA-TATGGGCTAAATTTTCTAACCTGTTCTGTATAATGTTTGGCCCACGTCTCATGCGAGAAATTATTTATTTTTATATT>11506
10873>TTGACCCGCTTTGCATAAAAAA-TATGGGCTAAATTTTCTAACCTGTTCTGTATAATGTTTGGCCCACGTCTCATGCGAGAAATTATTTATTTTTATATT>10971
 9094>TTGACCCGCTTTGCATAAAAAAATATGTGCTAAATTCTCTAACCTGTCCTCTATAATGTTTGGCCCACGTCTCATGTGAGAAATTATTTATTTTTATATT>9193

               *         *         *         *         *         *         *         *         *         *
10242>TTTTTACCAAGATTATAATTTTAATAACCTTCTTTTTATTTAATTAAATTAGTAAAAATAAAAATTTCTATAAGCCTATTTCTTGAAAAAAAAATAGTCA>10341
11234>TTTTTACCAAGATTATAATTTTAATAACCTTCTTTTTATTTAATTAAATTAGTAAAAATAAAAATTTCTATAAGCCTATTTCTTGAAAAAAAAA-ATTCA>11332
11507>TTTTTACCAAGATTATAATTTTAATAACCTTCTTTTTATTTAATTAAATTAGTAAAAATAAAAATTTCTATAAGCCTATTTCTTGAAAAAAAAATAGTCA>11606
10972>TTTTTACCAAGATTATAATTTTAATAACCTTCTTTTTATTTAATTAAATTAGTAAAAATAAAAATTTCTATAAGCCTATTTCTTGAAAAAAAAATAGTCA>11071
 9194>TTTTTACCAAGATTATAATTTTAATAACCTTCTTTTTATTTAATTAAATTAGTAAAAATAAAAATTTCTATAAGCCTATTTCTTGAAAAAAAAATATTCA>9293

               *         *         *         *         *         *         *         *         *         *
10342>AATTTTATAATTTCTAATTTTGAAATTCAAGTATGTTAAAAATAAAAATGACTTTTGATCTAGATTAGAAAAAAAGAAAAGAAACGCGAACAATAAAGTT>10441
11333>AATTTTATAATTTCTAATTTTGAAATTCAAGTATGTTAAAAATAAAAATGACTTTTGATCTAGATTAGAAAAAAAGGAAAGAAACGCGAACAATAAAGTT>11432
11607>AATTTTATAATTTCTAATTTTGAAATTCAAGTATGTTAAAAATAAAAATGACTTTTGATCTAGATTAGAAAAAAAGAAAAGAAACGCGAACAATAAAGTT>11706
11072>AATTTTATAATTTCTAATTTTGAAATTCAAGTATGTTAAAAATAAAAATGACTTTTGATCTAGATTAGAAAAAAAGAAAAGAAACGCGAACAATAAAGTT>11171
 9294>AATTTTATAATTTCTAATTTTGAAATTCAAGTATGTTAAAAATAAAAATGACTTTTGATCTAGATTAGAAAAAAAGGAAAGAAACGCGAATAATAAAGTT>9393
```

```
               *         *         *         *         *         *         *         *         *         *
10442>GATTTTGGATGGACTAGAGAAAAAAATTTTAATGAGCAACTTACTTATAAAAAACTCACATGATGTATTTTGTTGGTACCATCAAATTTTAAAGATTTAAG>10541
11433>GATTTTGGATGGACTAGAGAAAAAAATTTTAATGAGCAACTTACTTATAAAAAACTCACATGATGTATTTTGTTGGTACCATCAAATTTTAAAGATTTAAG>11532
11707>GATTTTGGATGGACTAGAGAAAAAAATTTTAATGAGCAACTTACTTATAAAAAACTCACATGATGTATTTTGTTGGTACCATCAAATTTTAAAGATTTAAG>11806
11172>GATTTTGGATGGACTAGAGAAAAAAATTTTAATGAGCAACTTACTTATAAAAAACTCACATGATGTATTTTGTTGGTACCATCAAATTTTAAAGATTTAAG>11271
 9394>GATTTTGGATGGACTAGAGAAAAAAATTTTAATGAGCAACTTACTTATAAAAAACTCACATGATGTATTTTGTTGGTACCATCAAATTTTAAAGATTTAAG>9493

               *         *         *         *         *         *         *         *         *         *
10542>TCAATGCGGGTTATGCAGGCTAGTGCAGGCTGACCTGTCGTTGGCTCGCGCGGCTATGAGTTAAGTAGGGCGGGCCTAAGCGAGCCAACAGACTCAAATA>10641
11533>TCAATGCGGGCTATGCAGGCTAGTGCAGGCTGACCTGTCGTTGGCTCGCGCGGCTATGAGTTAAGTAGGGCGGGCCTAAGCGAGCCAACAGACTCAAATA>11632
11807>TCAATGCGGGCTATGCAGGCTAGTGCAGGCTGACCTGTCGTTGGCTCGCGCGGCTATGAGTTAAGTAGGGCGGGCCTAAGCGAGCCAACAGACTCAAATA>11906
11272>TCAATGCGGGCTATGCAGGCTAGTGCAGGCTGACCTGTCGTTGGCTCGCGCGGCTATGAGTTAAGTAGGGCGGGCCTAAGCGAGCCAACAGACTCAAATA>11371
 9494>TCAATGCGGGCTATGCAGGCTAGTGCAGGCTGACCTATCGTTGGCTTGCGCGGCTATGACTTAAGTAGGGCGAGCCTAAGCAAGCCAACAGACTCAAATA>9593

               *         *         *         *         *         *         *         *         *         *
10642>CTTAATTTGTCCCGCACTTGCACTTTTTCACTTTCTCTTGTAGCTTGCAATGTCATCAACATTGTCATTCCTACTTTAAATGGATAGTTGTGAGTAACAA>10741
11633>CTTAATTTGTCCCGCACTTGCACTTTTTCACTTTTTCTTGTAGTTTGCAATGTCATCAACATTGTCATTCCTACTTTAAATGGATAGTTGTGAGTAACAA>11732
11907>CTTAATTTGTCCCGCACTTGCACTTTTTCACTTTCTCTTGTAGCTTGCAATGTCATCAACATTGTCATTCCTACTTTAAATGGATAGTTGTGAGTAACAA>12006
11372>CTTAATTTGTCCCGCACTTGCACTTTTTCACTTTCTCTTGTAGCTTGCAATGTCATCAACATTGTCATTCCTACTTTAAATGGATAGTTGTGAGTAACAA>11471
 9594>CTTAGTTTGTCCCGCACTTGCACTTTTTCACTTTTTCTTGTAGCTTGCAATGTCATCAGCATTGTCATTCCTACTTTAAATGGATAGTCGTGAGTAACAA>9693

               *         *         *         *         *         *         *         *         *         *
10742>ATAACGAACAATATAATGCCTGCTTGTAGATAGGTCAGGTTCAAATATCATTCTATTAGTATCCATGGGTACCCATTACCTGTAAAAAATTAAAATTAAA>10841
11733>ATAACGAACAATATAATGCCTGCTTGTAGATAGGTCAGGTTCAAATATCATTCTATTAGTATCCATGGGTACCCATTACCTGTAAAAAATTAAAATTAAA>11832
12007>ATAACGAACAATATAATGCCTGCTTGTAGATAGGTCAGGTTCAAATATCATTCTATTAGTATCCATGGGTACCCATTACCTGTAAAAAATTAAAATTAAA>12106
11472>ATAACGAACAATATAATGCCTGCTTGTAGATAGGTCAGGTTCAAATATCATTCTATTAGTATCCATGGGTACCCATTACCTGTAAAAAATTAAAATTAAA>11571
 9694>ATAACGAATAATATAATGCCTGCTTGTAGATAGGTCAGGTTCAA-TATCATTCTACTATTATCCATGGATACCCATTACCTGTAAAAAATTAAAATTAAA>9792

               *         *         *         *         *                   *         *         *
10842>AGTTAATTTATATTTTATTAGATAAATTTAATTAAAATCAAAATTTTCA------------TTTAATTTTATATACATTATAGTTTATATTTATTTGTAA>10929
11833>AGTTAATTTATATTTTATTAGATAAATTTAATTAAAATCAAAATTTTCA------------TTTAATTTTATATACATTATAGTTTATATTTATTTGTAA>11920
12107>AGTTAATTTATATTTTATTAGATAAATTTAATTAAAATCAAAATTTTCA------------TTTAATTTTATATACATTATAGTTTATATTTATTTGTAA>12194
11572>AGTTAATTTATATTTTATTAGATAAATTTAATTAAAATCAAAATTTTCA------------TTTAATTTTATATACATTATAGTTTATATTTATTTGTAA>11659
 9793>ATTTAATTTATATTTTATTAAATAAATTTAATTAAAATCAAAATTTTCATTTTCATTTTCATTTAATTTTATATACATTATAGTTTATATTTTTTTGTAA>9892

      *         *         *         *         *         *         *         *         *         *
10930>TTTTATTTTAGATTTTATTTTAAAAATCTATGAATTATTATTTTTCAAATATTTGTGGTCATGCATGGATATTCATGTATTCTAAAAAATTCATAAACAT>11029
11921>TTTTATTTTAGATTTTATTTTAAAAATCTATGAATTATTATTTTTCAAATATTTGTGATCATGCATGGATATTCATGTATTCTAAAAAATTCATAAACAT>12020
12195>TTTTATTTTAGATTTTATTTTAAAAATCTATGAATTATTATTTTTCAAATATTTGTGGTCATGCATGGATATTCATGTATTCTAAAAAATTCATAAACAT>12294
11660>TTTTATTTTAGATTTTATTTTAAAAATCTATGAATTATTATTTTTCAAATATTTGTGGTCATGCATGGATATTCATGTATTCTAAAAAATTCATAAACAT>11759
 9893>TTTTATTTTAGATTTTATTTTAAAAATCTATGAATTATTATTTTTCAAATATTTGTGGTCATGCATGGATATTCATGTATTCTAAAAAATCCATAAA-AT>9991

      *         *         *         *         *         *         *         *         *         *
11030>TTTTTAAACGAATATTTGGCGATTAGCAAAGTAAATAATATGTCAAATTTTTGTTACAAGTCGAATAACGAGTAATCACTATTCATTATCATTATCCCTA>11129
12021>TTTTTAAACGAATATTTGGCGATTAGCAAAGTAAATAATATGTCAAATTTTTGTTA--AGTCGAATAACGAGTAATCACTATTCATTATCATTATCCCTA>12118
12295>TTTTTAAACGAATATTTGGCGATTAGCAAAGTAAATAATATGTCAAATTTTTGTTACAAGTCGAATAACGAGTAATCACTATTCATTATCATTATCCCTA>12394
11760>TTTTTAAACGAATATTTGGCGATTAGCAAAGTAAATAATATGTCAAATTTTTGTTACAAGTCGAATAACGAGTAATCACTATTCATTATCATTATCCCTA>11859
 9992>TTTTTAAACAAATATTTGACAATTAACAAAGTAAATAATATGTCAAATTTTTGTTACAAGTCGAATAACGAGTAAGCACTATTCATTACCATTATCCCTA>10091

      *         *         *         *         *         *         *         *         *         *
11130>CACAATCACAAACCTCTTGTCATCCCTACACAATCACAAGGTCATAATATAATTGTTAACTTTCAAAGTTTGGGTCTAGTATTCAAATAAACGTTAACAA>11229
12119>CACAATCACAAACCTCTTGTCATCCCTACACAATCACAAGGTCATAATATAATTGTTAACTTTCAAAGTTTGGGTCTAGTATTCAAATAAACGTTAACAA>12218
12395>CACAATCACAAACCTCTTGTCATCCCTACACAATCACAAGGTCATAATATAATTGTTAACTTTCAAAGTTTGGGTCTAGTATTCAAATAAACGTTAACAA>12494
11860>CACAATCACAAACCTCTTGTCATCCCTACACAATCACAAGGTCATAATATAATTGTTAACTTTCAAAGTTTGGGTCTAGTATTCAAATAAACGTTAACAA>11959
10092>CACAATCACAAACCTCTTGTCATCCCTACACAATCACAAGGTCATAATATAATTGTTAACTTTCAAAGTTTGGGTCTAGTATTCAAATAAACGTTAACAA>10191

      *         *         *         *         *         *         *         *         *         *
11230>TAAATTAACTTTACGTATATTAAATATGTTAAAAATACGTTAGAGAAAAAATTATAATAATAGTTAAAATGTTTAAGTACACACATTAAAGGAAGTAATT>11329
12219>TAAATTAACTTTACGTATATTAAATATGTTAAAAATACGTTAGAGAAAAAATTATAATAATAGTTAAAATGTTTAAGTACACACATTAAAGGAAGTAATT>12318
12495>TAAATTAACTTTACGTATATTAAATATGTTAAAAATACGTTAGAGAAAAAATTATAATAATAGTTAAAATGTTTAAGTACACACATTAAAGGAAGTAATT>12594
11960>TAAATTAACTTTACGTATATTAAATATGTTAAAAATACGTTAGAGAAAAAATTATAATAATAGTTAAAATGTTTAAGTACACACATTAAAGGAAGTAATT>12059
10192>TAAATTAACTTTACGTATATTAAATATGTTAAAAATACGTTAGAGAAAAAATTATAATAATAGTTAAAATGTTTAAGTACACACATTAAAGGAAGTAATT>10291

      *         *         *         *         *         *         *         *         *         *
11330>ATTTATTTATTTTGAAAATTAACTAACTTTTAAAATAATTTATAAGAATACATATCATGGATTCAATTGATTAACTAAAACAAATTCAAACTTTATATAT>11429
12319>ATTTATTTATTTTGAAAATTAACTAACTTTTAAAATAATTTATAAGAATACATATCATGGATTCAATTGATTAACTAAAACAAATTCAAACTTTATATAT>12418
12595>ATTTATTTATTTTGAAAATTAACTAACTTTTAAAATAATTTATAAGAATACATATCATGGATTCAATTGATTAACTAAAACAAATTCAAACTTTATATAT>12694
12060>ATTTATTTATTTTGAAAATTAACTAACTTTTAAAATAATTTATAAGAATACATATCATGGATTCAATTGATTAACTAAAACAAATTCAAACTTTATATAT>12159
10292>ATTTATTTATTTTGAAAATTAACTAACTTTTAAAATAATTTATAAGAATACATATCATGGATTCAATTGATTAACTAAAACAAATTCAAACTTTATATAT>10391
```

```
      *         *         *         *         *         *         *         *         *         *
11430>AATTGACGTTTTAGGGTTTGATATAAAGATTTTAAATTTGATAAACTATTGTGTCATTTACGAACTAAAATAATAATTTATTAAAAATTAATCGGTAGTG>11529
12419>AATTGACGTTTTAGGGTTTGATATAAAGATTTTAAATTTGATAAACTATTGTGTCATTTACGAACTAAAATAATAATTTATTAAAAATTAATCGGTAGTG>12518
12695>AATTGACGTTTTAGGGTTTGATATAAAGATTTTAAATTTGATAAACTATTGTGTCATTTACGAACTAAAATAATAATTTATTAAAAATTAATCGGTAGTG>12794
12160>AATTGACGTTTTAGGGTTTGATATAAAGATTTTAAATTTGATAAACTATTGTGTCATTTACGAACTAAAATAATAATTTATTAAAAATTAATCGGTAGTG>12259
10392>AATTGACGTTTTAGGGTTTGATATAAAGATTTTAAATTTGATAAACTATTGTGTCATTTACGAACTAAAATAATAATTTATTAAAAATTAATCGGTAGTG>10491

      *         *         *         *         *         *         *         *         *         *
11530>TTTGAACTATTTTTATTTTTACGGAAAATCAGTTTAAAAACAAAAACAAATGAATAAAATAATAAAATAATTCTTTAGAGAAAAATCTTTTATATGATAA>11629
12519>TTTGAACTATTTTTATTTTTACGGAAAATCAGTTTAAAAACAAAAACAAATGAATAAAATAATAAAATAATTCTTTAGAGAAAAATCTTTTATATGATAA>12618
12795>TTTGAACTATTTTTATTTTTACGGAAAATCAGTTTAAAAACAAAAACAAATGAATAAAATAATAAAATAATTCTTTAGAGAAAAATCTTTTATATGATAA>12894
12260>TTTGAACTATTTTTATTTTTACGGAAAATCAGTTTAAAAACAAAAACAAATGAATAAAATAATAAAATAATTCTTTAGAGAAAAATCTTTTATATGATAA>12359
10492>TTGGAACTATTTTTATTTTTACGGAAAATCAGTTTAAAAACAAAAACAAATGAATAAAATAATAAAATAATTCTTTAGAGAAAAATCTTTTATATGACAA>10591

      *         *         *         *         *         *         *         *         *         *
11630>TACAAACAGTTTGTATTATTAAAAAATATATAATTTTTTAAGTAATATTATTTAAGAAACGAAAGTAAAATATATTTTTACATATTTAATTTTATTAAAT>11729
12619>TACAAACAGTTTGTATTATTAAAAAATATATAATTTTTTAAGTAATATTATTTAAGAAACGAAAGTAAAATATATTTTTACATATTTAATTTTATTAAAT>12718
12895>TACAAACAGTTTGTATTATTAAAAAATATATAATTTTTTAAGTAATATTATTTAAGAAACGAAAGTAAAATATATTTTTACATATTTAATTTTATTAAAT>12994
12360>TACAAACAGTTTGTATTATTAAAAAATATATAATTTTTTAAGTAATATTATTTAAGAAACGAAAGTAAAATATATTTTTACATATTTAATTTTATTAAAT>12459
10592>TACAAACAGTTTGTATTATTAAAAAATATATA-TTTTTTAAGTAATATTATTTAAGAAACGAAAGTAAAATATATTTTTACATATTTAATTTTATTAAAT>10690

      *         *         *         *         *         *         *         *         *         *
11730>GACCAAATAATTAAAAATCATAAAAAGATAAAATAAAAAAATGAAAAATGTAGCATAAAAAAGTAAGTATTACTTTAAATATGAGTTATTCTTTAATATT>11829
12719>GACCAAATAATTAAAAATCATAAAAAGATAAAATAAAAAAATGAAAAATGTAGCATAAAAAAGTAAGTATTACTTTAAATATGAGTTATTCTTTAATATT>12818
12995>GACCAAATAATTAAAAATCATAAAAAGATAAAATAAAAAAATGAAAAATGTAGCATAAAAAAGTAAGTATTACTTTAAATATGAGTTATTCTTTAATATT>13094
12460>GACCAAATAATTAAAAATCATAAAAAGATAAAATAAAAAAATGAAAAATGTAGCATAAAAAAGTAAGTATTACTTTAAATATGAGTTATTCTTTAATATT>12559
10691>GACCAAATAATTAAAAATCATAAAAAGATAAAATAAAAAAATGAAAAATGTAGCATAAAAAAGTAAGTATTACTTTAAATATGAGTTATTCTTTAATCTT>10790

      *         *         *         *         *         *         *         *         *         *
11830>TCATTAAGACCAAGTAATTAAAAATTATAATAGGCTAAAATTAAGAATGAAAAAAATAGTATAGAAAAATATTTTCATTTTTAGGACAAAATATATATAA>11929
12819>TCATTAAGACCAAGTAATTAAAAATTATAATAGGCTAAAATTAAGAATGAAAAAAATAGTATAGAAAAATATTTTCATTTTTAGGACAAAATATATATAA>12918
13095>TCATTAAGACCAAGTAATTAAA~~~~~~~~~~~~~~~~~~~~~~~~~~~~~~~~~~~~~~~~~~~~~~~~~~~~~~~~~~~~~~~~~~~~~~~~~~~~~~>13116
12560>TCATTAAGACCAAGTAATTAAAAATTATAATAGGCTAAAATTAAGAATGAAAAAAATAGTATAGAAAAATATTTTCATTTTTAGGACAAAATATATATAA>12659
10791>TCATTAAGACCAAGTAATTAAAAATTATAATAGGCTAAAATTAAGAATGAAAAAAATAGTATAGAAAAATATTTTCATTTTTAGGAAAAAATATATATAA>10890

      *         *         *         *         *         *         *         *         *         *
11930>ATAAAAATAAATAAATTAAAAATGTCAAATGACTAAAAATTTAACTTTTGAGACACCTATCAACAAAATAAACATAAATAATAAAATAAATTTAATAGAT>12029
12919>ATAAAAATAAATAAATTAAAAATGTCAAATGACTAAAAATTTAACTTTTGAGACATCTATCAACAAAATAAACATAAATAATAAAATAAATTTAATAGAT>13018
13116>~~~~~~~~~~~~~~~~~~~~~~~~~~~~~~~~~~~~~~~~~~~~~~~~~~~~~~~~~~~~~~~~~~~~~~~~~~~~~~~~~~~~~~~~~~~~~~~~~~~~>13116
12660>ATAAAAATAAATAAATTAAAAATGTCAAATGACTAAAAATTTAACTTTTGAGACACCTATC~~~~~~~~~~~~~~~~~~~~~~~~~~~~~~~~~~~~~~~>12720
10891>ATAAAAATAAATAAATTAAAAATGTCAAATGACTAAAAATTTAACTTTTGAGACACCTATCAACAAAATAAACATAAATAATAAAATAAATTTAATAGAT>10990

      *         *         *         *         *         *         *         *         *         *
12030>AATATATATTAAACATTTGAACTATTAAATACAATTTTTATATAGAATTCTATGTACATATGGTTACTATAGTTATTTATTAGAAAATATTAATTTAAAT>12129
13019>AATATATATTAAACATTTGAACTATTAAATACAATTTTTATATAGAATTCTATGTACATATGGTTACTATAGTTATTTATTAGAAAATATTAATTTAAAT>13118
13116>~~~~~~~~~~~~~~~~~~~~~~~~~~~~~~~~~~~~~~~~~~~~~~~~~~~~~~~~~~~~~~~~~~~~~~~~~~~~~~~~~~~~~~~~~~~~~~~~~~~~>13116
12720>~~~~~~~~~~~~~~~~~~~~~~~~~~~~~~~~~~~~~~~~~~~~~~~~~~~~~~~~~~~~~~~~~~~~~~~~~~~~~~~~~~~~~~~~~~~~~~~~~~~~>12720
10991>AATATATATTAAACATTTGAACTATTAAATACAATTTTTATATAGAATTCTATGTACATATGGTTACTATAGTTATTTATTAGAAAATATTAATTTAAAT>11090

      *         *         *         *         *         *         *         *         *         *
12130>TTGTATTTATATTATAATTTAATAAAAATATGTTTTATTTTTTATAAGATTTTTAAGATAAAATTATTATTACTATTTTTAAATAATATTATTTTACAAA>12229
13119>TTGTATTTATATTATAATTTAATAAAAATATGTTTTATTTTTTATAAGATTTTTAAGATAAAATTATTATTACTATTTTTAAATAATATTATTTTACAAA>13218
13116>~~~~~~~~~~~~~~~~~~~~~~~~~~~~~~~~~~~~~~~~~~~~~~~~~~~~~~~~~~~~~~~~~~~~~~~~~~~~~~~~~~~~~~~~~~~~~~~~~~~~>13116
12720>~~~~~~~~~~~~~~~~~~~~~~~~~~~~~~~~~~~~~~~~~~~~~~~~~~~~~~~~~~~~~~~~~~~~~~~~~~~~~~~~~~~~~~~~~~~~~~~~~~~~>12720
11091>TTGTATTTATATTATAATTTAATAAAAATATGTTTTATTTTTTATAAGATTTTTAAGATAAAATTATTATTACTATTTTTAAATAATATTATTTTACAAA>11190

      *         *         *         *         *         *         *         *         *         *
12230>ATAAAATTAAAACATAG~~~~~~~~~~~~~~~~~~~~~~~~~~~~~~~~~~~~~~~~~~~~~~~~~~~~~~~~~~~~~~~~~~~~~~~~~~~~~~~~~~~>12246
13219>ATAAAATTAAAACATAGTTTTGAAATTTAATTTTATTAAATGACTCTAAGTAGTGAAATATATAAAAGAAAAAAAAAGTAGTATAGAAAAAATAATTAAT>13318
13116>~~~~~~~~~~~~~~~~~~~~~~~~~~~~~~~~~~~~~~~~~~~~~~~~~~~~~~~~~~~~~~~~~~~~~~~~~~~~~~~~~~~~~~~~~~~~~~~~~~~~>13116
12720>~~~~~~~~~~~~~~~~~~~~~~~~~~~~~~~~~~~~~~~~~~~~~~~~~~~~~~~~~~~~~~~~~~~~~~~~~~~~~~~~~~~~~~~~~~~~~~~~~~~~>12720
11191>ATAAAATTAAAACATAGTTTTGAAATTTAATTTTATTAAATGACTCTAAGTAGTGAAATATATAAAAGAAAA~~~~~~~~~~~~~~~~~~~~~~~~~~~~>11262

      *
12246>~~~~~~>12246
13319>TTATTT>13324
13116>~~~~~~>13116
12720>~~~~~~>12720
11262>~~~~~~>11262
```
