# Supplementary material for: Seed Coat Pattern QTL and Development in Cowpea (Vigna unguiculata [L.] Walp.)
Source: Front Plant Sci. 2019 Oct 25;10:1346. doi: 10.3389/fpls.2019.01346 (PMC6824211; doi:10.3389/fpls.2019.01346)
Supplement: Supplementary file 3 [file DataSheet_2.pdf]

```
Tue Mar 12, 2019 16:40 PDT
Cowpea1.0_Vu09(old8)_30205000_30209613.fa from 1 to 4614
Alignment to
CowpeaA_LG9_27471300-27475947.fa--  Matches:4614; Mismatches:0; Gaps:34; Unattempted:0
CowpeaB_LG9_13109199-13113800.fa--  Matches:4600; Mismatches:2; Gaps:12; Unattempted:0
CowpeaD_LG9_25306000-25310538.fa--  Matches:4537; Mismatches:2; Gaps:75; Unattempted:0
CowpeaC_LG9_24769700-24774326.fa--  Matches:4611; Mismatches:3; Gaps:13; Unattempted:0

             *         *         *         *         *         *         *         *         *         *
   1>~~~~~~~~~~~~~~~~~~~~~~~~~~~~~~~~~~CCAATGGAAAACCAGATAAAAAGATATCCAACATATAAGAGTAGAGTAATGAAGCATAAGGCAATA>66
   1>ACACAACCCCTACAAAACTAGGCACACCACGATGCCAATGGAAAACCAGATAAAAAGATATCCAACATATAAGAGTAGAGTAATGAAGCATAAGGCAATA>100
   1>~~~~~~~~~~~~~~~~~~~~~~~~~~~~~~~~~~C------------AGATAAAAAGATATCCAACATATAAGAGTAGAGTAATGAAGCATAAGGCAATA>54
   0>~~~~~~~~~~~~~~~~~~~~~~~~~~~~~~~~~~~~~~~~~~~~~~~~~~~~~~~~~~~~~~~~~~~~~~~~~~~~~~~~~~~~~~~~~~~~~~~~~~~~>0
   1>GCACACCACGATG---------------------CCAATGGAAAACCAGATAAAAAGATATCCAACATATAAGAGTAGAGTAATGAAGCATAAGGCAATA>79

             *         *         *         *         *         *         *         *         *         *
  67>CATGGGTCATAACCCTTAAGAAAACCCATTAAGAAACAAAGTCCAGCCCATGGAGACTATGCGGTGGAACCATCACCTCCTTGTTGCCCACGGGTGTTTC>166
 101>CATGGGTCATAACCCTTAAGAAAACCCATTAAGAAACAAAGTCCAGCCCATGGAGACTATGCGGTGGAACCATCACCTCCTTGTTGCCCACGGGTGTTTC>200
  55>CATGGGTCATAACCCTTAAGAAAACCCATTAAGAAACAAAGTCCAGCCCATGGAGACTATGCGGTGGAACCATCACCTCCTTGTTGCCCACGGGTGTTTC>154
   1>~~~~~~~~~TAACCCTTAAGAAAACCCATTAAGAAACAAAGTCCAGCCCATGGAGACTATGCGGTGGAACCATCACCTCCTTGTTGCCCACGGGTGTTTC>91
  80>CATGGGTCATAACCCTTAAGAAAACCCATTAAGAAACAAAGTCCAGCCCATGGAGACTATGCGGTGGAACCATCACCTCCTTGTTGCCCACGGGTGTTTC>179

             *         *         *         *         *         *         *         *         *         *
 167>TCGCATATGCTCACATCAATAAGTTGATGATCATCGCAAAAAAGAGTACCACACAACAAAACACACAACAATAAAAGTAGGGTAAGCTAGAGCACAAAAG>266
 201>TCGCATATGCTCACATCAATAAGTTgatgatcatcGCAAAAAAGAGTACCACACAACAAAACACACAACAATAAAAGTAGGGTAAGCTAGAGCACAAAAG>300
 155>TCGCATATGCTCACATCAATAAGTTGATGATCATCGCAAAAAAGAGTACCACACAACAAAACACACAACAATAAAAGTAGGGTAAGCTAGAGCACAAAAG>254
  92>TCGCATATGCTCACATCAATAAGTTGATGATCATCGCAAAAAAGAGTACCACACAACAAAACACACAACAATAAAAGTAGGGTAAGCTAGAGCACAAAAG>191
 180>TCGCATATGCTCACATCAATAAGTTGATGATCATCGCAAAAAAGAGTACCACACAACAAAACACACAACAATAAAAGTAGGGTAAGCTAGAGCACAAAAG>279

             *         *         *         *         *         *         *         *         *         *
 267>ATTTCATCCATCAAATCATATATAATTTCACAATATCATACACTCAAATCAACCATGCATGTTATGACTTTTCAGGCAATACACTACAACACGACTCGAC>366
 301>ATTTCATCCATCAAATCATATATAATTTCACAATATCATACACTCAAATCAACCATGCATGTTATGACTTTTCAGGCAATACACTACAACACGACTCGAC>400
 255>ATTTCATCCATCAAATCATATATAATTTCACAATATCATACACTCAAATCAACCATGCATGTTATGACTTTTCAGGCAATACACTACAACACGACTCGAC>354
 192>ATTTCATCCATCAAATCATATATAATTTCACAATATCATACACTCAAATCAACCATGCATGTTATGACTTTTCAGGCAATACACTACAACACGACTCGAC>291
 280>ATTTCATCCATCAAATCATATATAATTTCACAATATCATACACTCAAATCAACCATGCATGTTATGACTTTTCAGGCAATACACTACAACACGACTCGAC>379

             *         *         *         *         *         *         *         *         *         *
 367>TCATCCGGATACGTATAACCTGGTCGAATTTAGCGGATGCTTGCACTTGTGGTGGATACCTCTACTCACCCCCGAGCTGCTCACCCCCGAGCTATGTGTT>466
 401>TCATCCGGATACGTATAACCTGGTCGAATTTAGCGGATGCTTGCACTTGTGGTGGATACCTCTACTCACCCCCGAGCTGCTCACCCCCGAGCTATGTGTT>500
 355>TCATCCGGATACGTATAACCTGGTCGAATTTAGCGGATGCTTGCACTTGTGGTGGATACCTCTACTCACCCCCGAGCTGCTCACCCCCGAGCTATGTGTT>454
 292>TCATCCGGATACGTATAACCTGGTCGAATTTAGCGGATGCTTGCACTTGTGGTGGATACCTCTACTCACCCCCGAGCTGCTCACCCCCGAGCTATGTGTT>391
 380>TCATCCGGATACGTATAACCTGGTCGAATTTAGCGGATGCTTGCACTTGTGGTGGATACCTCTACTCACCCCCGAGCTGCTCACCCCCGAGCTATGTGTT>479

             *         *         *         *         *         *         *         *         *         *
 467>GCAAGTGTTACAATGAATCAATCCCACACACACAAAGTTAGCCCTTAATGAGTTTCGGGCCTCCTGCTACTCTCACCACAAGAGTCAGTCCGCTCTAAGT>566
 501>GCAAGTGTTACAATGAATCAATCCCACACACACAAAGTTAGCCCTTAATGAGTTTCGGGCCTCCTGCTACTCTCACCACAAGAGTCAGTCCGCTCTAAGT>600
 455>GCAAGTGTTACAATGAATCAATCCCACACACACAAAGTTAGCCCTTAATGAGTTTCGGGCCTCCTGCTACTCTCACCACAAGAGTCAGTCCGCTCTAAGT>554
 392>GCAAGTGTTACAATGAATCAATCCCACACACACAAAGTTAGCCCTTAATGAGTTTCGGGCCTCCTGCTACTCTCACCACAAGAGTCAGTCCGCTCTAAGT>491
 480>GCAAGTGTTACAATGAATCAATCCCACACACACAAAGTTAGCCCTTAATGAGTTTCGGGCCTCCTGCTACTCTCACCACAAGAGTCAGTCCGCTCTAAGT>579

             *         *         *         *         *         *         *         *         *         *
 567>GAGACTAACTGACTCCTTAGAGTCTCAGGATGCAATCCTTACCTTGAATCCTTACCAAATTATATAGATGGGGCACCACCATGGACACCCACTAACAGGG>666
 601>GAGACTAACTGACTCCTTAGAGTctcaggatgcaATCCTTACCTTGAATCCTTACCAAATTATATAGATGGGGCACCACCATGGACACCCACTAACAGGG>700
 555>GAGACTAACTGACTCCTTAGAGTctcaggatgcaATCCTTACCTTGAATCCTTACCAAATTATATAGATGGGGCACCACCATGGACACCCACTAACAGGG>654
 492>GAGACTAACTGACTCCTTAGAGTctcaggatgcaATCCTTACCTTGAATCCTTACCAAATTATATAGATGGGGCACCACCATGGACACCCACTAACAGGG>591
 580>GAGACTAACTGACTCCTTAGAGTctcaggatgcaATCCTTACCTTGAATCCTTACCAAATTATATAGATGGGGCACCACCATGGACACCCACTAACAGGG>679

             *         *         *         *         *         *         *         *         *         *
 667>ACCATGAAATTACGTCCCGACCATTGAAGCACGACCTTGAAATCTCACATAGAGATTCCAAGGAATTACGTACTGACCACATACTATACATATTCAAACA>766
 701>ACCATGAAATTACGTCCCGACCATTGAAGCACGACCTTGAAATCTCACATAGAGATTCCAAGGAATTACGTACTGACCACATACTATACATATTCAAACA>800
 655>ACCATGAAATTACGTCCCGACCATTGAAGCACGACCTTGAAATCTCACATAGAGATTCCAAGGAATTACGTACTGACCACATACTATACATATTCAAACA>754
 592>ACCATGAAATTACGTCCCGACCATTGAAGCACGACCTTGAAATCTCACATAGAGATTCCAAGGAATTACGTACTGACCACATACTATACATATTCAAACA>691
 680>ACCATGAAATTACGTCCCGACCATTGAAGCACGACCTTGAAATCTCACATAGAGATTCCAAGGAATTACGTACTGACCACATACTATACATATTCAAACA>779

             *         *         *         *         *         *         *         *         *         *
 767>AACAATAATATTTATTATGCTTATAAATATCATGCCAACCTTTATAATCCATCCTCATACATGTTCCATGTTGAATCCATTCCAAATCATCCTTCCCAAT>866
 801>AACAATAATATTTATTATGCTTATAAATATCATGCCAACCTTTATAATCCATCCTCATACATGTTCCATGTTGAATCCATTCCAAATCATCCTTCCCAAT>900
 755>AACAATAATATTTATTATGCTTATAAATATCATGCCAACCTTTATAATCCATCCTCATACATGTTCCATGTTGAATCCATTCCAAATCATCCTTCCCAAT>854
 692>AACAATAATATTTATTATGCTTATAAATATCATGCCAACCTTTATAATCCATCCTCATACATGTTCCATGTTGAATCCATTCCAAATCATCCTTCCCAAT>791
 780>AACAATAATATTTATTATGCTTATAAATATCATGCCAACCTTTATAATCCATCCTCATACATGTTCCATGTTGAATCCATTCCAAATCATCCTTCCCAAT>879
```

```
               *         *         *         *         *         *         *         *         *         *
 867>TCATGAATATAGAAGTGTAATTCATACGGACACCATGCCACTTTGATTACCAACCATCTCATACAAGTCACATGCCAAGGTCATGTTAAACTTATCAATC>966
 901>TCATGAATATAGAAGTGTAATTCATACGGACACCATGCCACTTTGATTACCAACCATCTCATACAAGTCACATGCCAAGGTCATGTTAAACTTATCAATC>1000
 855>TCATGAATATAGAAGTGTAATTCATACGGACACCATGCCACTTTGATTACCAACCATCTCATACAAGTCACATGCCAAGGTCATGTTAAACTTATCAATC>954
 792>TCATGAATATAGAAGTGTAATTCATACGGACACCATGCCACTTTGATTACCAACCATCTCATACAAGTCACATGCCAAGGTCATGTTAAACTTATCAATC>891
 880>TCATGAATATAGAAGTGTAATTCATACGGACACCATGCCACTTTGATTACCAACCATCTCATACAAGTCACATGCCAAGGTCATGTTAAACTTATCAATC>979

               *         *         *         *         *         *         *         *         *         *
 967>ACAGACCATAAACCATTCTACTCATACCCATTCGCCCAATTCATGCTTTTAAAAGTAATTGAATCAATCCACAAGTTCAAGTTAAAGTAAGGAATTAAAA>1066
1001>ACAGACCATAAACCATTCTACTCATACCCATTCgcccaattcaTGCTTTTAAAAGTAATTGAATCAATCCACAAGTTCAAGTTAAAGTAAGGAATTAAAA>1100
 955>ACAGACCATAAACCATTCTACTCATACCCATTCgcccaattcaTGCTTTTAAAAGTAATTGAATCAATCCACAAGTTCAAGTTAAAGTAAGGAATTAAAA>1054
 892>ACAGACCATAAACCATTCTACTCATACCCATTCgcccaattcaTGCTTTTAAAAGTAATTGAATCAATCCACAAGTTCAAGTTAAAGTAAGGAATTAAAA>991
 980>ACAGACCATAAACCATTCTACTCATACCCATTCgcccaattcaTGCTTTTAAAAGTAATTGAATCAATCCACAAGTTCAAGTTAAAGTAAGGAATTAAAA>1079

               *         *         *         *         *         *         *         *         *         *
1067>TTCTGCAGCAATTATCGCTTAAGCTAGACTGATCTCGCTTAAGCTAGAAATTCTCGCTTAAGCGACTTAAGCAAGACTGATCTCGCTTAAGCTAAAATCC>1166
1101>TTCTGCAGCAATTATCGCTTAAGCTAGACTGATCTCGCTTAAGCTAGAAATTCTCGCTTAAGCGACTTAAGCAAGACTGATCTCGCTTAAGCTAAAATCC>1200
1055>TTCTGCAGCAATTATCGCTTAAGCTAGACTGATCTCGCTTAAGCTAGAAATTCTCGCTTAAGCGACTTAAGCAAGACTGATCTCGCTTAAGCTAAAATCC>1154
 992>TTCTGCAGCAATTATCGCTTAAGCTAGACTGATCTCGCTTAAGCTAGAAATTCTCGCTTAAGCGACTTAAGCAAGACTGATCTCGCTTAAGCTAAAATCC>1091
1080>TTCTGCAGCAATTATCGCTTAAGCTAGACTGATCTCGCTTAAGCTAGAAATTCTCGCTTAAGCGACTTAAGCAAGACTGATCTCGCTTAAGCTAAAATCC>1179

               *         *         *         *         *         *         *         *         *         *
1167>CTTGCTTAAGCTAAACTGACATCTTTTTAATGATTTCAACATGTAAAAACACAAAATCCCAAACAACAAAAATTTAAGAAAACACAAGCATGTTGTATAA>1266
1201>CTTGCTTAAGCTAAACTGACATCTTTTTAATGATTTCAACATGTAAAAACACAAAATCCCAAACAACAAAAATTTAAGAAAACACAAGCATGTTGTATAA>1300
1155>CTTGCTTAAGCTAAACTGACATCTTTTTAATGATTTCAACATGTAAAAACACAAAATCCCAAACAACAAAAATTTAAGAAAACACAAGCATGTTGTATAA>1254
1092>CTTGCTTAAGCTAAACTGACATCTTTTTAATGATTTCAACATGTAAAAACACAAAATCCCAAACAACAAAAATTTAAGAAAACACAAGCATGTTGTATAA>1191
1180>CTTGCTTAAGCTAAACTGACATCTTTTTAATGATTTCAACATGTAAAAACACAAAATCCCAAACAACAAAAATTTAAGAAAACACAAGCATGTTGTATAA>1279

               *         *         *         *         *         *         *         *         *         *
1267>AACGTTGGCATCATATTTCATGCTTTAAAAGAGTAAACAAATCAATCATGGGAGCATACAAACATTTAACAACTGTCAATCTCGCTCAAGCTAAGGAGCT>1366
1301>AACGTTGGCATCATATTTCATGCTTTAAAAGAGTAAACAAATCAATCATGGGAGCATACAAACATTTAACAACTGTCAATCTCGCTCAAGCTAAGGAGCT>1400
1255>AACGTTGGCATCATATTTCATGCTTTAAAAGAGTAAACAAATCAATCATGGGAGCATACAAACATTTAACAACTGTCAATCTCGCTCAAGCTAAGGAGCT>1354
1192>AACGTTGGCATCATATTTCATGCTTTAAAAGAGTAAACAAATCAATCATGGGAGCATACAAACATTTAACAACTGTCAATCTCGCTCAAGCTAAGGAGCT>1291
1280>AACGTTGGCATCATATTTCATGCTTTAAAAGAGTAAACAAATCAATCATGGGAGCATACAAACATTTAACAACTGTCAATCTCGCTCAAGCTAAGGAGCT>1379

               *         *         *         *         *         *         *         *         *         *
1367>CTCGCTCAGGCAACAGGGTCTCTCGCTCAAGCGAAAAACTTTCAGTTAAGCGAGATTGAAAACAGAGAGTACTTCGAGTTCTCGCTCAAGCCAGCCCATC>1466
1401>CTCGCTCAGGCAACAGGGTCTCTCGCTCAAGCGAAAAACTTTCAGTTAAGCGAGATTGAAAACAGAGAGTACTTCGAGTTCTCGCTCAAGCCAGCCCATC>1500
1355>CTCGCTCAGGCAACAGGGTCTCTCGCTCAAGCGAAAAACTTTCAGTTAAGCGAGATTGAAAACAGAGAGTACTTCGAGTTCTCGCTCAAGCCAGCCCATC>1454
1292>CTCGCTCAGGCAACAGGGTCTCTCGCTCAAGCGAAAAACTTTCAGTTAAGCGAGATTGAAAACAGAGAGTACTTCGAGTTCTCGCTCAAGCCAGCCCATC>1391
1380>CTCGCTCAGGCAACAGGGTCTCTCGCTCAAGCGAAAAACTTTCAGTTAAGCGAGATTGAAAACAGAGAGTACTTCGAGTTCTCGCTCAAGCCAGCCCATC>1479

               *         *         *         *         *         *         *         *         *         *
1467>TCACTCAAGCGAGAGGCCTTCGCTCAGGCGAGCACTCGAAACTAAAAGGGGGCGCTTAGGCGAGAGCTCATCGCTTAGGCGAGAGCTTTTCGTTTGGGCG>1566
1501>TCACTCAAGCGAGAGGCCTTCGCTCAGGCGAGCACTCGAAACTAAAAGGGGGCGCTTAGGCGAGAGCTCATCGCTTAGGCGAGAGCTTTTCGTTTGGGCG>1600
1455>TCACTCAAGCGAGAGGCCTTCGCTCAGGCGAGCACTCGAAACTAAAAGGGGGCGCTTAGGCGAGAGCTCATCGCTTAGGCGAGAGCTTTTCGTTTGGGCG>1554
1392>TCACTCAAGCGAGAGGCCTTCGCTCAGGCGAGCACTCGAAACTAAAAGGGGGCGCTTAGGCGAGAGCTCATCGCTTAGGCGAGAGCTTTTCGTTTGGGCG>1491
1480>TCACTCAAGCGAGAGGCCTTCGCTCAGGCGAGCACTCGAAACTAAAAGGGGGCGCTTAGGCGAGAGCTCATCGCTTAGGCGAGAGCTTTTCGTTTGGGCG>1579

               *         *         *         *         *         *         *         *         *         *
1567>AGAAACAGCTCGCTTAGGCGAGCATAGTAGATCTCACCTGCTCTCACGCATGCAAAGCCAAAAATCCATCCAGAAACAATACACCGATTATTTTCACACC>1666
1601>AGAAACAGCTCGCTTAGGCGAGCATAGTAGATCTCACCTGCTCTCACGCATGCAAAGCCAAAAATCCATCCAGAAACAATACACCGATTATTTTCACACC>1700
1555>AGAAACAGCTCGCTTAGGCGAGCATAGTAGATCTCACCTGCTCTCACGCATGCAAAGCCAAAAATCCATCCAGAAACAATACACCGATTATTTTCACACC>1654
1492>AGAAACAGCTCGCTTAGGCGAGCATAGTAGATCTCACCTGCTCTCACGCATGCAAAGCCAAAAATCCATCCAGAAACAATACACCGATTATTTTCACACC>1591
1580>AGAAACAGCTCGCTTAGGCGAGCATAGTAGATCTCACCTGCTCTCACGCATGCAAAGCCAAAAATCCATCCAGAAACAATACACCGATTATTTTCACACC>1679

               *         *         *         *         *         *         *         *         *         *
1667>ATCAAAAGCACATCAAATTATTTATAAAAGCATGAAAAATCCTAGCTTCCCTCACCTGGACAAATGCTAGTGGAAAGCACAGGCACCTACGGCAACGAAC>1766
1701>ATCAAAAGCACATCAAATTATTTATAAAAGCATGAAAAATCCTAGCTTCCCTCACCTGGACAAATGCTAGTGGAAAGCACAGGCACCTACGGCAACGAAC>1800
1655>ATCAAAAGCACATCAAATTATTTATAAAAGCATGAAAAATCCTAGCTTCCCTCACCTGGACAAATGCTAGTGGAAAGCACAGGCACCTACGGCAACGAAC>1754
1592>ATCAAAAGCACATCAAATTATTTATAAAAGCATGAAAAATCCTAGCTTCCCTCACCTGGACAAATGCTAGTGGAAAGCACAGGCACCTACGGCAACGAAC>1691
1680>ATCAAAAGCACATCAAATTATTTATGAAAGCATGAAAAATCCTAGCTTCCCTCACCTGGACAAATGCTAGTGGAAAGCACAGGCACCTACGGCAACGAAC>1779

               *         *         *         *         *         *         *         *         *         *
1767>CCAACAACTTTAGGAGCATACTCAAGAGGTGGAGCAGAGAATGAGGTTGAGCTGACTTGAATCTGAATGAGCTTCGAGCCCTAGCAGAGCACTGTTTGAG>1866
1801>CCAACAACTTTAGGAGCATACTCAAGAGGTGGAGCAGAGAATGAGGTTGAGCTGACTTGAATCTGAATGAGCTTCGAGCCCTAGCAGAGCACTGTTTGAG>1900
1755>CCAACAACTTTAGGAGCATACTCAAGAGGTGGAGCAGAGAATGAGGTTGAGCTGACTTGAATCTGAATGAGCTTCGAGCCCTAGCAGAGCACTGTTTGAG>1854
1692>CCAACAACTTTAGGAGCATACTCAAGAGGTGGAGCAGAGAATGAGGTTGAGCTGACTTGAATCTGAATGAGCTTCGAGCCCTAGCAGAGCACTGTTTGAG>1791
1780>CCAACAACTTTAGGAGCATACTCAAGAGGTGGAGCAGAGAATGAGGTTGAGCTGACTTGAATCTGAATGAGCTTCGAGCCCTAGCAGAGCACTGTTTGAG>1879
```

```
              *         *         *         *         *         *         *         *         *         *
1867>GAGAAAAAAGGAGTAAGATGGTTGATTTTGCAAAGTGGGCAGAACATGAGAGGGTTAGGCTGGTTTAGGAGTTTTGGGATGTCCTATGGGCCAGCCTTAG>1966
1901>GAGAAAAAAGGAGTAAGATGGTTGATTTTGCAAAGTGGGCAGAACATGAGAGGGTTAGGCTGGTTTAGGAGTTTTGGGATGTCCTATGGGCCAGCCTTAG>2000
1855>GAGAAAAAAGGAGTAAGATGGTTGATTTTGCAAAGTGGGCAGAACATGAGAGGGTTAGGCTGGTTTAGGAGTTTTGGGATGTCCTATGGGCCAGCCTTAG>1954
1792>GAGAAAAAAGGAGTAAGATGGTTGATTTTGCAAAGTGGGCAGAACATGAGAGGGTTAGGCTGGTTTAGGAGTTTTGGGATGTCCTATGGGCCAGCCTTAG>1891
1880>GAGAAAAAAGGAGTAAGATGGTTGATTTTGCAAAGTGGGCAGAACATGAGAGGGTTAGGCTGGTTTAGGAGTTTTGGGATGTCCTATGGGCCAGCCTTAG>1979

              *         *         *         *         *         *         *         *         *         *
1967>GCCCAATTGACTAAGAGAGGCCCTTAAATTTTAGGACAAAATTAAAGTGAATCTTACATTAAGGATCTTCTTAAAATTCTTGTTCATCATCCTTATTAAT>2066
2001>GCCCAATTGACTAAGAGAGGCCCTTAAATTTTAGGACAAAATTAAAGTGAATCTTACATTAAGGATCTTCTTAAAATTCTTGTTCATCATCCTTATTAAT>2100
1955>GCCCAATTGACTAAGAGAGGCCCTTAAATTTTAGGACAAAATTAAAGTGAATCTTACATTAAGGATCTTCTTAAAATTCTTGTTCATCATCCTTATTAAT>2054
1892>GCCCAATTGACTAAGAGAGGCCCTTAAATTTTAGGACAAAATTAAAGTGAATCTTACATTAAGGATCTTCTTAAAATTCTTGTTCATCATCCTTATTAAT>1991
1980>GCCCAATTGACTAAGAGAGGCCCTTAAATTTTAGGACAAAATTAAAGTGAATCTTACATTAAGGATCTTCTTAAAATTCTTGTTCATCATCCTTATTAAT>2079

              *         *         *         *         *         *         *         *         *         *
2067>CATATCATGTTGCCATACATTGACGACATTCAATTTGTTTATTTATTTATTTTTTTACAAAAATAGTGTGTTCAACCTACTAGTCTACTTAGACTTTCCA>2166
2101>CATATCATGTTGCCATACATTGACGACATTCAATTTGTTTATTTATTTATTTTTTTACAAAAATAGTGTGTTCAACCTACTAGTCTACTTAGACTTTCCA>2200
2055>CATATCATGTTGCCATACATTGACGACATTCAATTTGTTTATTTATTTATTTTTTTACAAAAATAGTGTGTTCAACCTACTAGTCTACTTAGACTTTCCA>2154
1992>CATATCATGTTGCCATACATTGACGACATTCAATTTGTTTATTTATTTATTTTTTTACAAAAATAGTGTGTTCAACCTACTAGTCTACTTAGACTTTCCA>2091
2080>CATATCATGTTGCCATACATTGACGACATTCAATTTGTTTATTTATTTATTTTTTTACAAAAATAGTGTGTTCAACCTACTAGTCTACTTAGACTTTCCA>2179

              *         *         *         *         *         *         *         *         *         *
2167>TTGATAGATAAATCAACCCATAGTTAGTCATGATGGGGTAATAATAATATGGTATGGAAGATGTTTTTGTCCCTACATTTAATTATGCGTTGCATGATAT>2266
2201>TTGATAGATAAATCAACCCATAGTTAGTCATGATGGGGTAATAATAATATGGTATGGAAGATGTTTTTGTCCCTACATTTAATTATGCGTTGCATGATAT>2300
2155>TTGATAGATAAATCAACCCATAGTTAGTCATGATGGGGTAATAATAATATGGTATGGAAGATGTTTTTGTCCCTACATTTAATTATGCGTTGCATGATAT>2254
2092>TTGATAGATAAATCAACCCATAGTTAGTCATGATGGGGTAATAATAATATGGTATGGAAGATGTTTTTGTCCCTACATTTAATTATGCGTTGCATGATAT>2191
2180>TTGATAGATAAATCAACCCATAGTTAGTCATGATGGGGTAATAATAATATGGTATGGAAGATGTTTTTGTCCCTACATTTAATTATGCGTTGCATGATAT>2279

              *         *         *         *         *         *         *         *         *         *
2267>GATGAGCTCAATTAATCAGTATATTATTTTTCATGACTCATTGCATAAAAATATTATAATTGTACAAAATTAGTCGCCTGCGAACTCAACAATATGCACG>2366
2301>GATGAGCTCAATTAATCAGTATATTATTTTTCATGACTCATTGCATAAAAATATTATAATTGTACAAAATTAGTCGCCTGCGAACTCAACAATATGCACG>2400
2255>GATGAGCTCAATTAATCAGTATATTATTTTTCATGACTCATTGCATAAAAATATTATAATTGTACAAAATTAGTCGCCTGCGAACTCAACAATATGCACG>2354
2192>GATGAGCTCAATTAATCAGTATATTATTTTTCATGACTCATTGCATAAAAATATTATAATTGTACAAAATTAGTCGCCTGCGAACTCAACAATATGCACG>2291
2280>GATGAGCTCAATTAATCAGTATATTATTTTTCATGACTCATTGCATAAAAATATTATAATTGTACAAAATTAGTCGCCTGCGAACTCAACAATATGCACG>2379

              *         *         *         *         *         *         *         *         *         *
2367>TAAAAGTTACTTAATATATTTTTTATTTAAATTAGTTATTACAAAATATTTCAATTTTAAAAATATATTCAAATTTTAGAAATTAGTTATTATTTTAAAT>2466
2401>TAAAAGTTACTTAATATATTTTTTATTTAAATTAGTTATTACAAAATATTTCAATTTTAAAAATATATTCAAATTTTAGAAATTAGTTATTATTTTAAAT>2500
2355>TAAAAGTTACTTAATATATTTTTTATTTAAATTAGTTATTACAAAATATTTCAATTTTAAAAATATATTCAAATTTTAGAAATTAGTTATTATTTTAAAT>2454
2292>TAAAAGTTACTTAATATATTTTTTATTTAAATTAGTTATTACAAAATATTTCAATTTTAAAAATATATTCAAATTTTAGAAATTAGTTATTATTTTAAAT>2391
2380>TAAAAGTTACTTAATATATTTTTTATTTAAATTAGTTATTACAAAATATTTCAATTTTAAAAATATATTCAAATTTTAGAAATTAGTTATTATTTTAAAT>2479

              *         *         *         *         *         *         *         *         *         *
2467>TAATATTAAAATATTGTAAATATTTTATCATACATGACATATCAATAAACTAAAAAAAAACAAATTCCTTCATACATATATTTTATATATTTCTTATATA>2566
2501>TAATATTAAAATATTGTAAATATTTTATCATACATGACATATCAATAAACTAAAAAAAAACAAATTCCTTCATACATATATTTTATATATTTCTTATATA>2600
2455>TAATATTAAAATATTGTAAATATTTTATCATACATGACATATCAATAAACTAAAAAAAAACAAATTCCTTCATACATATATTTTATATATTTCTTATATA>2554
2392>TAATATTAAAATATTGTAAATATTTTATCATACATGACATATCAATAAACTAAAAAAAAACAAATTCCTTCATACATATATTTTATATATTTCTTATATA>2491
2480>TAATATTAAAATATTGTAAATATTTTATCATACATGACATATCAATAAACTAAAAAAAAACAAATTCCTTCATACATATATTTTATATATTTCTTATATA>2579

              *         *         *         *         *         *         *         *         *         *
2567>TTTCATGTATTTCATATATTAAAAGCCCTAAAATTTCTAATATATGGAGTTGTGATAAACACAAATTATGCAAACCCTTAAAAAATTCATAAATCCAAAC>2666
2601>TTTCATGTATTTCATATATTAAAAGCCCTAAAATTTCTAATATATGGAGTTGTGATAAACACAAATTATGCAAACCCTTAAAAAATTCATAAATCCAAAC>2700
2555>TTTCATGTATTTCATATATTAAAAGCCCTAAAATTTCTAATATATGGAGTTGTGATAAACACAAATTATGCAAACCCTTAAAAAATTCATAAATCCAAAC>2654
2492>TTTCATGTATTTCATATATTAAAAGCCCTAAAATTTCTAATATATGGAGTTGTGATAAACACAAATTATGCAAACCCTTAAAAAATTCATAAATCCAAAC>2591
2580>TTTCATGTATTTCATATATTAAAAGCCCTAAAATTTCTAATATATGGAGTTGTGATAAACACAAATTATGCAAACCCTTAAAAAATTCATAAATCCAAAC>2679

              *         *         *         *         *         *         *         *         *         *
2667>AATCACTCTACTATTTATTATTTTTTTACTTCTTCAAACTCACTGCTTAAAACAAAACCACAAAGATGACATTTTGTGACTATTTAACCCAATGCTCTATT>2766
2701>AATCACTCTACTATTTATTATTTTTTTACTTCTTCAAACTCACTGCTTAAAACAAAACCACAAAGATGACATTTTGTGACTATTTAACCCAATGCTCTATT>2800
2655>AATCACTCTACTATTTATTATTTTTTTACTTCTTCAAACTCACTGCTTAAAACAAAAACACAAAGATGACATTTTGTGACTATTTAACCCAATGCTCTATT>2754
2592>AATCACTCTACTATTTATTATTTTTTTACTTCTTCAAACTCACTGCTTAAAACAAAAACACAAAGATGACATTTTGTGACTATTTAACCCAATGCTCTATT>2691
2680>AATCACTCTACTATTTATTATTTTTTTACTTCTTCAAACTCACTGCTTAAAACAAAAACACAAAGATGACATTTTGTGACTATTTAACCCAATGCTCTATT>2779

              *         *         *         *         *         *         *         *         *         *
2767>TTGATTTGTCCTATTCTAAGAAAGACTAATCCTTTTTCTGATATCATTATTGTTGTTGATCACATTAATACAACTCAACAAACATGTGAATTTAAAATGA>2866
2801>TTGATTTGTCCTATTCTAAGAAAGACTAATCCTTTTTCTGATATCATTATTGTTGTTGATCACATTAATACAACTCAACAAACATGTGAATTTAAAATGA>2900
2755>TTGATTTGTCCTATTCTAAGAAAGACTAATCCTTTTTCTGATATCATTATTGTTGTTGATCACATTAATACAACTCAACAAACATGTGAATTTAAAATGA>2854
2692>TTGATTTGTCCTATTCTAAGAAAGACTAATCCTTTTTCTGATATCATTATTGTTGTTGATCACATTAATACAACTCAACAAACATGTGAATTTAAAATGA>2791
2780>TTGATTTGTCCTATTCTAAGAAAGACTAATCCTTTTTCTGATATCATTATTGTTGTTGATCACATTAATACAACTCAACAAACATGTGAATTTAAAATGA>2879
```

```
          *         *         *         *         *         *         *         *         *         *
2867>TGAATTTACTGCTTGCATTTTTGAGTAACATCTAGAAAAAAATGTATTTTCCTGGAAACAAGATTTTGAGGATGATGAAAGAAATAATTAAAAATATAAA>2966
2901>TGAATTTACTGCTTGCATTTTTGAGTAACATCTAGAAAAAAATGTATTTTCCTGGAAACAAGATTTTGAGGATGATGAAAGAAATAATTAAAAATATAAA>3000
2855>TGAATTTACTGCTTGCATTTTTGAGTAACATCTAGAAAAAAATGTATTTTCCTGGAAACAAGATTTTGAGGATGATGAAAGAAATAATTAAAAATATAAA>2954
2792>TGAATTTACTGCTTGCATTTTTGAGTAACATCTAGAAAAAAATGTATTTTCCTGGAAACAAGATTTTGAGGATGATGAAAGAAATAATTAAAAATATAAA>2891
2880>TGAATTTACTGCTTGCATTTTTGAGTAACATCTAGAAAAAAATGTATTTTCCTGGAAACAAGATTTTGAGGATGATGAAAGAAATAATTAAAAATATAAA>2979

          *         *         *         *         *         *         *         *         *         *
2967>GTAAGGTGTAAAGGAGTGGATGGGCTATGTTGTGAATTTGAGGGTTTGACCTGTCTCGTCAATTGTGGTAAGCCATATCACGCCCACAGCTAACGCCCCT>3066
3001>GTAAGGTGTAAAGGAGTGGATGGGCTATGTTGTGAATTTGAGGGTTTGACCTGTCTCGTCAATTGTGGTAAGCCATATCACGCCCACAGCTAACGCCCCT>3100
2955>GTAAGGTGTAAAGGAGTGGATGGGCTATGTTGTGAATTTGAGGGTTTGACCTGTCTCGTCAATTGTGGTAAGCCATATCACGCCCACAGCTAACGCCCCT>3054
2892>GTAAGGTGTAAAGGAGTGGATGGGCTATGTTGTGAATTTGAGGGTTTGACCTGTCTCGTCAATTGTGGTAAGCCATATCACGCCCACAGCTAACGCCCCT>2991
2980>GTAAGGTGTAAAGGAGTGGATGGGCTATGTTGTGAATTTGAGGGTTTGACCTGTCTCGTCAATTGTGGTAAGCCATATCACGCCCACAGCTAACGCCCCT>3079

          *         *         *         *         *         *         *         *         *         *
3067>CACGTGAGACTTTTGGAGTGACAGACAAGTTACAAGAATTGGTCTGGTATGGTTTCTCAACTCCCACTCTCACCTAACATTTCAGTTCACCTTCCTCCAG>3166
3101>CACGTGAGACTTTTGGAGTGACAGACAAGTTACAAGAATTGGTCTGGTATGGTTTCTCAACTCCCACTCTCACCTAACATTTCAGTTCACCTTCCTCCAG>3200
3055>CACGTGAGACTTTTGGAGTGACAGACAAGTTACAAGAATTGGTCTGGTATGGTTTCTCAACTCCCACTCTCACCTAACATTTCAGTTCACCTTCCTCCAG>3154
2992>CACGTGAGACTTTTGGAGTGACAGACAAGTTACAAGAATTGGTCTGGTATGGTTTCTCAACTCCCACTCTCACCTAACATTTCAGTTCACCTTCCTCCAG>3091
3080>CACGTGAGACTTTTGGAGTGACAGACAAGTTACAAGAATTGGTCTGGTATGGTTTCTCAACTCCCACTCTCACCTAACATTTCAGTTCACCTTCCTCCAG>3179

          *         *         *         *         *         *         *         *         *         *
3167>TATCATCAAAAACCAAACCTCAAACCCCAATCCTTAAAAACATCGAACAGTATCATTAATCCCACGTCCAATTCCCTTCCAATGGACAATTCAACCCAAG>3266
3201>TATCATCAAAAACCAAACCTCAAACCCCAATCCTTAAAAACATCGAACAGTATCATTAATCCCACGTCCAATTCCCTTCCAATGGACAATTCAACCCAAG>3300
3155>TATCATCAAAAACCAAACCTCAAACCCCAATCCTTAAAAACATCGAACAGTATCATTAATCCCACGTCCAATTCCCTTCCAATGGACAATTCAACCCAAG>3254
3092>TATCATCAAAAACCAAACCTCAAACCCCAATCCTTAAAAACATCGAACAGTATCATTAATCCCACGTCCAATTCCCTTCCAATGGACAATTCAACCCAAG>3191
3180>TATCATCAAAAACCAAACCTCAAACCCCAATCCTTAAAAACATCGAACAGTATCATTAATCCCACGTCCAATTCCCTTCCAATGGACAATTCAACCCAAG>3279

          *         *         *         *         *         *         *         *         *         *
3267>ATTCTCACCTGCGATCGGAAAACTCCGTCACCTATGAATCGCCATACCCAATTTACGCCATGGCTTTCTCACCCTCCCACCCCCACCGTCTCGCCCTCGG>3366
3301>ATTCTCACCTGCGATCGGAAAACTCCGTCACCTATGAATCGCCATACCCAATTTACGCCATGGCTTTCTCACCCTCCcacccccaccgtctcgccctcgg>3400
3255>ATTCTCACCTGCGATCGGAAAACTCCGTCACCTATGAATCGCCATACCCAATTTACGCCATGGCTTTCTCACCCTCCCACCCCcaccgtctcgccctcgg>3354
3192>ATTCTCACCTGCGATCGGAAAACTCCGTCACCTATGAATCGCCATACCCAATTTACGCCATGGCTTTCTCACCCTCCCACCCCCACCGTctcgccctcgg>3291
3280>ATTCTCACCTGCGATCGGAAAACTCCGTCACCTATGAATCGCCATACCCAATTTACGCCATGGCTTTCTCACCCTCCCACCCCCACCGTCTCGCCCTCGG>3379

          *         *         *         *         *         *         *         *         *         *
3367>AAGCTTCATTGAAGAATACACCAACCGCGTTCACATCCTCTCCTTCCACCCTGAAACCCTCTCCCTCTCCCCACACCCTTCCCTCTCCTTCGATCACCCT>3466
3401>aagcttcattgaagaatacaccaaccgcgttcacatcctctccttccaccctgaaaccctctccctctccccacacccttccctctccttcgatcaccct>3500
3355>aagcttcattgaagaatacaccaaccgcgttcacatcctctccttccaccctgaaaccctctccctctccccacaccCTTCCCTCTCCTTCGATCACCCT>3454
3292>aagcttcattgaagaatacaccaaccgcgttcacatcctctccttccaccCTGAAACCCTCTCCCTCTCCCCACACCCTTCCCTCTCCTTCGATCACCCT>3391
3380>AAGCTTCATTGAAGAATACACCAACCGCGTtcacatcctctccttccaccctgaaaccctctccctctccccacacccttccctctccttcgatcaccct>3479

          *         *         *         *         *         *         *         *         *         *
3467>TACCCTCCAACCAAGCTAATGTTCCATCCCCGCAAACCTTCCCCCTCTTCCTCCTCCGACCTCCTAGCCACCTCCGGCGACTACCTCCGCCTCTGGGACG>3566
3501>taccctccaaccaagctaatgttccatccccgcaaaccttccccctcttcctcctccgacctcctagccacctccggcgactacctccgcctctgggacg>3600
3455>TACCCTCCAACCAAGCTAATGTTCCATCCCCGCAAACCTTCCCCCTCTTCCTCCTCCGACCTCCTAGCCACCTCCGGCGACTACCTCCGCCTCTGGGACG>3554
3392>TACCCTCCAACCAAGCTAATGTTCCATCCCCGCAAACCTTCCCCCTCTTCCTCCTCCGACCTCCTAGCCACCTCCGGCGACTACCTCCGCCTCTGGGACG>3491
3480>taccctccaaccaagctaatgttccatCCCCGCAAACCTTCCCCCTCTTCCTCCTCCGACCTCCTAGCCACCTCCGGCGACTACCTCCGCCTCTGGGACG>3579

          *         *         *         *         *         *         *         *         *         *
3567>TCCGCGAAAACTCCGTCGAGCCCCTCTCCCTCTTCAACAACAGCAAGACCAGCGAGTTCTGCGCGCCCCTCACCTCCTTCGACTGGAACGACATCGATCA>3666
3601>tccgcgaaaactccgtcgagcccctctccctcttcaacaacagcaagaccagcgagttctgcgcgcccctcACCTCCTTCGACTGGAACGACATCGATCA>3700
3555>TCCGCGAAAACTCCGTCGAGCCCCTCTCCCTCTTCAACAACAGCAAGACCAGCGAGTTCTGCGCGCCCCTCACCTCCTTCGACTGGAACGACATCGATCA>3654
3492>TCCGCGAAAACTCCGTCGAGCCCCTCTCCCTCTTCAACAACAGCAAGACCAGCGAGTTCTGCGCGCCCCTCACCTCCTTCGACTGGAACGACATCGATCA>3591
3580>TCCGCGAAAACTCCGTCGAGCCCCTCTCCCTCTTCAACAACAGCAAGACCAGCGAGTTCTGCGCGCCCCTCACCTCCTTCGACTGGAACGACATCGATCA>3679

          *         *         *         *         *         *         *         *         *         *
3667>CAACCGCATCGGCACCTCCAGCATCGACACCACCTGCACCATCTGGGACATTGAGCGCACCGTCGTGGAGACGCAGCTCATCGCCCACGACAAGGAGGTC>3766
3701>CAACCGCATCGGCACCTCCAGCATCGACACCACCTGCACCATCTGGGACATTGAGCGCACCGTCGTGGAGACGCAGCTCATCGCCCACGACAAGGAGGTC>3800
3655>CAACCGCATCGGCACCTCCAGCATCGACACCACCTGCACCATCTGGGACATTGAGCGCACCGTCGTGGAGACGCAGCTCATCGCCCACGATAAGGAGGTC>3754
3592>CAACCGCATCGGCACCTCCAGCATCGACACCACCTGCACCATCTGGGACATTGAGCGCACCGTCGTGGAGACGCAGCTCATCGCCCACGATAAGGAGGTC>3691
3680>CAACCGCATCGGCACCTCCAGCATCGACACCACCTGCACCATCTGGGACATTGAGCGCACCGTCGTGGAGACGCAGCTCATCGCCCACGATAAGGAGGTC>3779

          *         *         *         *         *         *         *         *         *         *
3767>TACGACATCGCCTGGGGCGAGGCAAGAGTCTTCGCCTCCGTGTCCGCGGACGGCTCCGTACGAATCTTCGATTTGCGTGACAAAGAACACTCCACCATCA>3866
3801>TACGACATCGCCTGGGGCGAGGCAAGAGTCTTCGCCTCCGTGTCCGCGGACGGCTCCGTACGAATCTTCGATTTGCGTGACAAAGAACACTCCACCATCA>3900
3755>TACGACATCGCCTGGGGCGAGGCAAGAGTCTTCGCCTCCGTGTCCGCGGACGGCTCCGTACGAATCTTCGATTTGCGTGACAAAGAACACTCCACCATCA>3854
3692>TACGACATCGCCTGGGGCGAGGCAAGAGTCTTCGCCTCCGTGTCCGCGGACGGCTCCGTACGAATCTTCGATTTGCGTGACAAAGAACACTCCACCATCA>3791
3780>TACGACATCGCCTGGGGCGAGGCAAGAGTCTTCGCCTCCGTGTCCGCGGACGGCTCCGTACGAATCTTCGATTTGCGTGACAAAGAACACTCCACCATCA>3879
```

```
          *         *         *         *         *         *         *         *         *         *
3867>TCTACGAAAGCCCCCACCCGGATACCCCTTTGCTTCGTTTGGCTTGGAATAAGCAGGATCTCAGGTACATGGCTACCATCTTGATGGATAGTAATAAAGT>3966
3901>TCTACGAAAGCCCCCACCCGGATACCCCTTTGCTTCGTTTGGCTTGGAATAAGCAGGATCTCAGGTACATGGCTACCATCTTGATGGATAGTAATAAAGT>4000
3855>TCTACGAAAGCCCCCACCCGGATACCCCTTTGCTTCGTTTGGCTTGGAATAAGCAGGATCTCAGGTACATGGCTACCATCTTGATGGATAGTAATAAAGT>3954
3792>TCTACGAAAGCCCCCACCCGGATACCCCTTTGCTTCGTTTGGCTTGGAATAAGCAGGATCTCAGGTACATGGCTACCATCTTGATGGATAGTAATAAAGT>3891
3880>TCTACGAAAGCCCCCACCCGGATACCCCTTTGCTTCGTTTGGCTTGGAATAAGCAGGATCTCAGGTACATGGCTACCATCTTGATGGATAGTAATAAAGT>3979

          *         *         *         *         *         *         *         *         *         *
3967>TGTGATATTGGATATTAGGTCCCCCACTACTCCTGTGGCGGAATTAGAGAGGCATCGTGGGAGTGTCAATGCCATTGCTTGGGCTCCTCATAGCTCCACG>4066
4001>TGTGATATTGGATATTAGGTCCCCCACTACTCCTGTGGCGGAATTAGAGAGGCATCGTGGGAGTGTCAATGCCATTGCTTGGGCTCCTCATAGCTCCACG>4100
3955>TGTGATATTGGATATTAGGTCCCCCACTACTCCTGTGGCGGAATTAGAGAGGCATCGTGGGAGTGTCAATGCCATTGCTTGGGCTCCTCATAGCTCCACG>4054
3892>TGTGATATTGGATATTAGGTCCCCCACTACTCCTGTGGCGGAATTAGAGAGGCATCGTGGGAGTGTCAATGCCATTGCTTGGGCTCCTCATAGCTCCACG>3991
3980>TGTGATATTGGATATTAGGTCCCCCACTACTCCTGTGGCGGAATTAGAGAGGCATCGTGGGAGTGTCAATGCCATTGCTTGGGCTCCTCATAGCTCCACG>4079

          *         *         *         *         *         *         *         *         *         *
4067>CATATTTGTTCTGCTGGGGATGATACTCAGGCTCTTATCTGGGAATTGCCCACGCTCGCTGCTCCCACCGGGATTGATCCCATGTGCATGTATTCTGCTG>4166
4101>CATATTTGTTCTGCTGGGGATGATACTCAGGCTCTTATCTGGGAATTGCCCACGCTCGCTGCTCCCACCGGGATTGATCCCATGTGCATGTATTCTGCTG>4200
4055>CATATTTGTTCTGCTGGGGATGATACTCAGGCTCTTATCTGGGAATTGCCCACGCTCGCTGCTCCCACCGGGATTGATCCCATGTGCATGTATTCTGCTG>4154
3992>CATATTTGTTCTGCTGGGGATGATACTCAGGCTCTTATCTGGGAATTGCCCACGCTCGCTGCTCCCACCGGGATTGATCCCATGTGCATGTATTCTGCTG>4091
4080>CATATTTGTTCTGCTGGGGATGATACTCAGGCTCTTATCTGGGAATTGCCCACGCTCGCTGCTCCCACCGGGATTGATCCCATGTGCATGTATTCTGCTG>4179

          *         *         *         *         *         *         *         *         *         *
4167>GCTGTGAAATCAACCAGCTTCAATGGTCCGCGGCTCAGCCTGATTGGATTGCTATTGCTTTCGCCAACAAGGTGCAGCTTTTGAAGGTTTGAGGTGAGGT>4266
4201>GCTGTGAAATCAACCAGCTTCAATGGTCCGCGGCTCAGCCTGATTGGATTGCTATTGCTTTCGCCAACAAGGTGCAGCTTTTGAAGGTTTGAGGTGAGGT>4300
4155>GCTGTGAAATCAACCAGCTTCAATGGTCCGCGGCTCAGCCTGATTGGATTGCTATTGCTTTCGCCAACAAGGTGCAGCTTTTGAAGGTTTGAGGTGAGGT>4254
4092>GCTGTGAAATCAACCAGCTTCAATGGTCCGCGGCTCAGCCTGATTGGATTGCTATTGCTTTCGCCAACAAGGTGCAGCTTTTGAAGGTTTGAGGTGAGGT>4191
4180>GCTGTGAAATCAACCAGCTTCAATGGTCCGCGGCTCAGCCTGATTGGATTGCTATTGCTTTCGCCAACAAGGTGCAGCTTTTGAAGGTTTGAGGTGAGGT>4279

          *         *         *         *         *         *         *         *         *         *
4267>CACAAACAAACATAGTTAACTTGGTTGTGTGATAGATAGGACTCTTTTGCCGGATGCTGCTGCAACTTGTCGGTTCTCACTTGTTTTCTTTGGGGTTTTT>4366
4301>CACAAACAAACATAGTTAACTTGGTTGTGTGATAGATAGGACTCTTTTGCCGGATGCTGCTGCAACTTGTCGGTTCTCACTTGTTTTCTTTGGGGTTTTT>4400
4255>CACAAACAAACATAGTTAACTTGGTTGTGTGATAGATAGGACTCTTTTGCCGGATGCTGCTGCAACTTGTCGGTTCTCACTTGTTTTCTTTGGGGTTTTT>4354
4192>CACAAACAAACATAGTTAACTTGGTTGTGTGATAGATAGGACTCTTTTGCCGGATGCTGCTGCAACTTGTCGGTTCTCACTTGTTTTCTTTGGGGTTTTT>4291
4280>CACAAACAAACATAGTTAACTTGGTTGTGTGATAGATAGGACTCTTTTGCCGGATGCTGCTGCAACTTGTCGGTTCTCACTTGTTTTCTTTGGGGTTTTT>4379

          *         *         *         *         *         *         *         *         *         *
4367>AGCTTGTGGATTACAGTATGAGTGAGTAAGTGTATACCAGTTAGCGCAAACTAGTTTGCCTCTTCCTCTCTTGGGCCAAAATCGATGTTTCACTTGTTGT>4466
4401>AGCTTGTGGATTACAGTATGAGTGAGTAAGTGTATACCAGTTAGCGCAAACTAGTTTGCCTCTTCCTCTCTTGGGCCAAAATCGATGTTTCACTTGTTGT>4500
4355>AGCTTGTGGATTACAGTATGAGTGAGTAAGTGTATACCAGTTAGCGCAAACTAGTTTGCCTCTTCCTCTCTTGGGCCAAAATCGATGTTTCACTTGTTGT>4454
4292>AGCTTGTGGATTACAGTATGAGTGAGTAAGTGTATACCAGTTAGCGCAAACTAGTTTGCCTCTTCCTCTCTTGGGCCAAAATCGATGTTTCACTTGTTGT>4391
4380>AGCTTGTGGATTACAGTATGAGTGAGTAAGTGTATACCAGTTAGCGCAAACTAGTTTGCCTCTTCCTCTCTTGGGCCAAAATCGATGTTTCACTTGTTGT>4479

          *         *         *         *         *         *         *         *         *         *
4467>TTTGTTGAAAAGATGGAAATCATAGTTGTACAATGTTCAGTGTATGTATTCCCATTTGATCGTTACTAAATTCAAGCACCTTGCTCCTGCTACGAATGGA>4566
4501>TTTGTTGAAAAGATGGAAATCATAGTTGTACAATGTTCAGTGTATGTATTCCCATTTGATCGTTACTAAATTCAAGCACCTTGCTCCTGCTACGAATGGA>4600
4455>TTTGTTGAAAAGATGGAAATCATAGTTGTACAATGTTCAGTGTATGTATTCCCATTTGATCGTTACTAAATTCAAGCACCTTGCTCCTGCTACGAATGGA>4554
4392>TTTGTTGAAAAGATGGAAATCATAGTTGTACAATGTTCAGTGTATGTATTCCCATTTGATCGTTACTAAATTCAAGCACCTTGCTCCTGCTACGAATGGA>4491
4480>TTTGTTGAAAAGATGGAAATCATAGTTGTACAATGTTCAGTGTATGTATTCCCATTTGATCGTTACTAAATTCAAGCACCTTGCTCCTGCTACGAATGGA>4579

          *         *         *         *
4567>TCTGTGATTTTTAGCATCTGCTTTCCCGCCTCCATAACTTTGCTTTGC>4614
4601>TCTGTGATTTTTAGCATCTGCTTTCCCGCCTCCATAACTTTGCTTTGC>4648
4555>TCTGTGATTTTTAGCATCTGCTTTCCCGCCTCCATAACTTTGCTTTGC>4602
4492>TCTGTGATTTTTAGCATCTGCTTTCCCGCCTCCATAACTTTGCTTTGC>4539
4580>TCTGTGATTTTTAGCATCTGCTTTCCCGCCTCCATAACTTTGCTTTGC>4627
```
