# Supplementary material for: Seed Coat Pattern QTL and Development in Cowpea (Vigna unguiculata [L.] Walp.)
Source: Front Plant Sci. 2019 Oct 25;10:1346. doi: 10.3389/fpls.2019.01346 (PMC6824211; doi:10.3389/fpls.2019.01346)
Supplement: Supplementary file 4 [file DataSheet_3.pdf]

```
Tue Mar 12, 2019 16:54 PDT
Cowpea1.0_Vu10(old10)_38312100_38321146.fa from 1 to 9047
Alignment to
CowpeaA_LG10_32968200-32977262.fa--  Matches:8899; Mismatches:59; Gaps:194; Unattempted:0
CowpeaB_LG10_2879982-2889000.fa--  Matches:8894; Mismatches:63; Gaps:152; Unattempted:0
CowpeaC_LG10_30686060-30695060.fa--  Matches:8894; Mismatches:59; Gaps:142; Unattempted:0
CowpeaD_LG10_32826100-32835156.fa--  Matches:9030; Mismatches:0; Gaps:44; Unattempted:0

                  *         *         *         *         *         *         *         *         *         *
    1>~~~~~~~~~~~~~~~~~~~~~~~~~~~~~~~~~~~~~~~~~~~~~~~~~~~~~~~~CATATAAAACTAGTGGCTTGGCTTTTACTCACCGGTGATGAGGA>44
    1>ATGCCAATTTAAGAATTGTTTTGTCCTCAATTTCTTATTATTTTTTACAACTAGGACATATAAAACTAGTGGCTTGGCTTTTACTCACCGGTGATGAGGA>100
    1>TACAACTATGA---------------------------------------------CATATAAAACTAGTGGCTTGGCTTTTACTCACCGGTGATGAGGA>55
    1>~~~~~~~~~~~~~~~~~~~~~~~~~~~~~~~~~~~~~~~~~~~~~~~~~~~~~~~~~~~~~AAAACTAGTGGCTTGGCTTTTACTCACCGGTGATGAGGA>39
    1>~~~~~~~~~~~~~~~~~~~~~~~~~~~~~~~~~~~~~~~~~~~~~~~~~~~~~~~~~~~~~~~~~~~~~~~~~TTGGCTTTTACTCACCGGTGATGAGGA>27

                  *         *         *         *         *         *         *         *         *         *
   45>ATTAAAAACTCGTTATTTTAGGTCAAATGTAAAAAATAAAATTAAACCAAGCCATGAAAACTAAAGACTTACATAAACTATAAAACAAATACAAGATCAG>144
  101>ATTAAAAACTCGTTATTTTAGGTCAAATGTAAAAAATAAAATTAAACCAAGCCATGAAAACTAAAGACTTACATAAACTATAAAACAAATACAAGATCAG>200
   56>ATTAAAAACTCGTTATTTTAGGTCAAATGTAAAAAATAAAATTAAACCAAGCCATGAAAACTAAAGACTTACATAAACTATAAAACAAATACAAGATCAG>155
   40>ATTAAAAACTCGTTATTTTAGGTCAAATGTAAAAAATAAAATTAAACCAAGCCATGAAAACTAAAGACTTACATAAACTATAAAACAAATACAAGATCAG>139
   28>ATTAAAAACTCGTTATTTTAGGTCAAATGTAAAAAATAAAATTAAACCAAGCCATGAAAACTAAAGACTTACATAAACTATAAAACAAATACAAGATCAG>127

                  *         *         *         *         *         *         *         *         *         *
  145>TGTTTTATGTTCAACCATAACATAAAAAATATCAAAAGAAAAGTCATGATAAAACATAAAACAAAGAATGGTACTAATTAATTTTTGATGACCAAAAATAC>244
  201>TGTTTTATGTTCAACCATAACATAAAAAATATCAAAAGAAAAGTCATGATAAAACATAAAACAAAGAATGGTACTAATTAATTTTTGATGACCAAAAATAC>300
  156>TGTTTTATGTTCAACCATAACATAAAAAATATCAAAAGAAAAGTCATGATAAAACATAAAACAAAGAATGGTACTAATTAATTTTTGATGACCAAAAATAC>255
  140>TGTTTTATGTTCAACCATAACATAAAAAATATCAAAAGAAAAGTCATGATAAAACATAAAACAAAGAATGGTACTAATTAATTTTTGATGACCAAAAATAC>239
  128>TGTTTTATGTTCAACCATAACATAAAAAATATCAAAAGAAAAGTCATGATAAAACATAAAACAAAGAATGGTACTAATTAATTTTTGATGACCAAAAATAC>227

                  *         *         *         *         *         *         *         *         *         *
  245>ATGCAATTTGAGAACATTCTGAAAGAAAATTGAAGATTTAAGGAGAGCATTATTATTAATAAATGATTGTGAATATTTTTGGCAGAGGAATGACGTAAAG>344
  301>ATGCAATTTGAGAACATTCTGAAAGAAAATTGAAGATTTAAGGAGAACATTATTATTAATAAATGATTGTGAATATTTTTGGCAGAGGAATGACGTAAAG>400
  256>ATGCAATTTGAGAACATTCTGAAAGAAAATTGAAGATTTAAGGAGAACATTATTATTAATAAATGATTGTGAATATTTTTGGCAGAGGAATGACGTAAAG>355
  240>ATGCAATTTGAGAACATTCTGAAAGAAAATTGAAGATTTAAGGAGAACATTATTATTAATAAATGATTGTGAATATTTTTGGCAGAGGAATGACGTAAAG>339
  228>ATGCAATTTGAGAACATTCTGAAAGAAAATTGAAGATTTAAGGAGAGCATTATTATTAATAAATGATTGTGAATATTTTTGGCAGAGGAATGACGTAAAG>327

                  *         *         *         *         *         *         *         *         *         *
  345>ATTCCAAAACCCTAAAAACTGAAGAATAATCTAAACCTAACTCCACCTTCTTATTCTTCTTCCACGCTTTCACTTTCAAGAGTGACATCAAAATCTTTTG>444
  401>ATTCCAAAACCCTAAAAACTGAAGAATAATCTAAACCTAACTCCACCTTCTTATTCTTCTTCCACGCTTTCACTTTCAAGAGTGACATCAAAATCTTTTG>500
  356>ATTCCAAAACCCTAAAAACTGAAGAATAATCTAAACCTAACTCCACCTTCTTATTCTTCTTCCACGCTTTCACTTTCAAGAGTGACATCAAAATCTTTTG>455
  340>ATTCCAAAACCCTAAAAACTGAAGAATAATCTAAACCTAACTCCACCTTCTTATTCTTCTTCCACGCTTTCACTTTCAAGAGTGACATCAAAATCTTTTG>439
  328>ATTCCAAAACCCTAAAAACTGAAGAATAATCTAAACCTAACTCCACCTTCTTATTCTTCTTCCACGCTTTCACTTTCAAGAGTGACATCAAAATCTTTTG>427

                  *         *         *         *         *         *         *         *         *         *
  445>GTCCTTGGAATTGGGATATTCTTTGCTTCCACACCACATCTTCTACACTCACTTTCATTCTAATGAATTCAGTTTTCCTTCACTAATTCGGTTTCATTCC>544
  501>GTCCTTGGAATTGGGATATTCTTTGCTTCCACACCACATCTTCTACACTCACTTTCATCCTAATGAATTCAGTTTTCCTTCACTAATTCGGTTTCATTCC>600
  456>GTTCTTGGAATTGGGATATTCTTTGCTTCCACACCACATCTTCTACACTCACTTTCATCCTAATGAATTCAGTTTTCCTTCACTAATTCGGTTTCATTCC>555
  440>GTCCTTGGAATTGGGATATTCTTTGCTTCCACACCACATCTTCTACACTCACTTTCATCCTAATGAATTCAGTTTTCCTTCACTAATTCGGTTTCATTCC>539
  428>GTCCTTGGAATTGGGATATTCTTTGCTTCCACACCACATCTTCTACACTCACTTTCATTCTAATGAATTCAGTTTTCCTTCACTAATTCGGTTTCATTCC>527

                  *         *         *         *         *         *         *         *         *         *
  545>AAATAACTTTATAGTAGTTAAAAAGTTGTGACTTCTTTTCATCTATTCTATTATTCATCTATTCTTCTCATCTGGTTTTCTTGTGTACACTTGAGAAATA>644
  601>AAATAACTTTATAGTAGTTAAAAAGTTGTGACTTCTTTTCAT----------------CTATTCTTCTCATCTGGTTTTCTTGTGTACACTTGAGAAACA>684
  556>AAATAACTTTATAGTAGTTAAAAAGTTGTGACTTCTTTTCAT----------------CTATTCTTCTCATCTGGTTTTCTTGTGTACACTTGAGAAACA>639
  540>AAATAACTTTATAGTAGTTAAAAAGTTGTGACTTCTTTTCAT----------------CTATTCTTCTCATCTGGTTTTCTTGTGTACACTTGAGAAACA>623
  528>AAATAACTTTATAGTAGTTAAAAAGTTGTGACTTCTTTTCATCTATTCTATTATTCATCTATTCTTCTCATCTGGTTTTCTTGTGTACACTTGAGAAATA>627

                  *         *         *         *         *         *         *         *         *         *
  645>TGAAATTTTGTATTTAATATAAAATAATATTACGATTAATATATTATAAGAAATGTCTTTTGTTGTTTATATTTAAATTCTCACAGTCTTTAACATCTGC>744
  685>TGAAATTTTGTATTTAATATAAAATAATATTACGATTAATATATTATAAGAAATGTCTTTTGTTGTTTATATTTAAATTCTCACAGTCTTTAACATCTGC>784
  640>TGAAATTTTGTATTTAATATAAAATAATATTACGATTAATATATTATAAGAAATGTCTTTTGTTGTTTATATTTAAATTCTCACAGTCTTTAACATCTGC>739
  624>TGAAATTTTGTATTTAATATAAAATAATATTACGATTAATATATTATAAGAAATGTCTTTTGTTGTTTATATTTAAATTCTCACAGTCTTTAACATCTGC>723
  628>TGAAATTTTGTATTTAATATAAAATAATATTACGATTAATATATTATAAGAAATGTCTTTTGTTGTTTATATTTAAATTCTCACAGTCTTTAACATCTGC>727

                  *         *         *          *         *         *         *         *         *
  745>AAACATGAAAATAAGACAACAAGACAAAATAA-TAGTCATATTATTCAATTAATAAAAAAGATAATATTAAAAATCAATTACGTGAGACTTAAAATTTAA>843
  785>AAACATGAAAATAAGACAACAAGACAAAATAA-TAGTCATATTATTCAATTAATAAAAAAGATAATATTAAAAATCAATTACGTGAGACTTAAAATTTAA>883
  740>AAACATGAAAATAAGACAACAAGACAAAATAAAAAGTCATATTATTCAATTAATAAAAAAGATAATATTAAAAATCAATTACGTGAGACTTAAAATTTAA>839
  724>AAACATGAAAATAAGACAACAAGACAAAATAA-TAGTCATATTATTCAATTAATAAAAAAGATAATATTAAAAATCAATTACGTGAGACTTAAAATTTAA>822
  728>AAACATGAAAATAAGACAACAAGACAAAATAA-TAGTCATATTATTCAATTAATAAAAAAGATAATATTAAAAATCAATTACGTGAGACTTAAAATTTAA>826
```

```
       *         *         *         *         *         *         *         *         *         *
 844>TTAAATCTAAATAAATTTAACAAGTGAAGTTTTCTTTTATGATTAGTCAAACTCCAGTTTTTAACTAGATATTTTTTGAACGATTGGGCTTGATGGGGTA>943
 884>TTAAATCTAAATAAATTTAGCAAGTGAAGTTTTCTTTTATGATTAGTCAAACTCCAGTTTTTAACTAGATATTTTTTGAACGATTGGGCTTGATGGGGTA>983
 840>TTAAATCTAAATAAATTTAACAAGTGAAGTTTTCTTTTATGATTAGTCAAACTCCAGTTTTTAACTAGATATTTTTTGAACGATTGGGCTTGATGGGGTA>939
 823>TTAAATCTAAATAAATTTAGCAAGTGAAGTTTTCTTTTATGATTAGTCAAACTCCAGTTTTTAACTAGATATTTTTTGAACGATTGGGCTTGATGGGGTA>922
 827>TTAAATCTAAATAAATTTAACAAGTGAAGTTTTCTTTTATGATTAGTCAAACTCCAGTTTTTAACTAGATATTTTTTGAACGATTGGGCTTGATGGGGTA>926

       *         *         *         *         *         *         *         *         *         *
 944>AATCATGATTAAATTGTAGAAATCCACTTCATGGACCCAACAATAAATTTTTTTTCAGTCATCTCCTTTCGGGCTGCTGCTGCTTCTTGCACCCTCTAAA>1043
 984>AATCATGATTAAATTGTAGAAATCCACTTCATGGACCCAACAATAAATATTTTTTCAGTCATCTCCTTTCGGGCTGCTGCTGCTTCTTGCACCCTCTAAA>1083
 940>AATCATGATTAAATTGTAGAAATCCACTTCATGGACCCAACAATAAATATTTTTTCAGTCATCTCCTTTCGGGCTGCTGCTGCTTCTTGCACCCTCTAAA>1039
 923>AATCATGATTAAATTGTAGAAATCCACTTCATGGACCCAACAATAAATATTTTTTCAGTCATCTCCTTTCGGGCTGCTGCTGCTTCTTGCACCCTCTAAA>1022
 927>AATCATGATTAAATTGTAGAAATCCACTTCATGGACCCAACAATAAATTTTTTTTCAGTCATCTCCTTTCGGGCTGCTGCTGCTTCTTGCACCCTCTAAA>1026

       *         *         *         *         *         *         *         *         *         *
1044>TTTTCTTCTATATCCTTTTGTATGAAACTACCATTTTGTCCTTTATTATTTTATAATAACATCAATCTTTGATTTTTTTTTTTTTTAAATTGGACATCAAT>1143
1084>TTTTCTTCTATATCCTTTTGTATGAAACTACCATTTTGTCCTTTATTATTTTATAATAACATCAATCTTTGATTTTTTTTTTTT--AAATTGGACATCAAT>1181
1040>TTTTCTTCTATATCCTTTTGTATGAAACTACCATTTTGTCCTTTATTATTTTATAATAACATCAATCTTTGATTTTTTTTTTT---AAATTGGCCATCAAT>1136
1023>TTTTCTTCTATATCCTTTTGTATGAAACTACCATTTTGTCCTTTATTATTTTATAATAACATCAATCTTTGATTTTTTTTTTTT--AAATTGGACATCAAT>1120
1027>TTTTCTTCTATATCCTTTTGTATGAAACTACCATTTTGTCCTTTATTATTTTATAATAACATCAATCTTTGATTTTTTTTTTTTTTAAATTGGACATCAAT>1126

       *         *         *         *         *         *         *          *         *         *
1144>GCAATTAAATTTTAAGATATTCCTTTACACTCAAAACCTTACCATAATATGTTTTCAATTTTTT-ATATATTATTAATTCTGTTATTTCTACTTAAATTC>1242
1182>GCAATTAAATTTTAAGATATTCCTTTACACTCAAAACCTTACCATAATATGTTTTCAATTTTTTTATATATTATTAATTCTGTTATTTCTACTTAAATTC>1281
1137>GCAATTAAATTTTAAGATATTCCTTTACACTCAAAACCTTACCATAATATGTTTTCAATTTTTTTATATATTATTAATTCTGTTATTTCTACTTAAATTC>1236
1121>GCAATTAAATTTTAAGATATTCCTTTACACTCAAAACCTTACCATAATATGTTTTCAATTTTTTTATATATTATTAATTCTGTTATTTCTACTTAAATTC>1220
1127>GCAATTAAATTTTAAGATATTCCTTTACACTCAAAACCTTACCATAATATGTTTTCAATTTTTTTATATATTATTAATTCTGTTATTTCTACTTAAATTC>1226

        *         *         *         *         *         *         *         *         *         *
1243>GCATTTAAAATATTAAAAAAAGTTATTTCTTTTCACATATTTATATATTTTAATATCTTATATATTTATCTTTTTAGTTTAATAACATTCACGTAATAAG>1342
1282>GCATTTAAAATATTAAAAAAAGTTATTTCTTTTCACATATTTATATATTTTAATATCTTATATATTTATCTTTTTAGTTTAATAACATTCACGTAATAAG>1381
1237>GCATTTAAAATATTAAAAAAAGTTATTTCTTTTCACATATTTATATATTTTAATATCTTATATATTTATCTTTTTAGTTTAATAACATTCACGTAATAAG>1336
1221>GCATTTAAAATATTAAAAAAAGTTATTTCTTTTCACATATTTATATATTTTAATATCTTATATATTTATCTTTTTAGTTTAATAACATTCACGTAATAAG>1320
1227>GCATTTAAAATATTAAAAAAAGTTATTTCTTTTCACATATTTATATATTTTAATATCTTATATATTTATCTTTTTAGTTTAATAACATTCACGTAATAAG>1326

        *         *         *         *         *         *         *         *         *         *
1343>AATGAAAGTAATACATTCAGCTTTTAAGATATCAAAATACGAAATCAAAAGCTATTACCACCATGCATAATTAACTCTCAGTAGTTTCTGATATTCATAC>1442
1382>AATGAAAGTAATACATTCAGCTTTTAAGATATCAAAATACGAAATCAAAAGCTATTACCACCATGCATAATTAACTCTCAGTAGTTTCTGATATTCATAC>1481
1337>AATGAAAGTAATACATTCAGCTTTTAAGATATCAAAATACGAAATCAAAAGCTATTACCACCATGCATAATTAACTCTCAGTAGTTTCTGATATTCATAC>1436
1321>AATGAAAGTAATACATTCAGCTTTTAAGATATCAAAATACGAAATCAAAAGCTATTACCACCATGCATAATTAACTCTCAGTAGTTTCTGATATTCATAC>1420
1327>AATGAAAGTAATACATTCAGCTTTTAAGATATCAAAATACGAAATCAAAAGCTATTACCACCATGCATAATTAACTCTCAGTAGTTTCTGATATTCATAC>1426

        *         *         *         *         *         *         *         *         *         *
1443>GTTAAAAATATAACACATATACATAAAATGCAACACTCTGAAACAAAAAAAACGAGAGATTATAACGATTTCATGCTTCTATCTCCATAAGAATTCTTGA>1542
1482>GTTAAAAATCTAACACATATACATAAAATGCAACACTCAGAAACAAAAAAAACGAGAGATTATAACGATTTCATGCTTCTATCTCCATAAGAATTCTTGA>1581
1437>GTTAAAAATCTAACACATATACATAAAATGCAACACTCAGAAACAAAAAAAACGAGAGATTATAACGATTTCATGCTTCTATCTCCATAAGAATTCTTGA>1536
1421>GTTAAAAATCTAACACATATACATAAAATGCAACACTCAGAAACAAAAAAAACGAGAGATTATAACGATTTCATGCTTCTATCTCCATAAGAATTCTTGA>1520
1427>GTTAAAAATATAACACATATACATAAAATGCAACACTCTGAAACAAAAAAAACGAGAGATTATAACGATTTCATGCTTCTATCTCCATAAGAATTCTTGA>1526

        *         *         *         *         *         *         *         *         *         *
1543>ACTGAGGCTTGCCAGTGAAGAACAAGCATGAGCATAACCAAAATCATCCCCAAACCCCAGGAAGAGCTTCCAACTCTGAACACCCACGAAGGCTCAGGAG>1642
1582>ACTGAGGCTTGCCAGTGAAGAACAAGCATGAGCATAACCAAAATCATCCCCAAACCCCAGGAAGAGCTTCCAACTCTGAATACCCATGAAGGCTCAGGAG>1681
1537>AGTGAGGCTTGCCAGTGAAGAACAAGCATGAGCATAACCAAAATCATCCCCAAACCCCAAGAAGAGCTTCCAACTCTGAATACCCATGAAGGCTCAGGAG>1636
1521>ACTGAGGCTTGCCAGTGAAGAACAAGCATGAGCATAACCAAAATCATCCCCAAACCCCAGGAAGAGCTTCCAACTCTGAATACCCATGAAGGCTCAGGAG>1620
1527>ACTGAGGCTTGCCAGTGAAGAACAAGCATGAGCATAACCAAAATCATCCCCAAACCCCAGGAAGAGCTTCCAACTCTGAACACCCACGAAGGCTCAGGAG>1626

        *         *         *         *         *         *         *         *         *         *
1643>AAGGTAGAAAGGAAGCTCTTGGTGTTGATGATGATGAAGACACAGAAGAAGAGGTTGTTCTTATGGCAATTATGAGACCAGCAAGAAACATAGGCAGAAC>1742
1682>AGGGTAGAAACGAAGCTCTCGGTGTTGATGATGATGAAGACACAGAAGAAGAGGTTGTTCTGATGGCAATTATGAGACCAGCAAGAAACATAGGCAGAAC>1781
1637>AGGGTAGAAACGAAGCTCTCGGTGTTGATGATGATGAAGACACAGAAGAAGAGGTTGTTCTGATGGCAATTATGAGACCAGCAAGAAACATAGGCAGAAC>1736
1621>AGGGTAGAAACGAAGCTCTCGGTGTTGATGATGATGAAGACACAGAAGAAGAGGTTGTTCTGATGGCAATTATGAGACCAGCAAGAAACATAGGCAGAAC>1720
1627>AAGGTAGAAAGGAAGCTCTTGGTGTTGATGATGATGAAGACACAGAAGAAGAGGTTGTTCTTATGGCAATTATGAGACCAGCAAGAAACATAGGCAGAAC>1726

        *         *         *         *         *         *         *         *         *         *
1743>AAAACCACCAGAGCTCCATTTTGATTCATAGTACCTTCTGTCACTTCTTGAATTCCCATACAACGGAGACAACACCACTGCAGAGACAAAAGCCAACACC>1842
1782>AAAACCACCAGAGCTCCATTTTGATTCATAGTACCTTCTGTCACTTCTTGAATTCCCATACAACGGAGACAACACCACTGCAGAGACAAAAGCCAACACC>1881
1737>AAAACCACCAGAGCTCCATTTTGATTCATAGTACCTTCTGTCACTTCTTGAATTCCCATACAACGGAGACAACACCACTGCAGAGACAAAAGCCAACACC>1836
1721>AAAACCACCAGAGCTCCATTTTGATTCATAGTACCTTCTGTCACTTCTTGAATTCCCATACAACGGAGACAACACCACTGCAGAGACAAAAGCCAACACC>1820
1727>AAAACCACCAGAGCTCCATTTTGATTCATAGTACCTTCTGTCACTTCTTGAATTCCCATACAACGGAGACAACACCACTGCAGAGACAAAAGCCAACACC>1826
```

```
           *         *         *         *         *         *         *         *         *         *
1843>ATGGCCAATGATCT-----ATG-TGATGAAGAAGAAGAAGCATAGGTAGTGGTGGTGGCCATAACTTGAAGCTGAATTTGTTTTGTGTATGATGAAAATC>1936
1882>ATGGCCAATGATCTCTGTGATGATGATGAAGAAGAAGAAGCATAGGTAGTGGTGATGGCCATAACTTGAAGCTGAATTTGTTTTGTGTATGATGAAAATC>1981
1837>ATGGCCAATGATCTCTGTGATGATGATGAAGAAGAAGAAGCATAGGTAGTGGTGATGGCCATAACTTGAAGCTGAATTTGTTTTGTGTATGATGAAAATC>1936
1821>ATGGCCAATGATCTCTGTGATGATGATGAAGAAGAAGAAGCATAGGTAGTGGTGATGGCCATAACTTGAAGCTGAATTTGTTTTGTGTATGATGAAAATC>1920
1827>ATGGCCAATGATCT-----ATG-TGATGAAGAAGAAGAAGCATAGGTAGTGGTGGTGGCCATAACTTGAAGCTGAATTTGTTTTGTGTATGATGAAAATC>1920

           *         *         *         *         *         *         *         *         *         *
1937>TGAGGTTATGAAGAAAGAATATATAGTGGGAGGAAAAGGTGATTAGGCTAAGGAATTATATAATAATGTATGTTGAATGATGCAGAAACAACCGGCCAAAAA>2036
1982>TGAGGTTATGAAGAAAGAATATATAGTGGGAGGAAAAGGTGATTAGGCTAAGGAATTATATAATAATGTATGTTGAATGATGCAGAAACAACCGGCCAAAAA>2081
1937>TGAGGTTATGAAGAAAGAATATATAGTGGGAGGAAAAGGTGATTAGGCTAAGGAATTATATAATAATGTATGTTGAATGATGCAGAAACAACCGGCCAAAAA>2036
1921>TGAGGTTATGAAGAAAGAATATATAGTGGGAGGAAAAGGTGATTAGGCTAAGGAATTATATAATAATGTATGTTGAATGATGCAGAAACAACCGGCCAAAAA>2020
1921>TGAGGTTATGAAGAAAGAATATATAGTGGGAGGAAAAGGTGATTAGGCTAAGGAATTATATAATAATGTATGTTGAATGATGCAGAAACAACCGGCCAAAAA>2020

           *         *         *         *         *         *         *         *         *         *
2037>GTGAAGAGGATTAAAGAATGGACACAAAAGATAAAAGAGAATATAAAGGTGAAAAGGATGGTGGAAAAAGGAGATTCAGGGTATGGAAGTAAGGTATTTT>2136
2082>GTGAAGAGGATTAAAGAATGGACAGAAAAGATAAAAGAGAATATAAAGGTGAAAAGGATGGTGGAAAAAGGAGATTCAGGGTATGGAAGTAAGGTATTTT>2181
2037>GTGAAGAGGATTAAAGAATGGACAGAAAAGATAAAAGAGAATATAAAGGTGAAAAGGATGGTGGAAAAAGGAGATTCAGGGTATGGAAGTAAGGTATTTT>2136
2021>GTGAAGAGGATTAAAGAATGGACAGAAAAGATAAAAGAGAATATAAAGGTGAAAAGGATGGTGGAAAAAGGAGATTCAGGGTATGGAAGTAAGGTATTTT>2120
2021>GTGAAGAGGATTAAAGAATGGACACAAAAGATAAAAGAGAATATAAAGGTGAAAAGGATGGTGGAAAAAGGAGATTCAGGGTATGGAAGTAAGGTATTTT>2120

           *         *         *         *         *         *         *         *         *         *
2137>TCTTAGCTTTTTGTTTGTGTTCACATTGTTATGTGTTGTTGATGAAGAAGCTATAGCTGTAACCCATGTTTTATAGATTGGAGATGAGAGTAGCTGTAAT>2236
2182>TCTTAGCTTTTTGTTTGTGTTCACATTGTTATGTGTTGTTGATGAAGAAGCTATAGCTGTAAGCCATGTTTTATAGATTGGAGATGAGAGTAGCTGTAAT>2281
2137>TCTTAGCTTTTTGTTTGTGTTCACATTGTTATGTGTTGTTGATGAAGAAGCTATAGCTGTAAGCCATGTTTTATAGATTGGAGATGAGAGTAGCTGTAAT>2236
2121>TCTTAGCTTTTTGTTTGTGTTCACATTGTTATGTGTTGTTGATGAAGAAGCTATAGCTGTAAGCCATGTTTTATAGATTGGAGATGAGAGTAGCTGTAAT>2220
2121>TCTTAGCTTTTTGTTTGTGTTCACATTGTTATGTGTTGTTGATGAAGAAGCTATAGCTGTAACCCATGTTTTATAGATTGGAGATGAGAGTAGCTGTAAT>2220

           *         *         *         *         *         *         *         *         *         *
2237>CAAAGTGACAAGCACTGCATCATTAGCAACAACAATTTGGATTACTTTTTGACAGTGTTGTCAGAAAGAGAAACTTGGGTTTTGTGTTTTTCATTTGATC>2336
2282>CAAAGTGACAAGCACTGCATCATTAGCAACAACAATTTGGATTACTTTTTGACAGTGTTGTCAGAAAGAGAAACTTGGGTTTTGTGTTTTTCATTTGATC>2381
2237>CAAAGTGACAAGCACTGCATCATTAGCAACAACAATTTGGATTACTTTTTGACAGTGTTGTCAGAAAGAGAAACTTGGGTTTTGTGTTTTTCATTTGATC>2336
2221>CAAAGTGACAAGCACTGCATCATTAGCAACAACAATTTGGATTACTTTTTGACAGTGTTGTCAGAAAGAGAAACTTGGGTTTTGTGTTTTTCATTTGATC>2320
2221>CAAAGTGACAAGCACTGCATCATTAGCAACAACAATTTGGATTACTTTTTGACAGTGTTGTCAGAAAGAGAAACTTGGGTTTTGTGTTTTTCATTTGATC>2320

                 *         *         *         *         *         *         *         *         *
2337>AATG---TTTTTGAAACCAAAACTAAACCCTTTATTCTTATTAATAAGAAAGAAAGAAAGGAATGCAGAAAGTATGGTAGTCAGGCCTCCAAAAA----->2428
2382>AATGAGGTTTTTCAAACCAAAACTAAAGCCT---------TTAATAAGAAAGAAAGAAAGGAATGCAGAAAGTATGGTAGTCAGGCCTCCAAAAATTCCA>2472
2337>AATGAGGTTTTTCAAACCAAAACTAAAGCCT---------TTAATAAGAAAGAAAGAAAGGAATGCAGAAAGTATGGTAGTCAGGCCTCCAAAAATTCCA>2427
2321>AATGAGGTTTTTCAAACCAAAACTAAAGCCT---------TTAATAAGAAAGAAAGAAAGGAATGCAGAAAGTATGGTAGTCAGGCCTCCAAAAATTCCA>2411
2321>AATG---TTTTTGAAACCAAAACTAAACCCTTTATTCTTATTAATAAGAAAGAAAGAAAGGAATGCAGAAAGTATGGTAGTCAGGCCTCCAAAAA----->2412

            *         *         *         *         *         *         *         *            *
2429>---AGCAAACATTTATACAATCAATTACCAAACTTTACCTGTTTATTTTACAGGTCTGAATTCGGTTTGGAAACGAACTTTCAAGGTG---AT-----TT>2517
2473>AAAAGCAAACATTTATATAATCAATTACCAAACTTTACCTGTTTATTTTACAGGTCTGAATTCGGTTTGGAAACGAACTTTCAAGGTG---TTTTTT-TT>2568
2428>AAAAGCAAACATTTATATAATCAATTACCAAACTTTACCTGTTTATTTTACAGGTCTGAATTCGGTTTGGAAACGAACTTTCAAGGTG---ttttttttt>2524
2412>AAAAGCAAACATTTATATAATCAATTACCAAACTTTACCTGTTTATTTTACAGGTCTGAATTCGGTTTGGAAACGAACTTTCAAGGTGTTTTT-----TT>2506
2413>---AGCAAACATTTATACAATCAATTACCAAACTTTACCTGTTTATTTTACAGGTCTGAATTCGGTTTGGAAACGAACTTTCAAGGTG---AT-----TT>2501

         *         *         *         *         *         *         *         *         *         *
2518>TGTTTTTGTTTTTCTATTGTATTCTACAAATTTCAAAAAAATAAACATGAAGCTTTATTTTATGGATATTATGATAAATATAACATATATATATATATATA>2617
2569>TTTTTTTGTTTTTCTAttgtattttaCAAATTTCAAAAAAATAAACATGAAGCTTTATTTTATGGATATTATGA--------------------------->2641
2525>tTTTTTTGTTTTTCTATTGTATTTTACAAATTTCAAAAAAATAAACATGAAGCTTTATTTTATGGATATTATGA--------------------------->2597
2507>TTTTTTTGTTTTTCTATTGTATTTTACAAATTTCAAAAAAATAAACATGAAGCTTTATTTTATGGATATTATGA--------------------------->2579
2502>TGTTTTTGTTTTTCTATTGTATTCTACAAATTTCAAAAAAATAAACATGAAGCTTTATTTTATGgatattatgataaatataacatatatatatatatata>2601

         *         *                                    *         *         *          *         *
2618>TATATATATATATATATATATAT--------------------------ATATATAAATATGTTATGTATATATAGA-AGATTATATTTATAAATAATAT>2690
2642>----------GATATATATATAT--------------------------ATATATAAATATGTTATGTATATATAAATAGATTATATTTATAAATAACAT>2705
2598>----------GATATATATATAT--------------------------ATATATAAATATGTTATGTATATATAAATAGATTATATTTATAAATAACAT>2661
2580>----------GATATATATATAT--------------------------ATATATAAATATGTTATGTATATATAAATAGATTATATTTATAAATAACAT>2643
2602>tatatatatatatatatatatatatatatatatatatatatatatatataatatataaatatgTTATGTATATATAGA-AGATTATATTTATAAATAATAT>2700

           *         *         *         *         *         *         *         *         *         *
2691>ACT----AATAGTTAGTTTATCAAGTAAAATAAATTAATAAATATTTTTTCAAAAATAAACAATTTAATTTAATGTATATTTATCATTATTTAAAAAGAA>2786
2706>ACTAAAAAATAGTTAGTTTATCAAGTAAAATAAATTAATAAATATTTTTTCAAAAATAAACAATTTAATTTAATGTATATTTATCATTATTTAAAAAGAA>2805
2662>ACTAAAAAATAGTTAGTTTATCAAGTAAAATAAATTAATAAATATTTTTTCAAAAATAAACAATTTAATTTAATGTATATTTATCATTATTTAAAAAGAA>2761
2644>ACTAAAAAATAGTTAGTTTATCAAGTAAAATAAATTAATAAATATTTTTTCAAAAATAAACAATTTAATTTAATGTATATTTATCATTATTTAAAAAGAA>2743
2701>ACT----AATAGTTAGTTTATCAAGTAAAATAAATTAATAAATATTTTTTCAAAAATAAACAATTTAATTTAATGTATATTTATCATTATTTAAAAAGAA>2796
```

```
          *         *         *         *         *         *         *         *         *         *
2787>ATAATTTATACTTTAAAAATATATATATAATTAATATTTCATAAGTAAATCAGTAAATTTAATACAACAAAAATGAATGTACAATTTAATATAATTATAA>2886
2806>ATAATTTATACTTTAAAAATATATATA--ATT-ATATTTCATAAGTAAATCAGTAAATTTAATACAACAAAAATGAATGTACAATTTAATATAATTATAA>2902
2762>ATAATTTATACTTTAAAAATATATATA--ATT-ATATTTCATAAGTAAATCAGTAAATTTAATACAACAAAAATGAATGTACAATTTAATATAATTATAA>2858
2744>ATAATTTATACTTTAAA--TATATATATAATT-ATATTTCATAAGTAAATCAGTAAATTTAATACAACAAAAATGAATGTACAATTTAATATAATTATAA>2840
2797>ATAATTTATACTTTAAAAATATATATATAATTAATATTTCATAAGTAAATCAGTAAATTTAATACAACAAAAATGAATGTACAATTTAATATAATTATAA>2896

          *         *         *         *         *         *         *         *         *         *
2887>ATAGAAAATAAATCCCACATATCTAATATATTTTTCTTTGTAATCAAGTCAAAACTAAAATTAAGTGAATACTAAACTTTTTAGTTGTATTTTATTCTAT>2986
2903>ATATAAAATAAATCCCACATATCTAATATATTTTTCTTTGTAATCAAGTCAAAACTAAAATTAAGTGAATACTAAACTTTTTAGTTGTATTTTATTCTAT>3002
2859>ATATAAAATAAATCCCACATATCTAATATATTTTTCTTTGTAATCAAGTCAAAACTAAAATTAAGTGAATACTAAACTTTTTAGTTGTATTTTATTCTAT>2958
2841>ATATAAAATAAATCCCACATATCTAATATATTTTTCTTTGTAATCAAGTCAAAACTAAAATTAAGTGAATACTAAACTTTTTAGTTGTATTTTATTCTAT>2940
2897>ATAGAAAATAAATCCCACATATCTAATATATTTTTCTTTGTAATCAAGTCAAAACTAAAATTAAGTGAATACTAAACTTTTTAGTTGTATTTTATTCTAT>2996

          *         *         *         *         *         *         *         *         *         *
2987>GTATATAACTATTTTGTGTGAAAAAAATTTATCTTCAATTTCATTTCATAATCTTGTTTGATAAGAAAAAAAATCTTGTTGTCTAAGTAAAAAGTAAAATAC>3086
3003>GTAAATAACTATTTTGTGTGAAAAAAATTTATCTTCAATTTCATTTCATAATCTTGTTTGATAAGAAAAAAAATCTTGTTGTCTAAGTAAAAAGTAAAATAC>3102
2959>GTAAATAACTATTTTGTGTGAAAAAAATTTATCTTCAATTTCATTTCATAATCTTGTTTGATAAGAAAAAAAATCTTGTTGTCTAAGTAAAAAGTAAAATAC>3058
2941>GTAAATAACTATTTTGTGTGAAAAAAATTTATCTTCAATTTCATTTCATAATCTTGTTTGATAAGAAAAAAAATCTTGTTGTCTAAGTAAAAAGTAAAATAC>3040
2997>GTATATAACTATTTTGTGTGAAAAAAATTTATCTTCAATTTCATTTCATAATCTTGTTTGATAAGAAAAAAAATCTTGTTGTCTAAGTAAAAAGTAAAATAC>3096

          *         *         *         *         *         *         *         *         *         *
3087>AATTTATTAAATTAATCACCAAAATAGTTCGGTTCTTATTATGTGTGATAATAAAAATACTATTTTCAGGGTGGTTCCTTAACGGTGGATGTTGTAGAAGT>3186
3103>AATTTATTAAATTAATCACCAAAATAGTTCGGTTCTTATTATGTGTGATAATAAAAATACTATTTTCAGGGTGGTTCCTTAACGGTGGATGTTGTAGAAGT>3202
3059>AATTTATTAAATTAATCACCAAAATAGTTCGGTTCTTATTATGTGTGATAATAAAAATACTATTTTCAGGGTGGTTCCTTAACGGTGGATGTTGTAGAAGT>3158
3041>AATTTATTAAATTAATCACCAAAATAGTTCGGTTCTTATTATGTGTGATAATAAAAATACTATTTTCAGGGTGGTTCCTTAACGGTGGATGTTGTAGAAGT>3140
3097>AATTTATTAAATTAATCACCAAAATAGTTCGGTTCTTATTATGTGTGATAATAAAAATACTATTTTCAGGGTGGTTCCTTAACGGTGGATGTTGTAGAAGT>3196

          *         *         *         *         *         *         *         *         *         *
3187>TGAAGGACGACGTGGATTTAGTGATATTGCAGAAAACGCAGAGCGTGTGTTGGTGTGAGGAATGAATGATCAATGTCCCATCAGGAGCAAGCCTTAGTGT>3286
3203>TGAAGGACGACGTGGATTTAGTGATATTGCAGAAAACGCAGAGCGTGTGTTGGTGTGAGGAATGAATGATCAATGTCCCATCAGGAGCAAGCCTTAGTGT>3302
3159>TGAAGGACGACGTGGATTTAGTGATATTGCAGAAAACGCAGAGCGTGTGTTGGTGTGAGGAATGAATGATCAATGTCCCATCAGGAGCAAGCCTTAGTGT>3258
3141>TGAAGGACGACGTGGATTTAGTGATATTGCAGAAAACGCAGAGCGTGTGTTGGTGTGAGGAATGAATGATCAATGTCCCATCAGGAGCAAGCCTTAGTGT>3240
3197>TGAAGGACGACGTGGATTTAGTGATATTGCAGAAAACGCAGAGCGTGTGTTGGTGTGAGGAATGAATGATCAATGTCCCATCAGGAGCAAGCCTTAGTGT>3296

          *         *         *         *         *         *         *         *         *         *
3287>CCCTTCTCTCCCAACTCGCCCTTTCCTTCGACGGCGCCGTTTTGGGCTTCGCCTTGGCCTACGCCGCCATCCGCACCCTCTTCAAATTCTCCGCCACCTC>3386
3303>CCCTTCTCTCCCAACTCGCCCTTTCCTTCGACGGCGCCGTTTTGGGCTTCGCCTtggcctacgccGCCATCCGCACCCTCTTCAAATTCTCCGCCACTTC>3402
3259>CCCTTCTCTCCCAACTCGCCCTTTCCTTCGACGGCGCCGTTTTGGGCTTCGCCTTGGCCTACGCCGCCATCCGCACCCTCTTCAAATTCTCCGCCACTTC>3358
3241>CCCTTCTCTCCCAACTCGCCCTTTCCTTCGACGGCGCCGTTTTGGGCTTCGCCTTGGCCTACGCCGCCATCCGCACCCTCTTCAAATTCTCCGCCACTTC>3340
3297>CCCTTCTCTCCCAACTCGCCCTTTCCTTCGACGGCGCCGTTTTGGGCTTCGCCTTGGCCTACGCCGCCATCCGCACCCTCTTCAAATTCTCCGCCACCTC>3396

          *         *         *         *         *         *         *         *         *         *
3387>TGCCGCCCTCCGCAAGCTCCGCCGCGCACCTTACCTCTCTGTTTCCGACCTCCGATCACTCCTCGCCGATACTCCCTCTGACGCCGACTCTACCTCTGAC>3486
3403>TGCCGCCCTCCGCAAGCTCCGCCGCGCACCTTACCTCTCTGTTTCCGACCTCCGATCACTCCTCGCCGATACTCCCTCTGACGCCGACTCTACCTCTGAC>3502
3359>TGCCGCCCTCCGCAAGCTCCGCCGCGCACCTTACCTCTCTGTTTCCGACCTCCGATCACTCCTCGCCGATACTCCCTCTGACGCCGACTCTACCTCTGAC>3458
3341>TGCCGCCCTCCGCAAGCTCCGCCGCGCACCTTACCTCTCTGTTTCCGACCTCCGATCACTCCTCGCCGATACTCCCTCTGACGCCGACTCTACCTCTGAC>3440
3397>TGCCGCCCTCCGCAAGCTCCGCCGCGCACCTTACCTCTCTGTTTCCGACCTCCGATCACTCCTCGCCGATACTCCCTCTGACGCCGACTCTACCTCTGAC>3496

          *         *         *         *         *         *         *         *         *         *
3487>GGTGGTACAATCGTAATTGTTCGCGGCACCGTTGATGCCAAGTCTGCCGTCGATGGCTCCTGGAAAACCCTCAGACCGGGCGTCTTGGTCTCCCGAGAGT>3586
3503>GGTGGTACAATCGTAATTGTTCGCGGCACCGTTGATGCCAAGTCTGCCGTCGATGGCTCCTGGAAAACCCTCAGACCGGGCGTCTTGGTCTCTCGAGAGT>3602
3459>GGTGGTACAATCGTAATTGTTCGCGGCACCGTTGATGCCAAGTCTGCCGTCGATGGCTCCTGGAAAACCCTCAGACCGGGCGTCTTGGTCTCTCGAGAGT>3558
3441>GGTGGTACAATCGTAATTGTTCGCGGCACCGTTGATGCCAAGTCTGCCGTCGATGGCTCCTGGAAAACCCTCAGACCGGGCGTCTTGGTCTCTCGAGAGT>3540
3497>GGTGGTACAATCGTAATTGTTCGCGGCACCGTTGATGCCAAGTCTGCCGTCGATGGCTCCTGGAAAACCCTCAGACCGGGCGTCTTGGTCTCCCGAGAGT>3596

          *         *         *         *         *         *         *         *         *         *
3587>CCGGTGATAAAGGCGTCATCCTTCAAAGAACTCAAACGGTAATCTCCTTATTTCTTTCTCGAATTTATTGAGATTAATGTGCGATTGAGCAGAGGGATTT>3686
3603>CCGGTGATAAAGGCGTCATCCTTCAAAGAACCCAAACGGTAATCTCCTTATTTCTTTCTCGAGTTTATTGAGATTAATGTGCGATTTAGCAGAGGGATTT>3702
3559>CCGGTGATAAAGGCGTCATCCTTCAAAGAACCCAAACGGTAATCTCCTTATTTCTTTCTCGAGTTTATTGAGATTAATGTGCGATTTAGCAGAGGGATTT>3658
3541>CCGGTGATAAAGGCGTCATCCTTCAAAGAACCCAAACGGTAATCTCCTTATTTCTTTCTCGAGTTTATTGAGATTAATGTGCGATTTAGCAGAGGGATTT>3640
3597>CCGGTGATAAAGGCGTCATCCTTCAAAGAACTCAAACGGTAATCTCCTTATTTCTTTCTCGAATTTATTGAGATTAATGTGCGATTGAGCAGAGGGATTT>3696

          *         *         *         *         *         *         *         *         *         *
3687>GCATGTTTGTTGAAAATTCGCTGTCGTAGAGAGTTTTATTCGAAATTTTGGGGTGTATGTTAAGTGGCTATTAGTGGTTTTAGAACAGTGGTCGACCTGT>3786
3703>GCATGTTTGTTGAAAATTCGCTGTCGTAGAGAGTTTTATTCGAAATTTTGGGGTGTATGTTAAGTGGCTATTAGTGGTTTTAGAACAGTGGTCGACCTGT>3802
3659>GCATGTTTGTTGAAAATTCGCTGTCGTAGAGAGTTTTATTCGAAATTTTGGGGTGTATGTTAAGTGGCTATTAGTGGTTTTAGAACAGTGGTCGACCTGT>3758
3641>GCATGTTTGTTGAAAATTCGCTGTCGTAGAGAGTTTTATTCGAAATTTTGGGGTGTATGTTAAGTGGCTATTAGTGGTTTTAGAACAGTGGTCGACCTGT>3740
3697>GCATGTTTGTTGAAAATTCGCTGTCGTAGAGAGTTTTATTCGAAATTTTGGGGTGTATGTTAAGTGGCTATTAGTGGTTTTAGAACAGTGGTCGACCTGT>3796
```

```
              *         *         *         *         *         *         *         *         *         *
3787>AATTTCGTATGCTTTAATTTAGGAAGTGAAAAGTTGAAATCCTGGAGTACCGTCAGCTCACACTTGATTCAGAACAAAAATTCGTGAAGGTGGAAAGTAA>3886
3803>AATTTCGTATGCTTTAATTTAGGAAGTGAAAAGTTGAAATCCTGGAGTACCGTCAGCTCACACTTGATTCAGAACAAAAATTCGTGAAGGTGGAAAGTAA>3902
3759>AATTTCGTATGCTTTAATTTAGGAAGTGAAAAGTTGAAATCCTGGAGTACCGTCAGCTCACACTTGATTCAGAACAAAAATTCGTGAAGGTGGAAAGTAA>3858
3741>AATTTCGTATGCTTTAATTTAGGAAGTGAAAAGTTGAAATCCTGGAGTACCGTCAGCTCACACTTGATTCAGAACAAAAATTCGTGAAGGTGGAAAGTAA>3840
3797>AATTTCGTATGCTTTAATTTAGGAAGTGAAAAGTTGAAATCCTGGAGTACCGTCAGCTCACACTTGATTCAGAACAAAAATTCGTGAAGGTGGAAAGTAA>3896

              *         *         *         *         *         *         *         *         *         *
3887>AACGGAACTTAGTTTGTTTGTACCTTCAATGTTGGTTGGTTGGTTGAATGTCAATCCGACATTTTCTTTCTTCAACTATCAGTTGCACACTATTAGAGCA>3986
3903>AACGGAACTTAGTTTGTTTGTACCTTCAATGTTGGTTGGTTGGTTGAATGTCAATCCGACATTTTCTTTCTTCAACTATCAGTTGCACACTATTAGAGCA>4002
3859>AACGGAACTTAGTTTGTTTGTACCTTCAATGTTGGTTGGTTGGTTGAATGTCAATCCGACATTTTCTTTCTTCAACTATCAGTTGCACACTATTAGAGCA>3958
3841>AACGGAACTTAGTTTGTTTGTACCTTCAATGTTGGTTGGTTGGTTGAATGTCAATCCGACATTTTCTTTCTTCAACTATCAGTTGCACACTATTAGAGCA>3940
3897>AACGGAACTTAGTTTGTTTGTACCTTCAATGTTGGTTGGTTGGTTGAATGTCAATCCGACATTTTCTTTCTTCAACTATCAGTTGCACACTATTAGAGCA>3996

              *         *         *         *         *         *         *         *         *         *
3987>TGTGTTGTTAAAGAACCAGAATGAACCAAATTGGTAACAATTGAATTGAACTGTAAAATGGACCATGCAACTAAATTGAACTGACTTTGCTTCACTTTGA>4086
4003>TGTGTTGTTAAAGAACCAGAATGAACCAAATTGGTAACAATTGAATTGAACTGTAAAATGGACCATGCAACTAAATTGAACTGACTTTGCTTCACTCTGA>4102
3959>TGTGTTGTTAAAGAACCAGAATGAACCAAATTGGTAACAATTGAATTGAACTGTAAAATGGACCATGCAACTAAATTGAACTGACTTTGCTTCACTCTGA>4058
3941>TGTGTTGTTAAAGAACCAGAATGAACCAAATTGGTAACAATTGAATTGAACTGTAAAATGGACCATGCAACTAAATTGAACTGACTTTGCTTCACTCTGA>4040
3997>TGTGTTGTTAAAGAACCAGAATGAACCAAATTGGTAACAATTGAATTGAACTGTAAAATGGACCATGCAACTAAATTGAACTGACTTTGCTTCACTTTGA>4096

              *         *         *         *         *         *         *         *         *         *
4087>CTGCTTTGAAACGAACAGTCTTGGACTACAATAAACTGATTTTGAAAAAGGAAGTTTAAAGATAGATAACAATGCATATAAGTTTTTTTAATATTAGTTA>4186
4103>CTGCTTTGAAACGAACAGTCTTGGACTACAATAAACTGATTTTGAAAAAGGAAGTTTAAAGATAGATAACAATGCATATAAGTTTTTTTAATATTAGTTA>4202
4059>CTGCTTTGAAACGAACAGTCTTGGACTACAATAAACTGATTTTGAAAAAGGAAGTTTAAAGATAGATAACAATGCATATAAGTTTTTTTAATATTAGTTA>4158
4041>CTGCTTTGAAACGAACAGTCTTGGACTACAATAAACTGATTTTGAAAAAGGAAGTTTAAAGATAGATAACAATGCATATAAGTTTTTTTAATATTAGTTA>4140
4097>CTGCTTTGAAACGAACAGTCTTGGACTACAATAAACTGATTTTGAAAAAGGAAGTTTAAAGATAGATAACAATGCATATAAGTTTTTTTAATATTAGTTA>4196

              *         *         *         *         *         *         *         *         *         *
4187>TTTTCACAATAATCAGGCAAAATGCCCTATACATATATAGTGGTTATTAATATTAAACTGTCCATTGTAGAAGTGTTTATTCGTTATGTCTGCAGAAATG>4286
4203>TTT-CACAATAATCAGGCAAAATGCCCTATACATATATAGTGGTTATTAATATTAAACTGTCCATTGTAGAAGTGTTTATTCGTTATGTCTGCAGAAATG>4301
4159>TTT-CACAATAATCAGGCAAAATGCCCTATACATATATAGTGGTTATTAATATTAAACTGTCCATTGTAGAAGTGTTTATTCGTTATGTCTGCAGAAATG>4257
4141>TTT-CACAATAATCAGGCAAAATGCCCTATACATATATAGTGGTTATTAATATTAAACTGTCCATTGTAGAAGTGTTTATTCGTTATGTCTGCAGAAATG>4239
4197>TTTTCACAATAATCAGGCAAAATGCCCTATACATATATAGTGGTTATTAATATTAAACTGTCCATTGTAGAAGTGTTTATTCGTTATGTCTGCAGAAATG>4296

              *         *         *         *         *         *         *         *         *         *
4287>ACTAGATAATTGGGAGTTTAAAACATCCTATATTAGTTAGTAGAGTTTTCCATCTCCAATAATATGAAATGATGAGACAAGAAGAAAAACTTTTCCTAAA>4386
4302>ACTAGATAATTGGGAGTTTAAAACATCCTATATTAGTTAGTAGAGTTTTCCATCTCCAATAATATGAAATGATGAGACAAGAAGAAAAACTTTTCCTAAA>4401
4258>ACTAGATAATTGGGAGTTTAAAACATCCTATATTAGTTAGTAGAGTTTTCCATCTCCAATAATATGAAATGATGAGACAAGAAGAAAAACTTTTCCTAAA>4357
4240>ACTAGATAATTGGGAGTTTAAAACATCCTATATTAGTTAGTAGAGTTTTCCATCTCCAATAATATGAAATGATGAGACAAGAAGAAAAACTTTTCCTAAA>4339
4297>ACTAGATAATTGGGAGTTTAAAACATCCTATATTAGTTAGTAGAGTTTTCCATCTCCAATAATATGAAATGATGAGACAAGAAGAAAAACTTTTCCTAAA>4396

              *         *         *         *         *         *         *         *         *         *
4387>AGTATTCAACTTCCATATTCCAGAATTACTAGTTAATCAGTAACCAAAAAAAAGCTATTGAATCAAATGGTTTGTAGTTTAAAACAAAAAATAAGTACAAC>4486
4402>AGTATTCAACTTCCATATTCCAGAATTACTAGTTAATCAGTAACCAAAAAAAAGCTATTGAATCAAATGGTTTGTAGTTTAAAACAAAAAATAAGTACAAC>4501
4358>AGTATTCAACTTCCATATTCCAGAATTACTAGTTAATCAGTAACCAAAAAAAAGCTATTGAATCAAATGGTTTGTAGTTTAAAACAAAAAATAAGTACAAC>4457
4340>AGTATTCAACTTCCATATTCCAGAATTACTAGTTAATCAGTAACCAAAAAAAAGCTATTGAATCAAATGGTTTGTAGTTTAAAACAAAAAATAAGTACAAC>4439
4397>AGTATTCAACTTCCATATTCCAGAATTACTAGTTAATCAGTAACCAAAAAAAAGCTATTGAATCAAATGGTTTGTAGTTTAAAACAAAAAATAAGTACAAC>4496

              *         *         *         *         *         *         *         *         *         *
4487>GGTATTGCATATATCCACATAAAAAATAGATTTAATAGAAACACGGCAAAGTAAGTGGGAGAACCAAATAAAAAGTGGTTAATTCATTTGAGCATATTTGT>4586
4502>GGTATTGCATATATCCACATAAAAAATAGATTTAATAGAAACACGGCAA-GTAAGTGGGAGAACCAAATAAAAAGTGGTTAATTCATTTGAGCATATTTGT>4600
4458>GGTATTGCATATATCCACATAAAAAATAGATTTAATAGAAACACGGCAA-GTAAGTGGGAGAACCAAATAAAAAGTGGTTAATTCATTTGAGCATATTTGT>4556
4440>GGTATTGCATATATCCACATAAAAAATAGATTTAATAGAAACACGGCAA-GTAAGTGGGAGAACCAAATAAAAAGTGGTTAATTCATTTGAGCATATTTGT>4538
4497>GGTATTGCATATATCCACATAAAAAATAGATTTAATAGAAACACGGCAAAGTAAGTGGGAGAACCAAATAAAAAGTGGTTAATTCATTTGAGCATATTTGT>4596

              *         *         *         *         *         *         *         *         *         *
4587>TTATTTTTCTTTACTTGAAATTACACATCATAAGTTGGACACCAGGTCATGAACGAGGGTGCTTTACAAAAATCAGTGGAAATTCTTTGAAACCAACAAG>4686
4601>TTATTTTTCTTTACTTGAAATTACACATCATAAGTTGGACACCAGGTCATGAACGAGGGTGCTTTACAAAAATCAGTGGAAATTCTTTGAAACCAACAAG>4700
4557>TTATTTTTCTTTACTTGAAATTACACATCATAAGTTGGACACCAGGTCATGAACGAGGGTGCTTTACAAAAATCAGTGGAAATTCTTTGAAACCAACAAG>4656
4539>TTATTTTTCTTTACTTGAAATTACACATCATAAGTTGGACACCAGGTCATGAACGAGGGTGCTTTACAAAAATCAGTGGAAATTCTTTGAAACCAACAAG>4638
4597>TTATTTTTCTTTACTTGAAATTACACATCATAAGTTGGACACCAGGTCATGAACGAGGGTGCTTTACAAAAATCAGTGGAAATTCTTTGAAACCAACAAG>4696

              *         *         *         *         *         *         *         *         *         *
4687>ATAAACTACTTGCTTCTAGCTCTGAACTTTTTCTATAACAGTTCACTTCAGTATTAAAACTGCTTTTGAAAGAGTTTAGATTCAATTTATTTTAAAACAG>4786
4701>ATAAACTACTTGCTTCTAGCTCTGAACTTTTTCTATAACAGTTCACTTCAGTATTAAAACTGATTTTGAAAGAGTTTAGATTCAATTTATTTTAAAACAG>4800
4657>ATAAACTACTTGCTTCTAGCTCTGAACTTTTTCTATAACAGTTCACTTCAGTATTAAAACTGATTTTGAAAGAGTTTAGATTCAATTTATTTTAAAACAG>4756
4639>ATAAACTACTTGCTTCTAGCTCTGAACTTTTTCTATAACAGTTCACTTCAGTATTAAAACTGATTTTGAAAGAGTTTAGATTCAATTTATTTTAAAACAG>4738
4697>ATAAACTACTTGCTTCTAGCTCTGAACTTTTTCTATAACAGTTCACTTCAGTATTAAAACTGCTTTTGAAAGAGTTTAGATTCAATTTATTTTAAAACAG>4796
```

```
                  *         *         *         *         *         *         *         *         *         *
4787>TTTAGTTATTTTGAAGTTAATTTAGTTCGGTTATTAAAGTTAGTTTTAGATTAGTTACTTTTGGACAATCTTACATTTTGGATTGGCATTCGGCACAGTG>4886
4801>TTTAGTTATTTTGAAGTTAATTTAGTTCGGTTATTAAAGTTAGTTTTAGATTAGTTACTTTTGGACAATCTTACATTTTGGATTGGCATTCGGCACAGTG>4900
4757>TTTAGTTATTTTGAAGTTAATTTAGTTCGGTTATTAAAGTTAGTTTTAGATTAGTTACTTTTGGACAATCTTACATTTTGGATTGGCATTCGGCACAGTG>4856
4739>TTTAGTTATTTTGAAGTTAATTTAGTTCGGTTATTAAAGTTAGTTTTAGATTAGTTACTTTTGGACAATCTTACATTTTGGATTGGCATTCGGCACAGTG>4838
4797>TTTAGTTATTTTGAAGTTAATTTAGTTCGGTTATTAAAGTTAGTTTTAGATTAGTTACTTTTGGACAATCTTACATTTTGGATTGGCATTCGGCACAGTG>4896

                  *         *         *         *         *         *         *         *         *         *
4887>AAAGGGTTTATCAAGCACATCATCA-TTGTGTCCTACTTTTCAGAAACGTGAGTCCATCTCTCATAGTTTGTTGGTGTTTAGCGTGGCTCAATGACTTTT>4985
4901>AAAGGGTTTATCAAGCACATCATCAATTGTGTCCTACTTTTCAGAAACGTGAGTCCATCTCTCATAGTTTGTTGGTGTTTAGCGTGGCTCAATGACTTTT>5000
4857>AAAGGGTTTATCAAGCACATCATCAATTGTGTCCTACTTTTCAGAAACGTGAGTCCATCTCTCATAGTTTGTTGGTGTTTAGCGTGGCTCAATGACTTTT>4956
4839>AAAGGGTTTATCAAGCACATCATCAATTGTGTCCTACTTTTCAGAAACGTGAGTCCATCTCTCATAGTTTGTTGGTGTTTAGCGTGGCTCAATGACTTTT>4938
4897>AAAGGGTTTATCAAGCACATCATCA-TTGTGTCCTACTTTTCAGAAACGTGAGTCCATCTCTCATAGTTTGTTGGTGTTTAGCGTGGCTCAATGACTTTT>4995

                  *         *         *         *         *         *         *         *         *         *
4986>CGAACACATGATTACTTCCACCACCTTAACCCAAGCCCTCAGTATGTGTGTATGTATGTATAACTGCACGTCAAACACACTATATGCGTATCCTAAACTA>5085
5001>CGAACACACGATTACTTCCACCACCTTAACCCAAGCCCTCAGTATGTGT--ATGTATGTATAACTGCACGTCAAACACACTATATGCGTATCCTAAACTA>5098
4957>CGAACACACGATTACTTCCACCACCTTAACCCAAGCCCTCAGTATGTGT--ATGTATGTATAACTGCACGTCAAACACACTATATGCGTATCCTAAACTA>5054
4939>CGAACACACGATTACTTCCACCACCTTAACCCAAGCCCTCAGTATGTGT--ATGTATGTATAACTGCACGTCAAACACACTATATGCGTATCCTAAACTA>5036
4996>CGAACACATGATTACTTCCACCACCTTAACCCAAGCCCTCAGTATGTGTGTATGTATGTATAACTGCACGTCAAACACACTATATGCGTATCCTAAACTA>5095

                  *         *         *         *         *         *         *         *         *         *
5086>CAAAGTTGTTATAATCTAACTGTTATAATTATCTATTATATCAATAATTGCACTTCGTCTTCTAAAGTATGACTTCAGATTGTTACCAAAGAGTTCCCTG>5185
5099>CAAAGTTGTTATAATCTAATTGTTATAATTATCTATTATATCAATAATTGCACTTCGTCTTCTAAAGTATGACTTCAGATTGTTACCTAAGAGTTCCCTG>5198
5055>CAAAGTTGTTATAATCTAATTGTTATAATTATCTATTATATCAATAATTGCACTTCGTCTTCTAAAGTATGACTTCAGATTGTTACCTAAGAGTTCCCTG>5154
5037>CAAAGTTGTTATAATCTAATTGTTATAATTATCTATTATATCAATAATTGCACTTCGTCTTCTAAAGTATGACTTCAGATTGTTACCTAAGAGTTCCCTG>5136
5096>CAAAGTTGTTATAATCTAACTGTTATAATTATCTATTATATCAATAATTGCACTTCGTCTTCTAAAGTATGACTTCAGATTGTTACCAAAGAGTTCCCTG>5195

                  *         *         *         *         *         *         *         *         *         *
5186>TTCAATTTATGGATAGTGTACTTTTTGATGTTTTGGCCATAAGATGTTCTTTGATATTAAGT-TTGACATGCCTCCTCTTGTTCTTGATTTATTTATATG>5284
5199>TTCAATTTATGGATAGTGTACTTTTTGATGTTTTGGCCATAAGATGTTCTTTGATATTAAGTATTGACATGCCTCCTATTGTTCTTGATTTATTTATATG>5298
5155>TTCAATTTATGGATAGTGTACTTTTTGATGTTTTGGCCATAAGATGTTCTTTGATATTAAGTATTGACATGCCTCCTATTGTTCTTGATTTATTTATATG>5254
5137>TTCAATTTATGGATAGTGTACTTTTTGATGTTTTGGCCATAAGATGTTCTTTGATATTAAGTATTGACATGCCTCCTATTGTTCTTGATTTATTTATATG>5236
5196>TTCAATTTATGGATAGTGTACTTTTTGATGTTTTGGCCATAAGATGTTCTTTGATATTAAGT-TTGACATGCCTCCTCTTGTTCTTGATTTATTTATATG>5294

                  *         *         *         *         *         *         *         *         *         *
5285>AACAACTTTTATGTCTTAAAGGAATAATATCTTTTTCGTTGTTGTAGAAAATATAAATTTAGTTTCAGCTTTAATGTCTTGCAATTTAGTAAGTTATTTG>5384
5299>AACAACTTTTATGTCTTAAAGGAATAATATCTTTTTCGTTGTTGTAGAAAATATAAATTTAGTTTCAGCTTTAATGTCTTGCAATTTAGTAAGTTATTTG>5398
5255>AACAACTTTTATGTCTTAAAGGAATAATATCTTTTTCGTTGTTGTAGAAAATATAAATTTAGTTTCAGCTTTAATGTCTTGCAATTTAGTAAGTTATTTG>5354
5237>AACAACTTTTATGTCTTAAAGGAATAATATCTTTTTCGTTGTTGTAGAAAATATAAATTTAGTTTCAGCTTTAATGTCTTGCAATTTAGTAAGTTATTTG>5336
5295>AACAACTTTTATGTCTTAAAGGAATAATATCTTTTTCGTTGTTGTAGAAAATATAAATTTAGTTTCAGCTTTAATGTCTTGCAATTTAGTAAGTTATTTG>5394

                  *         *         *         *         *         *         *         *         *         *
5385>TGAATTAGAGGTCTAATTTATTTATTTGTTTTGTGTTGATTATAATGAAGTGTATATACAATGAATGGAAAGGATTATTTGGATGGACTTCTGATCTTCG>5484
5399>TGAATTAGAGGTCTAATTTATTTATTTGTTTTGTGTTGATTATAATGAAGTGTATATACAATGAATGGAAAGGATTATTTGGATGGACTTCTGATCTTCG>5498
5355>TGAATTAGAGGTCTAATTTATTTATTTGTTTTGTGTTGATTATAATGAAGTGTATATACAATGAATGGAAAGGATTATTTGGATGGACTTCTGATCTTCG>5454
5337>TGAATTAGAGGTCTAATTTATTTATTTGTTTTGTGTTGATTATAATGAAGTGTATATACAATGAATGGAAAGGATTATTTGGATGGACTTCTGATCTTCG>5436
5395>TGAATTAGAGGTCTAATTTATTTATTTGTTTTGTGTTGATTATAATGAAGTGTATATACAATGAATGGAAAGGATTATTTGGATGGACTTCTGATCTTCG>5494

                  *         *         *         *         *         *         *         *         *         *
5485>GGCCATATTTGCAAGATCTTGGAGACAGCAAGAGTCTACATCTTTAAGGAAGGTAATGGGTGCATAACTTTGTTACATATATTGAGGTTCATAATGTATA>5584
5499>GGCCATATTTGCAAGATCTTGGAGACAGCAAGAGTCTACATCTTTAAGGAAGGTAATGGGTGCATAACTTTGTTACATATATTGAGGTTCATAATGTATA>5598
5455>GGCCATATTTGCAAGATCTTGGAGACAGCAAGAGTCTACATCTTTAAGGAAGGTAATGGGTGCATAACTTTGTTACATATATTGAGGTTCATAATGTATA>5554
5437>GGCCATATTTGCAAGATCTTGGAGACAGCAAGAGTCTACATCTTTAAGGAAGGTAATGGGTGCATAACTTTGTTACATATATTGAGGTTCATAATGTATA>5536
5495>GGCCATATTTGCAAGATCTTGGAGACAGCAAGAGTCTACATCTTTAAGGAAGGTAATGGGTGCATAACTTTGTTACATATATTGAGGTTCATAATGTATA>5594

                  *         *         *         *         *         *         *         *         *         *
5585>GAGAGAATTCTGGTATGATATGGTCAATTGATTTGTATTTCATGAGGTCAAATCTGATACCAGTTGGTATATATAATTGAGTGGGGTTGACAACAACTCT>5684
5599>GAGAGAATTCTGGTATGATATGTTCAATTGATTTGTATTTCATGAGGTCAAATCTGATACCAGTTGGTATATATAATTGAGTGGGGTTGACAACAACTCT>5698
5555>GAGAGAATTCTGGTATGATATGTTCAATTGATTTGTATTTCATGAGGTCAAATCTGATACCAGTTGGTATATATAATTGAGTGGGGTTGACAACAACTCT>5654
5537>GAGAGAATTCTGGTATGATATGTTCAATTGATTTGTATTTCATGAGGTCAAATCTGATACCAGTTGGTATATATAATTGAGTGGGGTTGACAACAACTCT>5636
5595>GAGAGAATTCTGGTATGATATGGTCAATTGATTTGTATTTCATGAGGTCAAATCTGATACCAGTTGGTATATATAATTGAGTGGGGTTGACAACAACTCT>5694

                  *         *         *         *         *         *         *         *         *         *
5685>GTATCCTAGTAGATTATTTTATATCCATAATCAATTAGTGGTTTTATTTTATTTTTTTAATTATATATAATATATAGTACTATCTGTTGCTGCAAAAGAT>5784
5699>CTATCCTAGTAGATTATTTTATATCCATAATCAATTAGTGGTTTTATTTTATTTTTTTAATTATATATAATATATAGTACTATCTGTTGCTGCAAAAGAT>5798
5655>CTATCCTAGTAGATTATTTTATATCCATAATCAATTAGTGGTTTTATTTTATTTTTTTAATTATATATAATATATAGTACTATCTGTTGCTGCAAAAGAT>5754
5637>CTATCCTAGTAGATTATTTTATATCCATAATCAATTAGTGGTTTTATTTTATTTTTTTAATTATATATAATATATAGTACTATCTGTTGCTGCAAAAGAT>5736
5695>GTATCCTAGTAGATTATTTTATATCCATAATCAATTAGTGGTTTTATTTTATTTTTTTAATTATATATAATATATAGTACTATCTGTTGCTGCAAAAGAT>5794
```

```
              *         *         *         *         *         *         *         *         *         *
5785>TGTGCACCATGGAGATGCTTCAGCTGATCTTGGCCTAGGTTAGTAAAAGCAGCTCAGCATTTCAAACAGGATGCACTTTCAGTTGCAATGTCTGGAACAC>5884
5799>TGTGCACCATGGAGATGCTTCAGCTGATCTTGGCCTGGGTTAGTAAAAGCAGCTCAGCATTTCAAACAGGATGCACTTTCAGTTGCAATGTCTGGAACAC>5898
5755>TGTGCACCATGGAGATGCTTCAGCTGATCTTGGCCTGGGTTAGTAAAAGCAGCTCAGCATTTCAAACAGGATGCACTTTCAGTTGCAATGTCTGGAACAC>5854
5737>TGTGCACCATGGAGATGCTTCAGCTGATCTTGGCCTGGGTTAGTAAAAGCAGCTCAGCATTTCAAACAGGATGCACTTTCAGTTGCAATGTCTGGAACAC>5836
5795>TGTGCACCATGGAGATGCTTCAGCTGATCTTGGCCTAGGTTAGTAAAAGCAGCTCAGCATTTCAAACAGGATGCACTTTCAGTTGCAATGTCTGGAACAC>5894

              *         *         *         *         *         *         *         *         *         *
5885>GAGCTAATGAATAGTAATAAAGGAATTTGATCATAGTTAAATTACTGCAGTTAACAAGTACCAAGTTACTCCACTCCTGTTTTTCAAGCTGCAATGTGAG>5984
5899>GAGCTAATGAATAGTAATAAAGGAATTTGATCATAGTTAAATTACTGCAGTTAACAAGTACCAAGTTACTCCACTCCTGTTTTTCAAGCTGCAATGTGAG>5998
5855>GAGCTAATGAATAGTAATAAAGGAATTTGATCATAGTTAAATTACTGCAGTTAACAAGTACCAAGTTACTCCACTCCTGTTTTTCAAGCTGCAATGTGAG>5954
5837>GAGCTAATGAATAGTAATAAAGGAATTTGATCATAGTTAAATTACTGCAGTTAACAAGTACCAAGTTACTCCACTCCTGTTTTTCAAGCTGCAATGTGAG>5936
5895>GAGCTAATGAATAGTAATAAAGGAATTTGATCATAGTTAAATTACTGCAGTTAACAAGTACCAAGTTACTCCACTCCTGTTTTTCAAGCTGCAATGTGAG>5994

              *         *         *         *         *         *         *         *         *         *
5985>AGGTTGGAGAGGTAGAGATACAGCTATATCAATTTCCTGCCAGTATGCCCCTATCCCCTGCCCCATAAAAAAAGGATATGAATGCTTTTTAGTTTGCATT>6084
5999>AGGTTGGAGAGGTAGAGATACAGCTATATCAATTTCCTGCCAGTATGCTCCTATCCCCTGCCCCATAAAAAAAGGATATGAATGCTTTTTAGTTTGCATT>6098
5955>AGGTTGGAGAGGTAGAGATACAGCTATATCAATTTCCTGCCAGTATGCTCCTATCCCCTGCCCCATAAAAAAAGGATATGAATGCTTTTTAGTTTGCATT>6054
5937>AGGTTGGAGAGGTAGAGATACAGCTATATCAATTTCCTGCCAGTATGCTCCTATCCCCTGCCCCATAAAAAAAGGATATGAATGCTTTTTAGTTTGCATT>6036
5995>AGGTTGGAGAGGTAGAGATACAGCTATATCAATTTCCTGCCAGTATGCCCCTATCCCCTGCCCCATAAAAAAAGGATATGAATGCTTTTTAGTTTGCATT>6094

              *         *         *         *         *         *         *         *         *         *
6085>TATTTTGTGTTTTGAAACTGATTTTAATGTGAAAAGTCTGTATGTGGATTTTATTAGATGATAGAAAAAAGATAGAAAGTTACCAAAGGAATAAAAAGGA>6184
6099>TATTTTGTGTTTTGAAACTGATTTTAATGTGAAAAGTCTGTATGTGGATTTTATTAGATGATAGAAAAAAGATAGAAAGTTACCAAAGGAATAAAAAAGA>6198
6055>TATTTTGTGTTTTGAAACTGATTTTAATGTGAAAAGTCTGTATGTGGATTTTATTAGATGATAGAAAAAAGATAGAAAGTTACCAAAGGAATAAAAAAGA>6154
6037>TATTTTGTGTTTTGAAACTGATTTTAATGTGAAAAGTCTGTATGTGGATTTTATTAGATGATAGAAAAAAGATAGAAAGTTACCAAAGGAATAAAAAAGA>6136
6095>TATTTTGTGTTTTGAAACTGATTTTAATGTGAAAAGTCTGTATGTGGATTTTATTAGATGATAGAAAAAAGATAGAAAGTTACCAAAGGAATAAAAAGGA>6194

              *         *         *         *         *         *         *         *         *         *
6185>GAATAAAAACAGATGGATAGTTGAACTAGAACATAGTAACAAGAAAAAGTAAACGAAAGAAAAGAAGATAGATTGACAGAAAGAAAGTTATAGCACAGAG>6284
6199>GAATAAAAACAGATGGATAGTTGAACTAGAACATAGTAACAAGAAAAAGTAAACGAAAGAAAAGAAGATAGATTCACAGAAAGAAAGTTATAGCACAGAG>6298
6155>GAATAAAAACAGATGGATAGTTGAACTAGAACATAGTAACAAGAAAAAGTAAACGAAAGAAAAGAAGATAGATTCACAGAAAGAAAGTTATAGCACAGAG>6254
6137>GAATAAAAACAGATGGATAGTTGAACTAGAACATAGTAACAAGAAAAAGTAAACGAAAGAAAAGAAGATAGATTCACAGAAAGAAAGTTATAGCACAGAG>6236
6195>GAATAAAAACAGATGGATAGTTGAACTAGAACATAGTAACAAGAAAAAGTAAACGAAAGAAAAGAAGATAGATTGACAGAAAGAAAGTTATAGCACAGAG>6294

              *         *         *         *         *         *         *         *         *         *
6285>GAAGCTGCAAAGAAGCAAGCTTGGCCTGTGGGCTACTAGGATTTGACTGGAAACTGGATGGGCTGAGGTTTGGAAAAAGATTAGCCTCCACTTGATGGTA>6384
6299>GAAGCTGCAAAGAAGCAAGCTTGGCCTGTGGGCTACTAGGATTTGACTGGAAACTGGATGGGCTGAGGTTTGGAAAAAGATTAGCCTCCACTTGATGGTA>6398
6255>GAAGCTGCAAAGAAGCAAGCTTGGCCTGTGGGCTACTAGGATTTGACTGGAAACTGGATGGGCTGAGGTTTGGAAAAAGATTAGCCTCCACTTGATGGTA>6354
6237>GAAGCTGCAAAGAAGCAAGCTTGGCCTGTGGGCTACTAGGATTTGACTGGAAACTGGATGGGCTGAGGTTTGGAAAAAGATTAGCCTCCACTTGATGGTA>6336
6295>GAAGCTGCAAAGAAGCAAGCTTGGCCTGTGGGCTACTAGGATTTGACTGGAAACTGGATGGGCTGAGGTTTGGAAAAAGATTAGCCTCCACTTGATGGTA>6394

              *         *         *         *         *         *         *         *         *         *
6385>TAATAGTAGGGCATTGATCTGCAATTCTGCTCATATTCAGCCGTGATTTTGGTCAGTATGCATTTGACACGGAGACACAGTGCTGTGAGTTTCTTGTTGG>6484
6399>TAATAGTAGGGCATTGATCTGCAATTCTGCTCATATTCAGCCGTGATTTTGGTCAGTATGCATTTGACACGGAGACACAGTGCTGTGAGTTTCTTGTTGT>6498
6355>TAATAGTAGGGCATTGATCTGCAATTCTGCTCATATTCAGCCGTGATTTTGGTCAGTATGCATTTGACACGGAGACACAGTGCTGTGAGTTTCTTGTTGT>6454
6337>TAATAGTAGGGCATTGATCTGCAATTCTGCTCATATTCAGCCGTGATTTTGGTCAGTATGCATTTGACACGGAGACACAGTGCTGTGAGTTTCTTGTTGT>6436
6395>TAATAGTAGGGCATTGATCTGCAATTCTGCTCATATTCAGCCGTGATTTTGGTCAGTATGCATTTGACACGGAGACACAGTGCTGTGAGTTTCTTGTTGG>6494

              *         *         *         *         *         *         *         *         *         *
6485>GGCTAGGGGCCAGGACTCTGTGACAACATCTTTTTGACACTATGTTGTTGAGCCTTTCAAGGGAGATTCAACACTAATAAACCATGAGACAACCATATAA>6584
6499>GGCTAGGGGCCAGGACTCTGTGACAACATCTCTTTGACACACTGTTGTTGAGCCTTTCAAGGGAGATTCAACACTAATAAACCATGAGACAACCATATAA>6598
6455>GGCTAGGGGCCAGGACTCTGTGACAACATCTCTTTGACACACTGTTGTTGAGCCTTTCAAGGGAGATTCAACACTAATAAACCATGAGACAACCATATAA>6554
6437>GGCTAGGGGCCAGGACTCTGTGACAACATCTCTTTGACACACTGTTGTTGAGCCTTTCAAGGGAGATTCAACACTAATAAACCATGAGACAACCATATAA>6536
6495>GGCTAGGGGCCAGGACTCTGTGACAACATCTTTTTGACACTATGTTGTTGAGCCTTTCAAGGGAGATTCAACACTAATAAACCATGAGACAACCATATAA>6594

              *         *         *         *         *         *         *         *         *         *
6585>GAAACTACCACACAATCTTCTATCTAATACTTTAAAGACAACGCGTTTGTGGGTCTTTTATGTCCTTCAACATTTTTATTTCTACTCAACGCAAGAATCA>6684
6599>GAAACTACCACACAATCTTCTATCTAATACTTTAAAGACAACGCGTTTGTGGGTCTTTTATGTCGTTCAACTTTTTTATTTCTACTCAACGCAAGAATCA>6698
6555>GAAACTACCACACAATCTTCTATCTAATACTTTAAAGACAACGCGTTTGTGGGTCTTTTATGTCGTTCAACTTTTTTATTTCTACTCAACGCAAGAATCA>6654
6537>GAAACTACCACACAATCTTCTATCTAATACTTTAAAGACAACGCGTTTGTGGGTCTTTTATGTCGTTCAACTTTTTTATTTCTACTCAACGCAAGAATCA>6636
6595>GAAACTACCACACAATCTTCTATCTAATACTTTAAAGACAACGCGTTTGTGGGTCTTTTATGTCCTTCAACATTTTTATTTCTACTCAACGCAAGAATCA>6694

              *         *                         *         *         *         *         *         *
6685>TACTTACACTTAAATTCTTAA----------------TAGATGTTGCCGTTGGATACTCTGTATGGATTGATTATGATGTGTCTTTTGGTTTATCTTTTC>6768
6699>TAGTTACACTTAAATTCTTAATAGATGTTATTCTTAATAGATGTTGCCGTTGGATACTCTGTATGGATTGATTATGATGTGTCTTTTGGTTTATCTTTTC>6798
6655>TAGTTACACTTAAATTCTTAATAGATGTTATTCTTAATAGATGTTGCCGTTGGATACTCTGTATGGATTGATTATGATGTGTCTTTTGGTTTATCTTTTC>6754
6637>TAGTTACACTTAAATTCTTAATAGATGTTATTCTTAATAGATGTTGCCGTTGGATACTCTGTATGGATTGATTATGATGTGTCTTTTGGTTTATCTTTTC>6736
6695>TACTTACACTTAAATTCTTAA----------------TAGATGTTGCCGTTGGATACTCTGTATGGATTGATTATGATGTGTCTTTTGGTTTATCTTTTC>6778
```

```
              *         *         *         *         *         *         *         *         *         *
6769>TTTGTTCCACCTTTGATCTTAATTTGCTTTAATAGCTTGCACATATAATGGGTGAGTTTGTTTGTGCTTATTTTTTGAAATGAGAGTATTTTGAATAAAA>6868
6799>TTTGTTCCACCTTTGATCTTAATTTGCTTTAATAGCTTGCACATATAATGGGTGAGTTTGTTTGTGCTTATTTTTTGAAATGAGAGTATTTTGAATAAAA>6898
6755>TTTGTTCCACCTTTGATCTTAATTTGCTTTAATAGCTTGCACATATAATGGGTGAGTTTGTTTGTGCTTATTTTTTGAAATGAGAGTATTTTGAATAAAA>6854
6737>TTTGTTCCACCTTTGATCTTAATTTGCTTTAATAGCTTGCACATATAATGGGTGAGTTTGTTTGTGCTTATTTTTTGAAATGAGAGTATTTTGAATAAAA>6836
6779>TTTGTTCCACCTTTGATCTTAATTTGCTTTAATAGCTTGCACATATAATGGGTGAGTTTGTTTGTGCTTATTTTTTGAAATGAGAGTATTTTGAATAAAA>6878

              *         *         *         *         *         *         *         *         *         *
6869>TAAGTATATTTCTGCTTTCTTTATGTGTTTGTCTAATCTGCTTAGTTAAAAAATTAGCAGCTTTCTATCTGATTCTCAAATAAGTGATTATTAAGAAACG>6968
6899>TAAGTATATTTCTGCTTTCTTTATGTGTTTGTCTAATCTGCTTAGTTAAAAAATAAGCAGCTTTCTATCTGATTCTCAAATAAGTGATTATTAAGAAACG>6998
6855>TAAGTATATTTCTGCTTTCTTTATGTGTTTGTCTAATCTGCTTAGTTAAAAAATAAGCAGCTTTCTATCTGATTCTCAAATAAGTGATTATTAAGAAACG>6954
6837>TAAGTATATTTCTGCTTTCTTTATGTGTTTGTCTAATCTGCTTAGTTAAAAAATAAGCAGCTTTCTATCTGATTCTCAAATAAGTGATTATTAAGAAACG>6936
6879>TAAGTATATTTCTGCTTTCTTTATGTGTTTGTCTAATCTGCTTAGTTAAAAAATTAGCAGCTTTCTATCTGATTCTCAAATAAGTGATTATTAAGAAACG>6978

              *         *             *         *         *         *         *         *         *         *
6969>GCTTATTTAAAAAACACTT----TAAGTGTAAACAAACTGGCCCATTATATATTCAAATTACAATGCATTGGAGGAAAAAAGAGTTCAAGCTCAAAATTA>7064
6999>GCTTATTTAAAAAACACTTACTTTAAGTGTAAACAAACCGGCCCATTATATATTCAAATTACAATGCATTGGAGGAAAAAAGAGTTCAAGCTCAAAATTA>7098
6955>GCTTATTTAAAAAACACTTACTTTAAGTGTAAACAAACCGGCCCATTATATATTCAAATTACAATGCATTGGAGGAAAAAAGAGTTCAAGCTCAAAATTA>7054
6937>GCTTATTTAAAAAACACTTACTTTAAGTGTAAACAAACCGGCCCATTATATATTCAAATTACAATGCATTGGAGGAAAAAAGAGTTCAAGCTCAAAATTA>7036
6979>GCTTATTTAAAAAACACTT----TAAGTGTAAACAAACTGGCCCATTATATATTCAAATTACAATGCATTGGAGGAAAAAAGAGTTCAAGCTCAAAATTA>7074

                  *         *         *         *         *         *         *         *         *         *
7065>AAAAAAAAATTGAATATTAATTTACTTTATGTATTGTAATGGTGCTTTACAAAATAAAAAGCATTAAGTTTTGTTTACACTGATACTAGACTCATAATGT>7164
7099>-AAAAAAAATTGAATATTAATTTACTTTATGTATTGTAATGGTGCTTTACAAAATAAAAAGCATTAAGTTTTGTTTACACTGATACTAGACTCATAATGT>7197
7055>-AAAAAAAATTGAATATTAATTTACTTTATGTATTGTAATGGTGCTTTACAAAATAAAAAGCATTAAGTTTTGTTTACACTGATACTAGACTCATAATGT>7153
7037>-AAAAAAAATTGAATATTAATTTACTTTATGTATTGTAATGGTGCTTTACAAAATAAAAAGCATTAAGTTTTGTTTACACTGATACTAGACTCATAATGT>7135
7075>AAAAAAAAATTGAATATTAATTTACTTTATGTATTGTAATGGTGCTTTACAAAATAAAAAGCATTAAGTTTTGTTTACACTGATACTAGACTCATAATGT>7174

                  *         *         *         *         *         *         *         *         *         *
7165>TACCAAAATAAGGAGCAAATCATGAAATATATGTATTGAAATATGAAAATCATTCTTTAAGGATCATATAACAATAAAAATAACAACTAAAGTCCTGCCC>7264
7198>TACCAAAATAAGGAGCAAATCATGAAATATATGTATTGAAATATGAAAATCATTCTTTAAGGATCATATAACAATAAAAATAACAACTAAAGTCCTGCCC>7297
7154>TACCAAAATAAGGAGCAAATCATGAAATATATGTATTGAAATATGAAAATCATTCTTTAAGGATCATATAACAATAAAAATAACAACTAAAGTCCTGCCC>7253
7136>TACCAAAATAAGGAGCAAATCATGAAATATATGTATTGAAATATGAAAATCATTCTTTAAGGATCATATAACAATAAAAATAACAACTAAAGTCCTGCCC>7235
7175>TACCAAAATAAGGAGCAAATCATGAAATATATGTATTGAAATATGAAAATCATTCTTTAAGGATCATATAACAATAAAAATAACAACTAAAGTCCTGCCC>7274

                  *         *         *         *         *         *         *         *         *         *
7265>CACTAAGAAAGAATCATGTAAGATATTGTAAATAGTTTTAGATACAATAGTATGAAATGTATTATAGATATTCTGACTATCTTTGTTGTAGGTGCCTTTT>7364
7298>CACTAAGAAAGAATCATGTAAGATATTGTAAATAGTTTTAGATACAATAGTATGAAATGTATTATAGATATTCTGACTATCTTTGTTGTAGGTGCCTTTT>7397
7254>CACTAAGAAAGAATCATGTAAGATATTGTAAATAGTTTTAGATACAATAGTATGAAATGTATTATAGATATTCTGACTATCTTTGTTGTAGGTGCCTTTT>7353
7236>CACTAAGAAAGAATCATGTAAGATATTGTAAATAGTTTTAGATACAATAGTATGAAATGTATTATAGATATTCTGACTATCTTTGTTGTAGGTGCCTTTT>7335
7275>CACTAAGAAAGAATCATGTAAGATATTGTAAATAGTTTTAGATACAATAGTATGAAATGTATTATAGATATTCTGACTATCTTTGTTGTAGGTGCCTTTT>7374

                  *         *         *         *         *         *         *         *         *         *
7365>GTTCTAATTGATGTTGGACGGCGACCATCTACTCCTGAGTATGTTGTTGTCAACATGGATGGCTCAAGGCATCCATTGCCTCTGACAACGGTTTATCATA>7464
7398>GTTCTAATTGATGTTGGACGGCGACCATCTACTCTTGAGTATGTTGTTGTCAACATGGATGGCTCAAGGCATCCATTGCCTCTGACAACGGTTTATCATA>7497
7354>GTTCTAATTGATGTTGGACGGCGACCATCTACTCTTGAGTATGTTGTTGTCAACATGGATGGCTCAAGGCATCCATTGCCTCTGACAACGGTTTATCATA>7453
7336>GTTCTAATTGATGTTGGACGGCGACCATCTACTCTTGAGTATGTTGTTGTCAACATGGATGGCTCAAGGCATCCATTGCCTCTGACAACGGTTTATCATA>7435
7375>GTTCTAATTGATGTTGGACGGCGACCATCTACTCCTGAGTATGTTGTTGTCAACATGGATGGCTCAAGGCATCCATTGCCTCTGACAACGGTTTATCATA>7474

                  *         *         *         *         *         *         *         *         *         *
7465>AATTGCAACCAATAAATGCTTCTCCTTATACTTTCTTGCAAGCACTTTTTGGGCATGAATATCCGGTGAGTTGATATTCTCATTACTTATTTGCCGTGGA>7564
7498>AATTGCAACCAATAAATGCTTCTCCTTATACTTTCTTGCAAGCACTTTTTGGGCATGAATATCCGGTGAGTTGATATTCTCATTACTTATTTGCCGTGGA>7597
7454>AATTGCAACCAATAAATGCTTCTCCTTATACTTTCTTGCAAGCACTTTTTGGGCATGAATATCCGGTGAGTTGATATTCTCATTACTTATTTGCCGTGGA>7553
7436>AATTGCAACCAATAAATGCTTCTCCTTATACTTTCTTGCAAGCACTTTTTGGGCATGAATATCCGGTGAGTTGATATTCTCATTACTTATTTGCCGTGGA>7535
7475>AATTGCAACCAATAAATGCTTCTCCTTATACTTTCTTGCAAGCACTTTTTGGGCATGAATATCCGGTGAGTTGATATTCTCATTACTTATTTGCCGTGGA>7574

                  *         *         *         *         *         *         *         *         *         *
7565>TAGTATAATCATGAGTAATTTTAAAGCTCAATACTTGTACTATTCACTTCAATCAAGTGGTGTTTCACATCTTTGACTATCTCTTTTGAAATTGTTGATT>7664
7598>TAGTATAATCATGAGTAATTTTAAAGCTCAATACTTGTACTATTCACTTCAATCAAGTGGTGTTTCACATCTTTGACTATCTCTTTTGAAATTGTTGATT>7697
7554>TAGTATAATCATGAGTAATTTTAAAGCTCAATACTTGTACTATTCACTTCAATCAAGTGGTGTTTCACATCTTTGACTATCTCTTTTGAAATTGTTGATT>7653
7536>TAGTATAATCATGAGTAATTTTAAAGCTCAATACTTGTACTATTCACTTCAATCAAGTGGTGTTTCACATCTTTGACTATCTCTTTTGAAATTGTTGATT>7635
7575>TAGTATAATCATGAGTAATTTTAAAGCTCAATACTTGTACTATTCACTTCAATCAAGTGGTGTTTCACATCTTTGACTATCTCTTTTGAAATTGTTGATT>7674

                  *         *         *         *         *         *         *         *         *         *
7665>TTTGGTGTATCACATCTGCTGAAATGTTATCATATGTTTCTTTTCTGCAGGTTGGACTGCTAGATGAAGAGAAAATACTTCCATTGGGGAAGGATATCAC>7764
7698>TTTGGTGTATCACATTTGCTGAAATGTTATCATATGTTTCTTTTCTGCAGGTTGGACTGCTAGATGAAGAGAAAATACTTCCATTGGGGAAGGATATCAC>7797
7654>TTTGGTGTATCACATTTGCTGAAATGTTATCATATGTTTCTTTTCTGCAGGTTGGACTGCTAGATGAAGAGAAAATACTTCCATTGGGGAAGGATATCAC>7753
7636>TTTGGTGTATCACATTTGCTGAAATGTTATCATATGTTTCTTTTCTGCAGGTTGGACTGCTAGATGAAGAGAAAATACTTCCATTGGGGAAGGATATCAC>7735
7675>TTTGGTGTATCACATCTGCTGAAATGTTATCATATGTTTCTTTTCTGCAGGTTGGACTGCTAGATGAAGAGAAAATACTTCCATTGGGGAAGGATATCAC>7774
```

```
              *         *         *         *         *         *         *         *         *         *
7765>TGCTGTTGGCCTTGGCAGTTTAAATAATGGAGTTGTTGAAATAAAGTCATGCAAAGATCTACCATATTTTTTGTAAGGCCTAAGATTTTGTTTTGGTGGC>7864
7798>TGCTGTTGGCCTTGGCAGTTTAAATAATGGAGTTGTTGAAATAAAGTCATGCAAAGATCTACCATATTTTTTGTAAGGCCTAAGATTTTGTTTTGGTGGC>7897
7754>TGCTGTTGGCCTTGGCAGTTTAAATAATGGAGTTGTTGAAATAAAGTCATGCAAAGATCTACCATATTTTTTGTAAGGCCTAAGATTTTGTTTTGGTGGC>7853
7736>TGCTGTTGGCCTTGGCAGTTTAAATAATGGAGTTGTTGAAATAAAGTCATGCAAAGATCTACCATATTTTTTGTAAGGCCTAAGATTTTGTTTTGGTGGC>7835
7775>TGCTGTTGGCCTTGGCAGTTTAAATAATGGAGTTGTTGAAATAAAGTCATGCAAAGATCTACCATATTTTTTGTAAGGCCTAAGATTTTGTTTTGGTGGC>7874

              *         *         *         *         *         *         *         *         *         *
7865>GCATTAATATGAGTGATACTGAATTTCGTAGTTTTCCGCTAAAGTTCATTATCTGAGTGCATATATCTCGGTAAATATTCATAAGTGAATTCAGCCTGTG>7964
7898>GCATTAATATGAGTGATACTGAATTTCGTAGTTTTCCGCTAAAGTTCATTATCTGAGTGCATATATCTCGGTAAATATTCATAAGTGAATTCAGCCTGTG>7997
7854>GCATTAATATGAGTGATACTGAATTTCGTAGTTTTCCGCTAAAGTTCATTATCTGAGTGCATATATCTCGGTAAATATTCATAAGTGAATTCAGCCTGTG>7953
7836>GCATTAATATGAGTGATACTGAATTTCGTAGTTTTCCGCTAAAGTTCATTATCTGAGTGCATATATCTCGGTAAATATTCATAAGTGAATTCAGCCTGTG>7935
7875>GCATTAATATGAGTGATACTGAATTTCGTAGTTTTCCGCTAAAGTTCATTATCTGAGTGCATATATCTCGGTAAATATTCATAAGTGAATTCAGCCTGTG>7974

              *         *         *         *         *         *         *         *         *         *
7965>GACGTTCTGCTTATGGTTTTGTTCTCTTTTGTAGATCTGACTTGAGCAAAGATCAGATGATAGTGGATCTTTCCATCAAAACGAAAATTCTGTTTTGGGG>8064
7998>GACGTTCTGCTTATGGTTTTGTTCTCTTTTGTAGATCTGACTTGAGCAAAGATCAGATGATAGTGGATCTTTCCATCAAAACGAAAATTCTGTTTTGGGG>8097
7954>GACGTTCTGCTTATGGTTTTGTTCTCTTTTGTAGATCTGACTTGAGCAAAGATCAGATGATAGTGGATCTTTCCATCAAAACGAAAATTCTGTTTTGGGG>8053
7936>GACGTTCTGCTTATGGTTTTGTTCTCTTTTGTAGATCTGACTTGAGCAAAGATCAGATGATAGTGGATCTTTCCATCAAAACGAAAATTCTGTTTTGGGG>8035
7975>GACGTTCTGCTTATGGTTTTGTTCTCTTTTGTAGATCTGACTTGAGCAAAGATCAGATGATAGTGGATCTTTCCATCAAAACGAAAATTCTGTTTTGGGG>8074

              *         *         *         *         *         *         *         *         *         *
8065>TGGTGTTGCTCTCGGTTCAATGTCAGTTGGGATCCTTGGCTATGCAGTTGTGCGGTATGTTGGTTTGACTTATCTGTCCATTTATTATGTTACTTCTGTG>8164
8098>TGGTGTTGCTCTCGGTTCAATGTCAGTTGGGATCCTTGGCTATGCAGTTGTGCGGTATGTTGGTTTGACTTATCTGTCCATTTATTATGTTACTTCTGTG>8197
8054>TGGTGTTGCTCTCGGTTCAATGTCAGTTGGGATCCTTGGCTATGCAGTTGTGCGGTATGTTGGTTTGACTTATCTGTCCATTTATTATGTTACTTCTGTG>8153
8036>TGGTGTTGCTCTCGGTTCAATGTCAGTTGGGATCCTTGGCTATGCAGTTGTGCGGTATGTTGGTTTGACTTATCTGTCCATTTATTATGTTACTTCTGTG>8135
8075>TGGTGTTGCTCTCGGTTCAATGTCAGTTGGGATCCTTGGCTATGCAGTTGTGCGGTATGTTGGTTTGACTTATCTGTCCATTTATTATGTTACTTCTGTG>8174

              *         *         *         *         *         *         *         *         *         *
8165>AACTGCATTTTGTTAATTATGGTTGACTTTAGAATATTTTGAAACAAATGGATTTTCCTGGTTTGATAATCTGCCTTCATGCGGAAATGATTTGAAATAA>8264
8198>AACTGCATTTTGTTAATTATGGTTGACTTTAGAATATTTTGAAACAAATGGATTTTCCTGGTTTGATAATCTGCCTTCATGCGGAAATGATTTGAAATAA>8297
8154>AACTGCATTTTGTTAATTATGGTTGACTTTAGAATATTTTGAAACAAATGGATTTTCCTGGTTTGATAATCTGCCTTCATGCGGAAATGATTTGAAATAA>8253
8136>AACTGCATTTTGTTAATTATGGTTGACTTTAGAATATTTTGAAACAAATGGATTTTCCTGGTTTGATAATCTGCCTTCATGCGGAAATGATTTGAAATAA>8235
8175>AACTGCATTTTGTTAATTATGGTTGACTTTAGAATATTTTGAAACAAATGGATTTTCCTGGTTTGATAATCTGCCTTCATGCGGAAATGATTTGAAATAA>8274

              *         *         *         *         *         *         *         *         *         *
8265>TACTATTAGTAATTTTGGGTGATTAAAGACATAGTTGTATTGGGGGCAATTACACACTGCTGATTTTCAGGAACTGGAATAAGTGGAAACAGTGGAAGCT>8364
8298>TACTATTAGTAATTTTGGGTGATTAAAGACATAGTTGTATTGGGGGCAATTACACACTGCTGATTTTCAGGAACTGGAATAAGTGGAAACAGTGGAAGCT>8397
8254>TACTATTAGTAATTTTGGGTGATTAAAGACATAGTTGTATTGGGGGCAATTACACACTGCTGATTTTCAGGAACTGGAATAAGTGGAAACAGTGGAAGCT>8353
8236>TACTATTAGTAATTTTGGGTGATTAAAGACATAGTTGTATTGGGGGCAATTACACACTGCTGATTTTCAGGAACTGGAATAAGTGGAAACAGTGGAAGCT>8335
8275>TACTATTAGTAATTTTGGGTGATTAAAGACATAGTTGTATTGGGGGCAATTACACACTGCTGATTTTCAGGAACTGGAATAAGTGGAAACAGTGGAAGCT>8374

              *         *         *         *         *         *         *         *         *         *
8365>ACAGAGGCAGCTCCAGCAACAAAGGCAAGCAGTGAGTGATGTTGAACCTCAGGTGGATGATGAGATTGAAGATGTTCCAGATGGACAATTATGTGTTATA>8464
8398>ACAGAGGCAGCTCCAGCAACAAAGGCAAGCAGTGAGTGATGTTGAACCTCAGGTGGATGATGAGATTGAAGATGTTCCAGATGGACAATTATGTGTTATA>8497
8354>ACAGAGGCAGCTCCAGCAACAAAGGCAAGCAGTGAGTGATGTTGAACCTCAGGTGGATGATGAGATTGAAGATGTTCCAGATGGACAATTATGTGTTATA>8453
8336>ACAGAGGCAGCTCCAGCAACAAAGGCAAGCAGTGAGTGATGTTGAACCTCAGGTGGATGATGAGATTGAAGATGTTCCAGATGGACAATTATGTGTTATA>8435
8375>ACAGAGGCAGCTCCAGCAACAAAGGCAAGCAGTGAGTGATGTTGAACCTCAGGTGGATGATGAGATTGAAGATGTTCCAGATGGACAATTATGTGTTATA>8474

              *         *         *         *         *         *         *         *         *         *
8465>TGTCTGATGAGGAGAAGGCGTTCTGTCTTCATCCCCTGTGGGCATCTTGTGTGTTGCCAAGGGTGTGCCATATCAGTTGAACGTGAAGTGGCACCTAAAT>8564
8498>TGTCTGATGAGGAGAAGGCGTTCTGTCTTCATCCCCTGTGGGCATCTTGTGTGTTGCCAAGGGTGTGCCATATCAGTTGAACGTGAAGTGGCACCTAAAT>8597
8454>TGTCTGATGAGGAGAAGGCGTTCTGTCTTCATCCCCTGTGGGCATCTTGTGTGTTGCCAAGGGTGTGCCATATCAGTTGAACGTGAAGTGGCACCTAAAT>8553
8436>TGTCTGATGAGGAGAAGGCGTTCTGTCTTCATCCCCTGTGGGCATCTTGTGTGTTGCCAAGGGTGTGCCATATCAGTTGAACGTGAAGTGGCACCTAAAT>8535
8475>TGTCTGATGAGGAGAAGGCGTTCTGTCTTCATCCCCTGTGGGCATCTTGTGTGTTGCCAAGGGTGTGCCATATCAGTTGAACGTGAAGTGGCACCTAAAT>8574

              *         *         *         *         *         *         *         *         *         *
8565>GCCCTGTTTGTCGTCAGGAGATTCGAAATTCAGTGCGGATTTTTGAATCATGATCAGACACCAGCTGAATGTTCTGTTCACTCCTGCGGCAGGAAACTTT>8664
8598>GCCCTGTTTGTCGTCAGGAGATTCGAAATTCAGTGCGGATTTTTGAATCATGATCAGACACCAGCGGAATGTTCTGTTCACTCCTGCGGCAGGAAACTTT>8697
8554>GCCCTGTTTGTCGTCAGGAGATTCGAAATTCAGTGCGGATTTTTGAATCATGATCAGACACCAGCGGAATGTTCTGTTCACTCCTGCGGCAGGAAACTTT>8653
8536>GCCCTGTTTGTCGTCAGGAGATTCGAAATTCAGTGCGGATTTTTGAATCATGATCAGACACCAGCGGAATGTTCTGTTCACTCCTGCGGCAGGAAACTTT>8635
8575>GCCCTGTTTGTCGTCAGGAGATTCGAAATTCAGTGCGGATTTTTGAATCATGATCAGACACCAGCTGAATGTTCTGTTCACTCCTGCGGCAGGAAACTTT>8674

              *         *         *         *         *         *         *         *         *         *
8665>AACACCTGCTAATGAGAAGAATGTGCTTTTTGTTTTGATAAGATATGGAATATGGAATTTTTGGGAAAACTTGTCTTGCTCTTGTTTCCTGTGAACTTAT>8764
8698>AACACCTGCTAATGAGAAGAATGTGCTTTTTGTTTTGATAAGATATGGAATATGGAATTTTTGGGAAAACTTGTCTTGCTCTTGTTTCCTGTGAACTTAT>8797
8654>AACACCTGCTAATGAGAAGAATGTGCTTTTTGTTTTGATAAGATATGGAATATGGAATTTTTGGGAAAACTTGTCTTGCTCTTGTTTCCTGTGAACTTAT>8753
8636>AACACCTGCTAATGAGAAGAATGTGCTTTTTGTTTTGATAAGATATGGAATATGGAATTTTTGGGAAAACTTGTCTTGCTCTTGTTTCCTGTGAACTTAT>8735
8675>AACACCTGCTAATGAGAAGAATGTGCTTTTTGTTTTGATAAGATATGGAATATGGAATTTTTGGGAAAACTTGTCTTGCTCTTGTTTCCTGTGAACTTAT>8774
```

```
              *         *         *         *         *         *         *         *         *         *
8765>GACTTTATCTGGGGTAGATAATTGGACATGGCTTAAATTGTTTGGTCGAGGTTAATTCGTAGCTTCCTCACTTTCTCACTCTGCCAGGTCTTTTTGGGAA>8864
8798>GACTTTATCTGGGGTAGATAATTGGACATGGCTTAAATTGGTTGGTCGAGGTTAATTCGTAGCTTCCTCACTTTCTCACTCTGCCAGGTCTTTTTGGGAA>8897
8754>GACTTTATCTGGGGTAGATAATTGGACATGGCTTAAATTGGTTGGTCGAGGTTAATTCGTAGCTTCCTCACTTTCTCACTCTGCCAGGTCTTTTTGGGAA>8853
8736>GACTTTATCTGGGGTAGATAATTGGACATGGCTTAAATTGGTTGGTCGAGGTTAATTCGTAGCTTCCTCACTTTCTCACTCTGCCAGGTCTTTTTGGGAA>8835
8775>GACTTTATCTGGGGTAGATAATTGGACATGGCTTAAATTGTTTGGTCGAGGTTAATTCGTAGCTTCCTCACTTTCTCACTCTGCCAGGTCTTTTTGGGAA>8874

              *         *         *         *         *         *         *         *         *         *
8865>ACCTTGCAAGATTTTCCAACTAATCCAGTTGCAGTGTACATAAATACTTAATTTTGCCTTACACCTATAGAATGAACTCTGATATGCTCTTGTTCATATC>8964
8898>ACCTTGCAAGATTTTCCAACTAATCCAGTTGCAGTGTACATAAATACTTAATTTTGCCTTACACCTATAGAGTGAACTCTGATATGCTCTTGTTCATTTC>8997
8854>ACCTTGCAAGATTTTCCAACTAATCCAGTTGCAGTGTACATAAATACTTAATTTTGCCTTACACCTATAGAGTGAACTCTGATATGCTCTTGTTCATTTC>8953
8836>ACCTTGCAAGATTTTCCAACTAATCCAGTTGCAGTGTACATAAATACTTAATTTTGCCTTACACCTATAGAGTGAACTCTGATATGCTCTTGTTCATTTC>8935
8875>ACCTTGCAAGATTTTCCAACTAATCCAGTTGCAGTGTACATAAATACTTAATTTTGCCTTACACCTATAGAATGAACTCTGATATGCTCTTGTTCATATC>8974

              *         *         *         *         *         *         *         *
8965>ATGCTGCTTTCTAGTTAAAGGTAATCTCACGAGTAAACCTGTAAATCCTACATTAAAAAGTAAAAACTGATGCTGGTATTCAG>9047
8998>ATGCTGCTTTCTAGTTAAAGGTAATCTCACGAGTAAACCTGTAAATCCTACATTAAAAAGTAAAAA~~~~~~~~~~~~~~~~~>9063
8954>ATGCTGCTTTCTAGTTAAAGGTAATCTCACGAGTAAACCTGTAAATCCTACATTAAAAAGTAAAAA~~~~~~~~~~~~~~~~~>9019
8936>ATGCTGCTTTCTAGTTAAAGGTAATCTCACGAGTAAACCTGTAAATCCTACATTAAAAAGTAAAAA~~~~~~~~~~~~~~~~~>9001
8975>ATGCTGCTTTCTAGTTAAAGGTAATCTCACGAGTAAACCTGTAAATCCTACATTAAAAAGTAAAAACTGATGCTGGTATTCAG>9057
```
